# Twists, Turns, and Spins: Copper-Guided Electronic Communication in Sulfur-Bridged Trimetallic Complexes

**DOI:** 10.1021/acsorginorgau.6c00057

**Published:** 2026-07-31

**Authors:** Akhil K. Singh, Dakota D. Jones, Aruzhan Abdikaiym, Naushad Ahmed, Sarnali Sanfui, Joseph H. Reibenspies, Yisong Guo, Brad S. Pierce, Kim R. Dunbar, Michael B. Hall, Marcetta Y. Darensbourg

**Affiliations:** † Department of Chemistry, 14736 Texas A&M University, College Station, Texas 77843, United States; ‡ Department of Chemistry & Biochemistry, 8059 University of Alabama, Tuscaloosa, Alabama 35487, United States; § Department of Chemistry, 6612 Carnegie Mellon University, Pittsburgh, Pennsylvania 15213, United States

**Keywords:** Trimetallic complexes, electronic communication, iron-nitrosyl, vibrational spectroscopy, metallodithiolate ligand

## Abstract

Sulfur-bridged multimetallic complexes provide a platform for probing long-range electronic communication relevant to metalloproteins and molecular electronic materials. Trimetallic complexes of the type (MN_2_S_2_)_2_Cu (M = [FeNO]^2+^ or [VO]^2+^), in which these two redox-active *S* = 1/2 spin probes are linked by a diamagnetic Cu­(I) center in a tetrahedral S_4_ coordination environment, are developed. Structural analysis shows that the Cu­(I) bridge enforces a twisted arrangement of the two MN_2_S_2_ units, in contrast to square-planar NiS_4_-bridged analogues, [ QuirozM., ,  Chem. Sci. 2023, 14, 9167–9174].10.1039/d3sc01546gPMC1046628537655023 leading to distinct metal–ligand orbital alignment and sulfur-mediated bonding pathways. Despite a large interspin separation of approximately 6 Å between the spin probes, magnetic susceptibility measurements reveal appreciable ferromagnetic exchange coupling between these paramagnetic centers, although weaker than the coupling observed for the d^8^ (M′ = Ni­(II), Pd­(II), and Pt­(II))-bridged stair-step complexes. These differences are attributed to the contrasting geometries of the CuS_4_ and M′S_4_ cores and their impact on superexchange through the bridging sulfurs. In addition to the solid-state X-ray crystallography, this new class of trimetallics was characterized by multiple methods including electrochemistry, vibrational spectroscopy, magnetic susceptibility, EPR, Mössbauer, and DFT computations. Redox and vibrational studies further probe electronic communication between the FeNO units: ν­(NO) stretching frequency indicates that one-electron reduction is largely localized on a single FeNO center, whereas two-electron reduction leads to electron delocalization across both units. Together, these results demonstrate that copper-imposed geometric distortion modulates sulfur-mediated spin coupling and electron delocalization, providing design principles for multimetallic, spin-active architectures.

## Graphical abstract

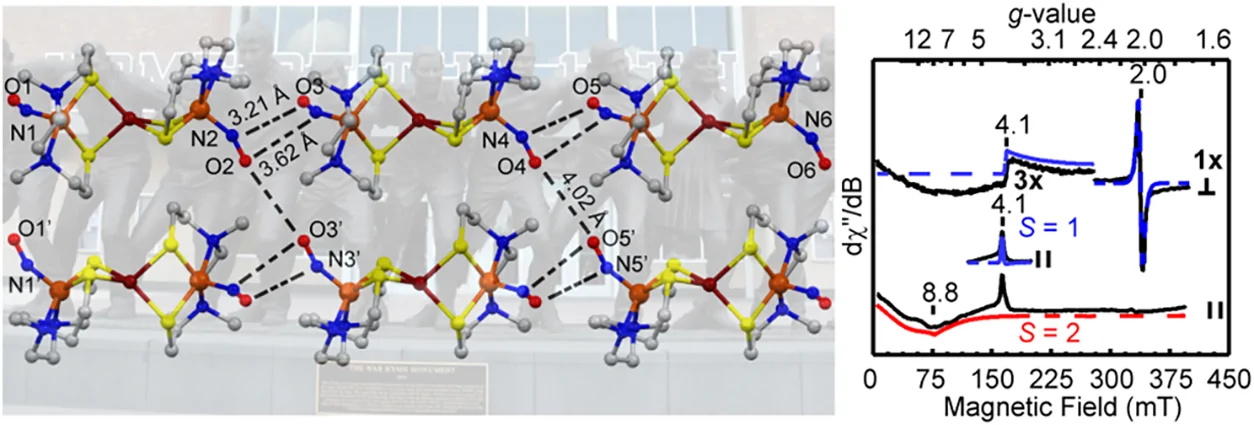

## Introduction

In recognition of the important roles that sulfur plays in the construction of catalytically active sites in metallo-biochemistry,^1–4^ as well as the quantum properties expected in solid-state extended metal sulfides,^5–7^ we have developed synthons capable of linking metals by sulfur in trimetallic complexes, i.e., MS_2_M′S_2_M. Their thorough characterization by a suite of tools well known to physical inorganic chemists offers prospects of relating fundamental properties at the atomic level to useful applications that require charge and spin coupling across metal sites.^8–11^

We have thus harnessed the well-known aggregative ability of thiolate sulfurs to produce a series of tri- and tetrametallic complexes wherein paramagnetic [Fe­(NO)]^2+^ or [V≡O] ^2+^ units, themselves securely held in an N_2_S_2_ ligand field, are connected through robust sulfur bridges to a central metal with discrete spatial requirements.^12^ Serving as a foundation for this approach has been square planar, d^8^ Ni­(II), Pd­(II) and Pt­(II), or a Ni_2_S_2_ cluster (Chart 1B,C).^13,14^ Taking advantage of the magnetic properties of spin-labeled multimetallics, we have developed a reliable synthetic approach for such systems that informs on long-range (6–8 Å) coupling between the distal metals in the “stair-step topologies”.^13^ With the [(N_2_S_2_)­Fe­(NO)] metallodithiolate, *S* = 1/2, spin probe ligand, magnetic coupling constant (*J* values) range from −124 to −3 cm^–1^, M = Pt, Pd, Ni. The bridging Ni_2_S_2_ cluster also shows antiferromagnetic coupling with a value, −54 cm^–1^, close to that of Pd^2+^, −23 cm^–1^.^14^

**Chart 1:**
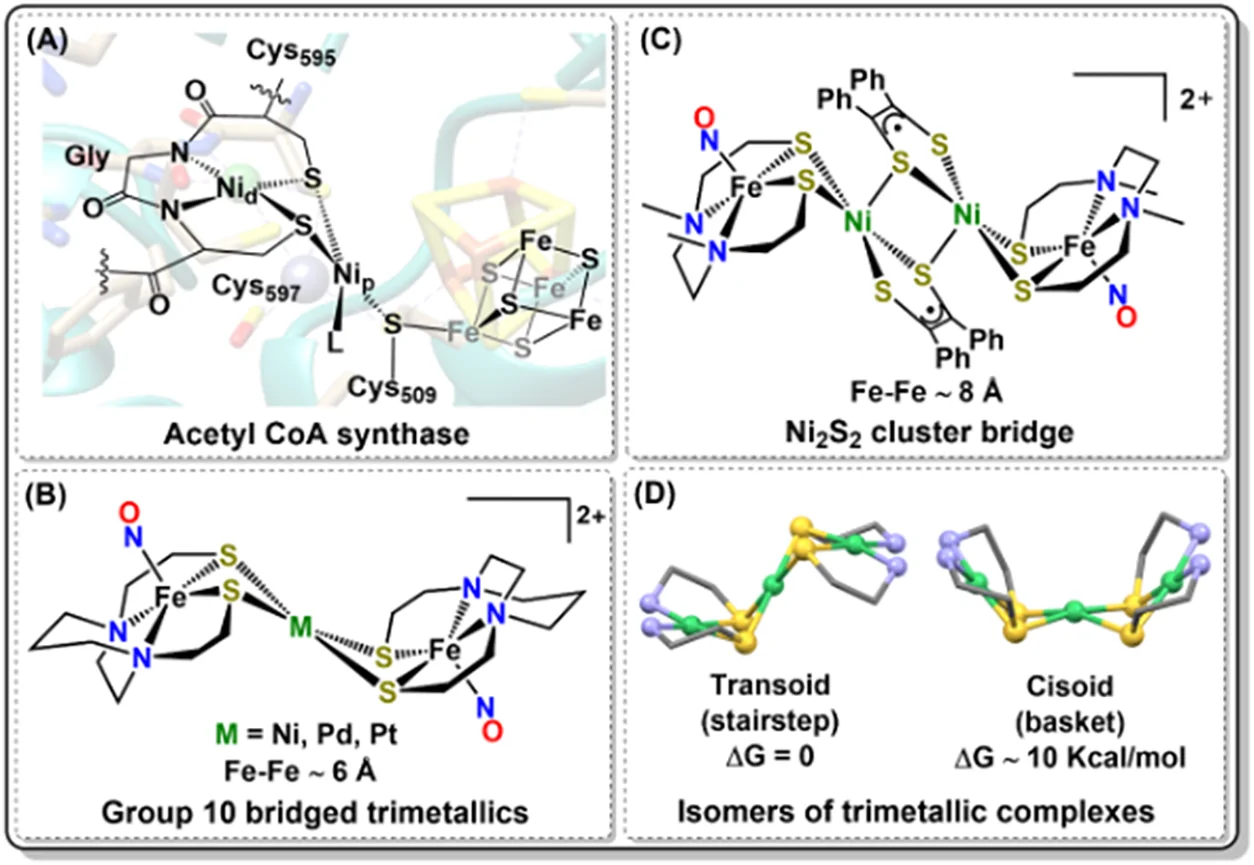
Structures of Selected Thiolate-Rich Trimetallic Complexes Featuring Various Donor Types and Coordination Geometries[Fn c1fn1]

a (A) Acetyl-CoA synthase (ACS) active site. (B) Stair-step trimetallic complex based on the [(N_2_S_2_)­Fe­(NO)] metallo-ligand. (C) Ni_2_S_2_ cluster-bridged complex. (D) Isomers of thiolate-bridged trimetallic complexes.

In an analogous Group 10 series, (N_2_S_2_)­[V≡O] derivatives display much weaker coupling and ferromagnetic behavior.^15^ The better orbital alignment of the highly delocalized (N_2_S_2_)­Fe­(NO) combined with the bridging group 10 metals in the stair-step topology accounts for the difference in magnitude and type of magnetic coupling. That is, the contraction of the spin-orbital on the vanadyl cation and its localization toward the vanadium­(+4) center in [V≡O]^2+^ accounts for its lesser involvement and effect on the bridging metal ion.

The ν­(NO) vibration is a sensitive monitor of charge distribution in the redox levels suggested from solution cyclic voltammetry; FTIR and 2-D FTIR results distinguished charge centers in three redox levels of diiron trinitrosyl complexes.^16,17^ Thus, the noninnocence of the (N_2_S_2_)­Fe­(NO) metallodithiolate ligand in terms of accessibility to redox levels has been established. Serving as an “innocent” ligand counterpart, the (N_2_S_2_)Ni derivative can be compared to diphosphines for which group 10 metals readily form regular square-planar [M­(diphos)_2_]^2+^ derivatives.^18^ Utilization of such phosphines for holding substrates for activation by M requires careful synthetic modifications in the secondary sphere of the complex.^19^ The added structural variety from the inner sphere extra lone pair on sulfur accounts for MS_2_M′ hinges and the “stair-step” topology of the sets of trimetallics, exemplified in Chart 1B. This *transoid* metallodithiolate isomer is favored by electrostatic repulsion between sulfur lone pairs as well as steric repulsion by the N to N and S to S hydrocarbon linkers by *ca*. 10 kcal/mol over the *cisoid* isomer.^20,21^ The basket-like topology of the latter uncommon isomer is expected to hold, and perhaps activate, a substrate.^21^ In fact, the most accessible basket-type trimetallic derived from metallodithiolate ligands has been found to exist in the presence of a Mo-NO or Cr-NO as the center of the basket floor. In this case, the π-accepting ability of the M-NO center works in synergy to overcome the S-lone pair repulsions and assist in the dispensation of excess S-negative charge.^21,22^ In general, the geometrical preferences of the central metal are accommodated within the topologies that comprise these hydrocarbon-linked N_2_S_2_ binding sites to a centroid metal ion in the sulfur-bridged trimetallic constructions.^23^

Sulfur-bridged di- and multimetallics are common constructions that nature has strategically used in metalloproteins.^24–26^ In fact, extensive aggregation at sulfur sites has been a vexing impediment to the progress of synthetic systems designed to mimic the biological redox catalysis properties of sulfur-containing metalloproteins, requiring heroic efforts to control the stoichiometry of the molecular metal sulfides.^27^ The simple Cys-X-Cys tripeptide motif, which our hydrocarbon-linked N_2_S_2_ ligand nominally mimics, provides two peptide-derived nitrogen atoms and two cysteinyl sulfur donors to create an ideal tetradentate N_2_S_2_ binding pocket amenable to the tight binding site for Ni^2+^, in acetyl-CoA synthase (Chart 1A).^28,29^ The second nickel is known to be labile and competitive with other metal ions such as Cu and Zn, creating undesirable heterogeneity in enzyme preparations.^30^ Note that this second nickel is sandwiched between the N_2_S_2_Ni and an iron-sulfur cluster, creating a trimetallic chain of NiS_2_ Ni’SFe-Fe_3_S_3_ cluster that suggests involvement of the whole construction in the redox catalytic mechanism of the enzyme.^30,31^

In the “simple” trimetallic systems thus far developed, the central bridging metal was found to facilitate efficient electronic communication between the terminal metal ions due to favorable orbital, parallel, alignment, and energetic overlap.^13,15^ We posited that placement of the distal spin probes at an angle should modulate the extent of orbital alignment by the central metal and act as a test of our hypothesis.

Since copper­(I) is known to stabilize in a tetrahedral coordination sphere,^32^ we chose to examine it as a connecting metal in this trimetallic complex system while maintaining the diamagnetic nature of the metal, analogous to group 10 divalent cations. As observed in biological^33–35^ and synthetic^36^ systems, the ability of copper to access multiple oxidation states and adopt varied coordination geometries, especially combined with the equally versatile stereodirecting sulfur donors, creates a fascinating choice to place spin probes at different orientations. Thus, the two metallodithiolates have been applied in the simple synthesis in Scheme 1. The spin-density plots for the metallodithiolate ligands are also given with accompanying charge and spin values showing the differences in the metallodithiolate ligands.^37^ The iron center in (A) shows major delocalization within the Fe­(NO) and over the sulfur atoms. In contrast, the vanadyl derivative displays localization of charge and spin. Extensive characterization of the products is given below.

**Scheme 1:**
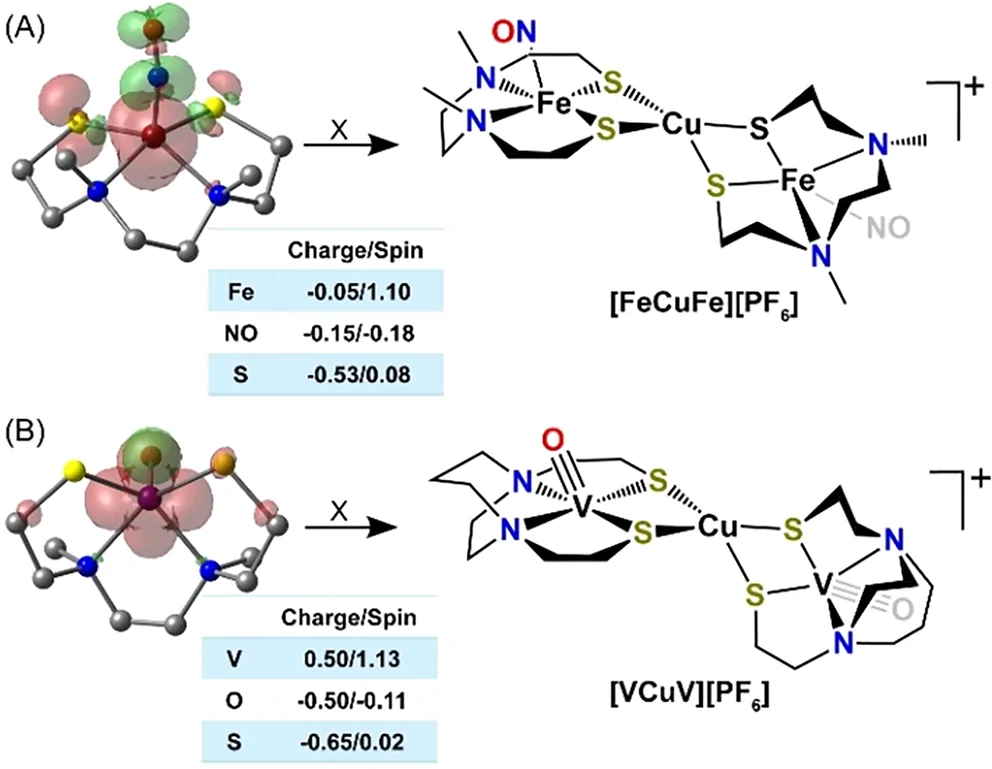
Synthesis and Structures of the Copper­(I)-Bridged Trimetallics with (N_2_S_2_)­Fe­(NO) and (N_2_S_2_)­V­(O) metalloligands^a^

a Spin density plots^37^ (isovalue = 0.003 au) showcasing significant spin delocalization onto the dithiolates in (N_2_S_2_)­Fe­(NO) complexes; synthon X= [Cu­(CH_3_CN)_4_]^+^ with PF_6_^–^ or BF_4_^–^, but localized on V in the (N_2_S_2_)­V­(O) analog.

## Results and Discussion

Procedures for the preparation of N_2_S_2_ ligands, their metalation (MN_2_S_2_) and nitrosylated complexes, were reported earlier (see Experimental Section for detailed methodology).^38–40^ To an acetonitrile solution of [(N_2_S_2_)­Fe­(NO)] at 22 °C, 0.5 equiv of portions of copper­(I) salts, BF_4_^–^ or PF_6_^–^, dissolved in acetonitrile were added dropwise. The reaction was monitored by the color changes from green to brown, accompanied by a shift in the ν­(NO) IR stretch of the metallodithiolate free ligand, [(N_2_S_2_)­Fe­(NO)] at ca. 1665 cm^–1^ to a single stretch at ca. 1694 cm^–1^ in the products (Figure S1). The observed shift to a higher wavenumber is attributed to a drain of electron density from the Fe­(NO) centers upon coordination of exogenous metal cations to the thiolate sulfurs, diminishing the π-back-donation from the Fe centers to the nitrosyl ligands and increasing the ν­(NO) stretching frequency (Scheme 1).^13^ The reaction mixture was stirred for 30 min under N_2_, and further purification of crude product yielded a dark brown powder. In a similar approach, a 0.5 equiv of copper salts dissolved in acetonitrile was added dropwise into an acetonitrile solution of [(N_2_S_2_)­(V≡O)] under nitrogen, giving the VO complexes in good yields.

The electrospray ionization mass spectrometry (ESI-MS) showed intense parent peaks at *m*/*z* 646.976 for **[FeCuFe]** ^**+**^ (Figure S2) and at 632.988 for **[VCuV]** ^**+**^ (Figure S3); experimental isotopic distribution patterns well match the calculated patterns. The UV–visible spectrum of **[FeCuFe]­[PF**_**6**_ **]** in acetonitrile displays three absorption bands at 364, 483, and 650 nm, while **[FeCuFe]­[BF**_**4**_ **]** in acetonitrile has absorption bands located at 364, 490, and 658 nm (Figure S4). The band around 650 nm is assigned as a thiolate-S to Fe charge transfer, similar to the characteristic ∼710 nm band observed for NO-deactivated, iron-containing nitrile hydratase.^41^ A charge transfer transition is observed in the ∼220–350 nm range. The 483 nm band is interpreted as thiolate-S to Cu charge transfer, comparable to the transition seen in various Cupredoxins.^42–44^ In contrast, the band at ∼650 nm is absent in the **[VCuV]­[BF**_**4**_ **]**; instead, the S to Cu charge transfer band appears near 445 nm, and charge transfer bands are observed in the ∼250–290 nm range (Figure S5).

### Single-Crystal X-ray Diffraction Analysis

Single crystals of **[FeCuFe]­[PF**_**6**_ **], [FeCuFe]­[BF**_**4**_ **], [VCuV]­[PF**_**6**_ **]**, and **[VCuV]­[BF**_**4**_ **]** were obtained by vapor diffusion of Et_2_O into CH_3_CN solutions of the materials at room temperature under N_2_ (Tables S1 and S2). Figures 1 & S13–S16 displays the SC-XRD-derived structures of the complexes; molecular packing diagrams from their respective unit cells are given in Figures S6–S11.

**Figure 1:**
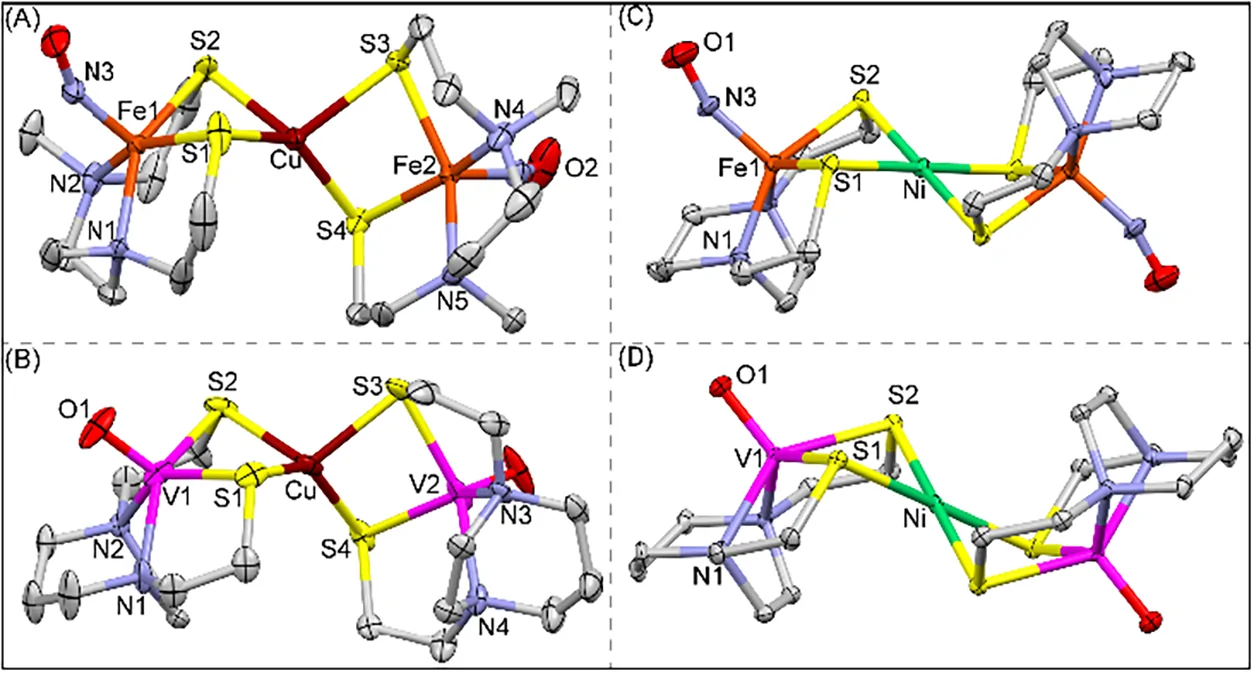
Abbreviated ellipsoid plots (50% thermal contour) at 100 K, for Cu­(I) (A, B) and Ni­(II) complex cations (C, D) with Fe­(NO) vs V≡O metallodithiolate ligands. Complexes containing Ni­(II) are generated by symmetry about an inversion center at the nickel site.

The structural analyses revealed trimetallic skeleton arrangements as expected for sulfur-hinged conformations. All MCuM complexes crystallize in the monoclinic P21/c space group except **[VCuV]­[PF**_**6**_ **]** in the P21/n space group, containing four molecules per unit cell (Figures S6, S9, and S11). The **[FeM′Fe]** ^**+**^ complexes have discrete Fe centers, each in a five-coordinate square pyramidal geometry at N_2_S_2_ thiolates with the Fe displaced from the N_2_S_2_ best plane and toward the NO apex. Each (N_2_S_2_)­Fe­(NO), or (N_2_S_2_)­V­(O), core is bridged by a tetrahedral CuS_4_ unit, twisting the N_2_S_2_ planes, and yielding a Td angle, defined by the intersection of two CuS_2_ best planes of the metalloligands, of approximately 87°. Unlike the previously reported **[FeM′Fe]** ^**2+**^ stair-step complexes bridged by group 10 metals **(M = Ni** ^**2+**^, **Pd** ^**2+**^, **Pt** ^**2+**^ **)**, where both (N_2_S_2_)­Fe­(NO) units exhibited parallel N_2_S_2_ planes and equivalent metrical parameters^13^ (Figure 1C,D), the copper-bridged complexes show slight differences; however, it is considered insignificant to our general discussion. The Fe–N–O angles span 150.6° to 156.1°, similar to the unbound metallodithiolate ligand^45^ but slightly less linear compared to those in the **[FeM′Fe]** ^**2+**^, M = Ni^2+^, Pd^2+^, Pt^2+^ series (Tables 1 and S3).^13^ The Fe–Cu and Fe–Fe distances vary from 2.82 Å to 2.90 Å and 5.64 Å to 5.71 Å, respectively; as expected, both are shorter than the analogous metrics in **[FeM′Fe]** ^**2+**^ complexes (2.96 Å and 5.91 Å, respectively).^13^

**Table 1:** Selected Structural and Geometrical Parameters

**Complex**	**[FeCuFe] [PF**_**6**_ **]**	**[VCuV] [PF**_**6**_ **]**	**[FeNiFe] [2BF**_**4**_ **]**	**[VNiV] [2BF**_**4**_ **]**
V/Fe–N (Å)^a^	2.057(2)	2.126(5)	2.007(1)	2.112(2)
V/Fe–S (Å)^b^	2.266(1)	2.383(2)	2.228(3)	2.351(1)
**ΔN**_**2**_ **S**_**2**_^**M**^ **(Å)** ^c^	0.514	0.697	0.615	0.694
M-M′ (Å)^d^	2.894(1)	2.956(1)	2.956(2)	2.990
**∠Fe–N–O** ^e^	156.1(2)	---	165.6(1)	---
M-M (Å)^f^	5.71	5.92	5.91	5.98
**Td angle at Cu (°)** ^g^	87.4	80.7	0.00	0
**Hinge angle (°)** ^h^	72.4	63.2	73.6	82.1
	76.0	67.8		
**Interplanar angle (°)** ^i^	38.28	56.89	0.00	0
**ref.**	This work	This work	^13^	^15^

a Average distance between metals (Fe or V) and nitrogen of the N_2_S_2_ core.

b Average distance between metals (Fe or V) and sulfur from the N_2_S_2_ core.

c Average displacement of metals (Fe or V) from the least-squares plane of the N_2_S_2_ core.

d Distance between Cu or Ni and the terminal metals.

e Fe–N–O angle

f Nonbonding distance between metals in N_2_S_2_ cores.

g Angle between two tetrahedron planes (M′S_2_) formed by copper.

h The dihedral angle (θ) between the N_2_S_2_ and CuS_2_ mean planes.

i Angle between two intramolecular least-squares planes of N_2_S_2_ cores. Structural data for [FeNiFe] and [VNiV] are taken from refs 13 and 15.

In contrast to the group 10-bridged **[FeM′Fe]** ^**2+**^ stair-step complexes where two identical N_2_S_2_ units are diagonally stacked in parallel (Figure S14), having interplanar angles (dihedral angles) of zero, the two N_2_S_2_ planes in M = Cu­(I) derivatives are twisted with an interplanar angle of approximately 38°, to accommodate the copper preference for tetrahedron. A comparison of the ∠S-M-S and ∠S-M′-S angles is given in Figure S12 and Table S2, and the corresponding angles for the parent Fe­(NO)­bme-dame^45^ and (V≡O)­bme-dach^40^ are given in Figure S17.

The tetrahedral twist at copper renders each distinct molecule having C1 symmetry in the unit cell chiral; however, there is an inversion center within the unit cell. Therefore, the space group *P*2_1_/*c* is consistent with overall achirality. Inspection of **[FeCuFe]­[PF**_**6**_ **]** reveals four molecules in one unit cell (Figure 2A), which interact through NO with other molecules from neighboring unit cells, forming an alternating arrangement of discrete chains of cations flanked by rows of anions (Figure 2B). The closest F···F distance between two PF_6_ anions is 3.23 Å. The independent chains of cations show close intrachain contacts of NO ligands of 3.21 Å, as given in Figure 2C. An NO on the first iron center of one molecule interacts with the NO ligand of the second iron center of a neighboring molecule within the column, generating a linear, head-to-tail chain arrangement. There are close NO···NO contacts (4.03 Å) between the columns also.

**Figure 2:**
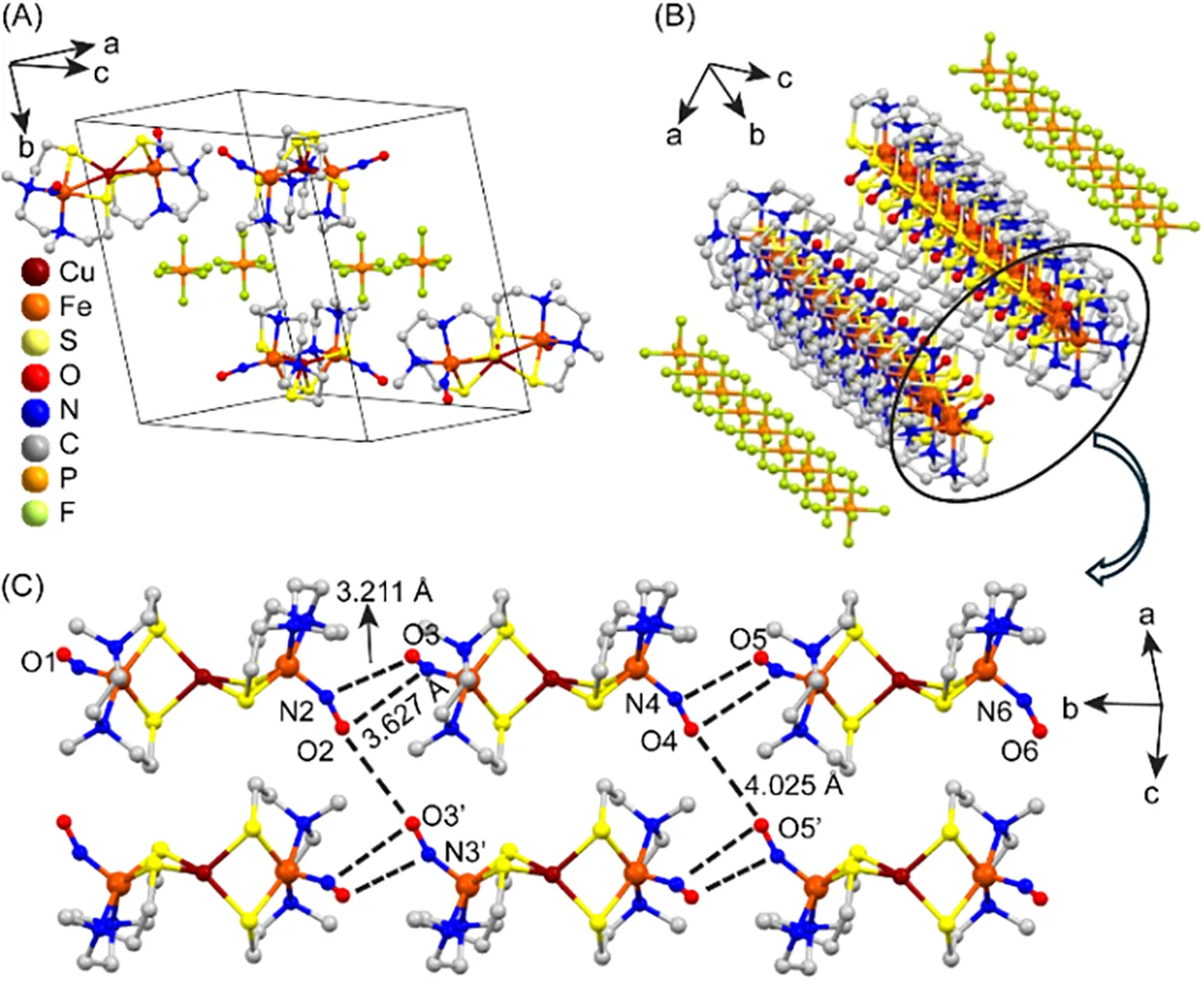
(A) Unit cell at 100 K of **[FeCuFe]­[PF**_**6**_ **]**. (B) Linear chain structures (from extended packing along the *b* axis) showing discrete columns of **[FeCuFe]** ^**+**^ and **[PF**_**6**_ **]** ^**‑**^ in head-to-tail arrangements of trimetallics due to short NO···NO contacts with two columns flanked by rows of PF_6_^–^ anions. (C) Intra- and intermolecular interactions in columns of **[FeCuFe]** ^**+**^ viewed down the *b* axis.

In the case of **[FeCuFe]­[BF**_**4**_ **]**, an acetonitrile molecule cocrystallizes and prohibits the type of intermolecular packing as shown for the PF_6_^–^ analogue (Figure S9). The vanadyl analogues do not display close intermolecular interactions. Complex **[VCuV]­[PF**_**6**_ **]** crystallizes in a monoclinic space group *P*2_1_/*n* and contains four molecules in each unit cell (Figure S11). The X-ray structure shows a five-coordinate vanadium having an oxido group at the fifth coordination position in each N_2_S_2_ unit. Each MN_2_S_2_ unit is bridged by a distorted tetrahedral Cu­(I) having an average twist of 81°, with the S–Cu distances averaging to 2.31 Å. The vanadium atom is displaced by ca. 0.70 Å above the mean N_2_S_2_ plane toward the oxido group with a V–O distance of 1.59 Å. The V···V and V···Cu separations are 5.92 Å and 2.98 Å, respectively; both are slightly longer than M-M′ and M-M distances in **FeCuFe** complexes but similar to the **FeM′Fe** and **VM′V** complexes, M = Group 10 dications.^15^ The hinge angle between N_2_S_2_ planes and CuS_2_ planes is nearly the same as the angle between the N_2_S_2_ and MS_4_ planes in the stair-step group 10-bridged trimetallic **[FeM′Fe]** ^**2+**^ and **[VM′V]** ^**2+**^ complexes. Also diverging from the group 10-bridged trimetallic complexes having C_2h_ symmetry, the two N_2_S_2_ planes in **[VCuV]­[PF**_**6**_ **]** have an interplanar angle of approximately 57°, in contrast to the **[FeCuFe]** ^+^ angle of ∼40°. The S1–V–S2 angle decreases to the same extent in both cores, from 98° in the parent (bme-dach)­V­(O) ligand (Figure S17B) to 93° in the **[VCuV]­[PF**_**6**_ **]** complex (Figure S12B). The intraligand S–Cu–S angles (∠S1–Cu–S2 and ∠S3–Cu–S4) are the same for both N_2_S_2_ units, measuring 97°; however, interligand S–Cu–S angles (∠S2–Cu–S3 and ∠S1–Cu–S4) are 110° and 138°, suggesting distortion from tetrahedral geometry.

### Magnetic Properties in the Solid State

To understand the nature and magnitude of magnetic interactions between the two spin carriers, FeNO or VO, separated by a bridging diamagnetic metal Cu^I^ (d^10^) center, we performed direct current (dc) magnetic susceptibility measurements on polycrystalline samples of **[FeCuFe]­[PF**_**6**_ **]** and **[VCuV]­[PF**_**6**_ **]** under an applied magnetic field of 10 kOe over the temperature range of 1.8 to 300 K (Figure 3). The magnetic susceptibility data were processed, corrected to Pascal’s constants,^46^ and plotted as χ_*M*_ *T* = *f*(T). The observed room-temperature χ_*M*_ *T* values of 0.96 cm^3^ K mol^–1^ and 0.73 cm^3^ K mol^–1^ for complexes **[FeCuFe]­[PF**_**6**_ **]** and **[VCuV]­[BF**_**4**_ **]** are found to be very close to the calculated value for two uncoupled electrons (*S* = 1/2, χ_*M*_ *T* = 0.76 cm^3^ K mol^–1^). The calculated μ_eff_ of 2.46 μ_B_ matches well with solution-phase magnetism from NMR experiments using the Evans method^47^ (Figure S19). Upon lowering the temperature, the χ_*M*_ *T* value remains almost constant to 45 K; below this temperature, the χ_*M*_ *T* value starts to increase and reaches the maximum value of 1.06 cm^3^ K mol^–1^ at 6 K (for **[FeCuFe]­[PF**_**6**_ **]**) and 0.77 cm^3^ K mol^–1^ at 2 K (for **[VCuV]­[PF**_**6**_ **]**). The low-temperature increase in χ_*M*_ *T* value in both cases is an indication of ferromagnetic coupling between the spin carriers mediated by the orbital overlap of the central Cu^I^ unit (see the Computational Methodology section). In the case of **[FeCuFe]­[PF**_**6**_ **]**, upon further lowering the temperature, the χ_*M*_ *T* decreases precipitously and reaches a minimum value of 0.92 cm^3^ K mol^–1^. The very low-temperature χ_*M*_ *T* profile is attributed to intermolecular antiferromagnetic interactions. In fact, the extended packing of complex **[FeCuFe]­[PF**_**6**_ **]** confirms the intermolecular close distance of ∼3.2 Å between spin carriers (see Figure 2C). In the case of **[VCuV]­[PF**_**6**_ **]**, no sudden decrease in the χ_*M*_ *T* value occurs down to 2 K, although a very weak intermolecular interaction *zJ* was used to fit the magnetic data. This result is not surprising given the longer intermolecular distance between V≡O spin carrier units, which is nearly twice that of **[FeCuFe]­[PF**_**6**_ **]**. Additionally, the delocalization of spins in the Fe-NO units supports stronger intermolecular magnetic interactions as compared to **[VCuV]­[PF**_**6**_ **]**; this is also evident from EPR spectra *(vide infra)*.

**Figure 3:**
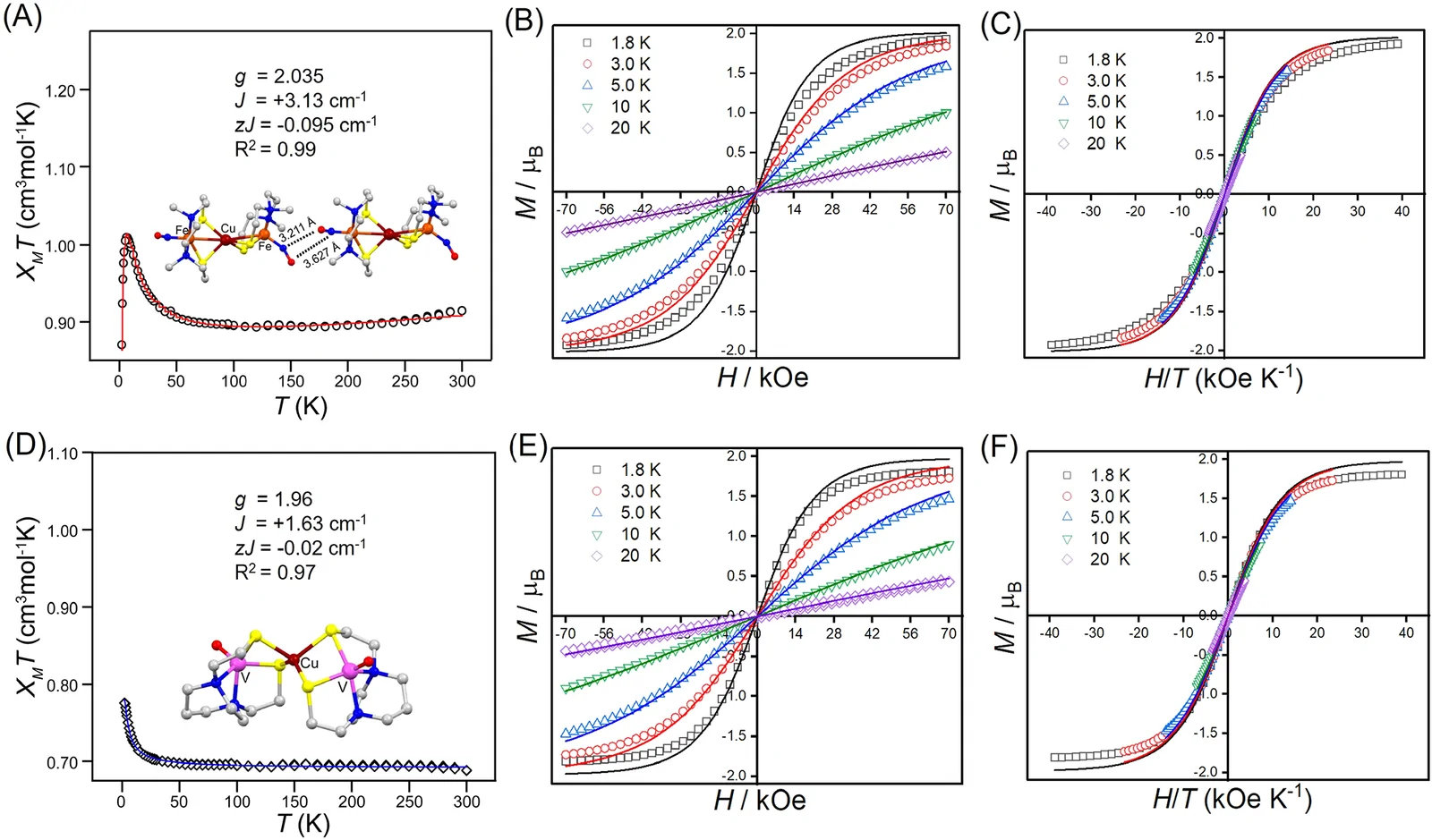
Temperature-dependent magnetic susceptibilities, magnetization, and reduced magnetization data for complexes **[FeCuFe]­[PF**_**6**_ **]** (A–C) and **[VCuV]­[PF**_**6**_ **]** (D–F) are plotted as χ_M_ = f­(T), M = f­(H), and M = f­(H/T), respectively. The solid lines/curves are the fitted data.

The field-dependent magnetization data, collected at temperatures of 1.8 to 20 K under an applied magnetic field of ±70 kOe, were plotted as M = *f*(*H*) and M = *f*(*H*/*T*). The magnetization value increases linearly with an increase in the magnetic field up to 40 kOe, after which there is no significant increase in magnetization. At 1.8 K and an applied magnetic field of ±70 kOe magnetic field, the magnetization saturation *M*_sat_ values of 1.92 μ_*B*_ and 1.81 μ_*B*_ were observed for **[FeCuFe]­[PF**_**6**_ **]** and **[VCuV]­[BF**_**4**_ **]**, respectively. The observed saturation values are slightly lower than the theoretical value of 2.0 μ_*B*_ for two spins. The reduced magnetization M = *f*(*H*/*T*) curves are superimposable, a result that suggests little to no magnetic anisotropy associated with these molecules. To estimate the nature and magnitude of spin–spin coupling *J*, we have fitted the magnetization and magnetic susceptibility data simultaneously using Hamiltonian ([Disp-formula eq1]) within the PHI program.^48^

1 $$H=\mu \mathrm{BH}\left(gS_{1}+gS_{2}\right)-2J\left(S_{1}\cdot S_{2}\right)$$

The best fit yielded *J* = +3.5 cm^–1^ with *g* = 2.035 and intermolecular interaction *zJ* = −0.11 cm^–1^ (for **[FeCuFe]­[PF**_**6**_ **]**) and *J* = +1.62 cm^–1^ with *g* = 1.96 and intermolecular interaction *zJ* = −0.02 cm^–1^ (for **[VCuV]­[BF**_**4**_ **]**), respectively, which further confirms the ferromagnetic interaction between spin carriers.

In a previous study,^13^ we reported that the square-planar Ni­(II) bridge promotes antiferromagnetic interaction (*J* = −3.0 cm^–1^) between FeNO spin carriers. The strength of the coupling increases to *J* = −23 cm^–1^ and further to *J* = −123 cm^–1^ with replacement of the central Ni­(II) with Pd­(II) and Pt­(II), respectively. This leads to the diamagnetic spin ground state *S* = 0 at 2 K. In contrast, in the present studies, the tetrahedral Cu­(I) promotes ferromagnetic interactions (*J* = +3.5 cm^–1^) between the FeNO spin carriers. Investigation using theoretical tools revealed that this is due to the different d-orbital involvement of the central bridging metal, *vide infra.* Weak magnetic exchange coupling of similar magnitude (ca. ±5 cm^–1^) through diamagnetic bridging centers in trimetallic complexes has been documented in the literature, although the ligand backbone was different.^49–51^

### Mössbauer Spectroscopy

The Enemark-Feltham notation is commonly used to describe iron-nitrosyl species to avoid controversial oxidation state assignments in heavily delocalized metal–ligand systems.^52^ However, it cannot (and does not claim to) distinctively identify the actual ground state; a given {Fe-(NO)}^7^ can have an *S* = 1/2 ground state with low-lying excited states such as 3/2 or 5/2 being very close in energy.^53^ Given the EPR spectra *(vide infra)* display signals at *g* = 2 and *g* = 8 in almost equal ratio, it may be possible that a *g* = 8 signal is arising from ferromagnetic coupling between two {Fe-(NO)}^7^ centers having spin states of 3/2 and 1/2, giving an overall *S* = 2 species (Figure 4, quintet state). To estimate this possibility, we recorded zero-field ^57^Fe Mössbauer spectra of microcrystalline solids of **[FeCuFe]­[PF**_**6**_ **]** at 4.2 K (Figure S28). The Mössbauer data reveal a single quadrupole doublet with an isomer shift of 0.32 mm/s and a quadrupole splitting of 0.93 mm/s, slightly different from the free “metallo-ligand”, (bme-dame)­Fe­(NO),^14^ that exhibits an isomer shift (δ) of 0.27 mm s^–1^ and a quadrupole splitting (Δ*E*_Q_) of 1.14 mm s^–1^. The observed difference from the free “metallo-ligand” in Δ*E*_Q_ is attributed to changes in the environment at the Fe center, as the bidentate S-donors, shared with Cu­(I), result in a slight difference in coordination geometry and a redistribution of electron density as reflected by the changes in the ν­(NO) IR stretch. Notably, these values are practically identical to those measured for **[FeM′Fe]** ^**2+**^ congeners (M = Ni­(II), Pd­(II), Pt­(II)).^13^ Based on the above, we conclude that two {Fe­(NO)}^7^ units in **[FeCuFe]­[PF**_**6**_ **]** are identical and have a spin state of *S* = 1/2 on each Fe center.

**Figure 4:**
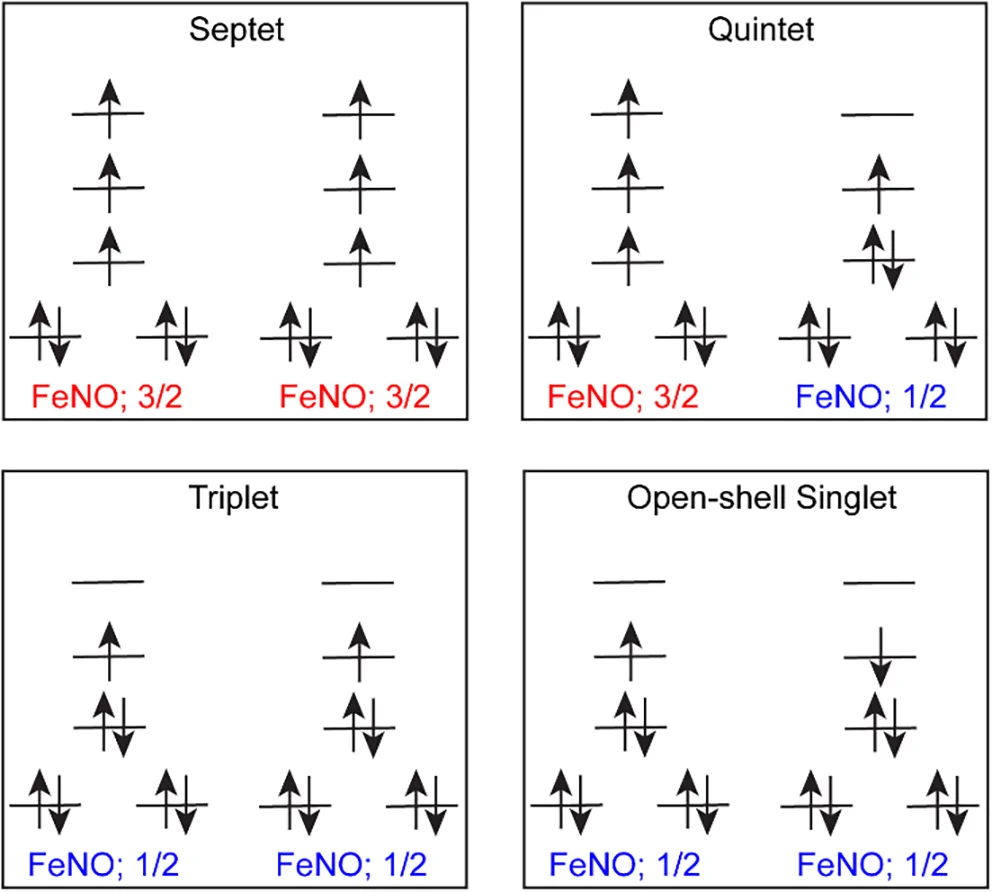
Examples of possible spin-coupling schemes for two [Fe­(NO)]^7^ units in the copper-bridged diiron complex.

### Quantum Chemical Calculations: Gas Phase

Further insight into electronic structure and magnetic behaviors of the trimetallic complexes derives from a series of computational calculations (for details, see the Supporting Information). The geometries of the complexes **[FeCuFe]** ^**+**^ and **[VCuV]** ^**+**^ were optimized in the gas phase without any constraints and found to retain the structural motifs as determined by the SC-XRD, including agreement for bond distances and angles despite the lack of crystal packing and intermolecular interactions in the calculations (Figure S29). Due to the large separation (∼6 Å) between two spin probes (FeNO and VO) in both complexes, direct spin interaction between them is unlikely. Thus, through-bond magnetic exchange mediated by bridging sulfur atoms connecting M (VO/FeNO) and M′(Cu) separated at 2.9 Å was expected. Analysis of these interactions requires appropriate orbital orientation and symmetry.

Most descriptions of the electronic structure of the Fe­(NO) unit are based on an assumed oxidation state of iron as it exists in the particular ligand field and its interaction with NO π* electrons.^53^ For [Fe­(NO)]^2+^ as paramagnetic {Fe­(NO)}^7^ (E-F notation), two primary spin-state possibilities (*S* = 1/2 and *S* = 3/2) are expected depending on low-spin or high-spin configuration of the iron center. The presence of two {Fe­(NO)}^7^ centers in the **[FeCuFe]** ^**+**^ complex gives rise to multiple spin-coupling possibilities, some of which are depicted in Figure 4. Accordingly, geometry optimizations were carried out for the open-shell singlet (broken-symmetry), triplet, quintet, and septet spin states; a solvent correction (acetonitrile) was used in the energy calculation. As anticipated, the open-shell singlet exhibits significant spin contamination (⟨*S* ^2^⟩ ≈ 1.3), while the triplet excited state shows minimal spin contamination (⟨*S* ^2^⟩ ≈ 2.3) except for B3LYP (Table S4). For all the density functionals examined, open-shell singlet and triplet states are nearly isoenergetic, while the quintet and septet spin states are higher in energy, again, excepting B3LYP (Table S4). Considering that the experimentally observed solution- and solid-state magnetic data support a low-temperature ground state with ferromagnetic interactions of two unpaired electrons for the **[FeCuFe]** ^**+**^ system, higher excited spin states were considered unlikely to be populated. Complex **[VCuV]** ^**+**^ was optimized in open-shell singlet and triplet spin states, resulting in similar energies for both states (Table S5) and an interpretation paralleling that for the **[FeCuFe]** ^**+**^ system.

The upper portion of Figure 5 shows the three highest-energy, occupied α- and β-MOs of the broken-symmetry (BS) singlet state, while the corresponding triplet orbitals are given in Figure S22. The BS solution can be stabilized by the overlap of the α and β orbitals, providing a valence-bond description of the antiferromagnetic interaction.

**Figure 5:**
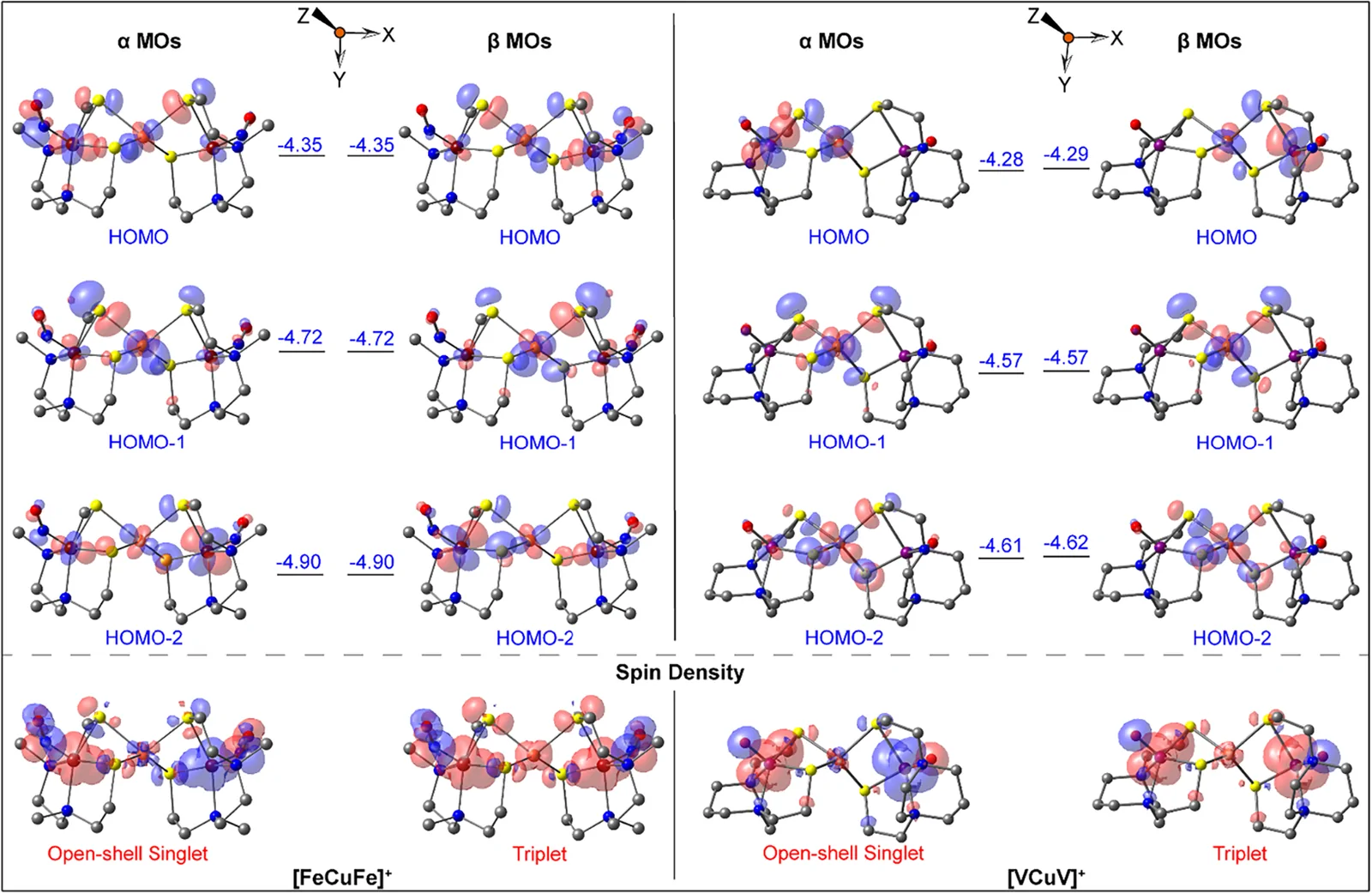
(Top) Frontier molecular orbitals of **[FeCuFe]** ^**+**^ and **[VCuV]** ^**+**^ (open-shell singlet). The MO surfaces are plotted at an isosurface value of 0.05 au, and energies are in eV. (Bottom) Spin density plots (isovalue = 0.002).

In **[FeCuFe]** ^**+**^, these HOMOs originate from FeNO-centered orbitals with significant contribution from the coordinating sulfur and smaller contributions from the bridging copper atoms. The copper bridge provides an electronic communication pathway between the two FeNO units. As expected, α- and β-HOMOs appear as near mirror images of each other. However, the Cu contributions to the α- and β-HOMOs are nearly orthogonal, which limits their mixing and reduces the stabilization of the singlet. Overall, there is insufficient overlap between the α- and β-manifolds to stabilize the singlet state, such that the triplet state is the ground state. Similar observations can be found in the case of **[VCuV]** ^**+**^, where the spatial distribution of copper in both the α- and β-HOMOs spin manifolds provides moderate mixing, resulting in closely spaced singlet and triplet states. Overall, in **[FeCuFe]** ^**+**^, HOMOs are more delocalized over the dithiolate ligand than in the **[VCuV]** ^**+**^ case.

It is helpful to compare the spin density plots, lower portion of Figures 5 and S25, that are fundamental to the observed magnetic behavior. The spin density distribution suggests that in the **[FeCuFe]** ^**+**^ complex, most of the spin density is localized on the FeNO units, with minor delocalization onto the sulfur donors and the copper center. Likewise, in **[VCuV]** ^**+**^, most of the spin density is on the vanadium with even less delocalization onto the Cu and S. This indicates that less spin polarization is through the ligand framework and accounts for the smaller magnitude of magnetic coupling as experimentally observed.

In summary, molecular orbital analysis (Figures 5, S22, and S24) of FeNO suggests that its unpaired electron resides in a Fe d orbital which does not overlap well with the Cu­(I) orbitals in the tetrahedral CuS_4_ bridging unit. However, in group 10-bridged **FeM′Fe** complexes, the Fe and M′ orbitals are well aligned, accounting for the relatively strong antiferromagnetic coupling. The V­(IV)≡O spin probe has its unpaired electron in the *d*_*xy*_ orbital, which couples poorly through the Cu unit. However, its coupling is even weaker in the group 10, **VM′V** (M = Ni, Pd, Pt), complexes.

### Electrochemistry

Solution electrochemical data of **[FeCuFe]­[PF**_**6**_ **]** recorded in CH_3_CN solvent reveals two reversible responses in the cathodic direction from the open circuit potential (Figure 6B), similarly to the cyclic voltammograms recorded for the isolated dicationic stair-step trimetallics, **[FeM′Fe]** ^**2+**^, where M = Ni^2+^, Pd^2+^, and Pt^2+^.^13^ The most accessible, at −1.03 V for the Cu^I^ series, is reasonably assigned to a neutral, mixed-valent {Fe­(NO)}^7^–Cu^I^–{Fe­(NO)}^8^ complex. Should the added electron be spread over both {Fe­(NO)} centers, the diiron species might be represented collectively as {CuFe_2_(NO)_2_}^15^. That is, equal apportionment of charge from the added electron through bond allocation might yield in the Enemark-Feltham notation, {Fe­(NO)}^7.5^. Such ambiguity as we have detected earlier in the diiron trinitrosyl, {Fe­(NO)}^7^–{Fe­(NO)_2_}^10^, system requires further investigation by experiment and theory and will be discussed later.^16,54^

**Figure 6:**
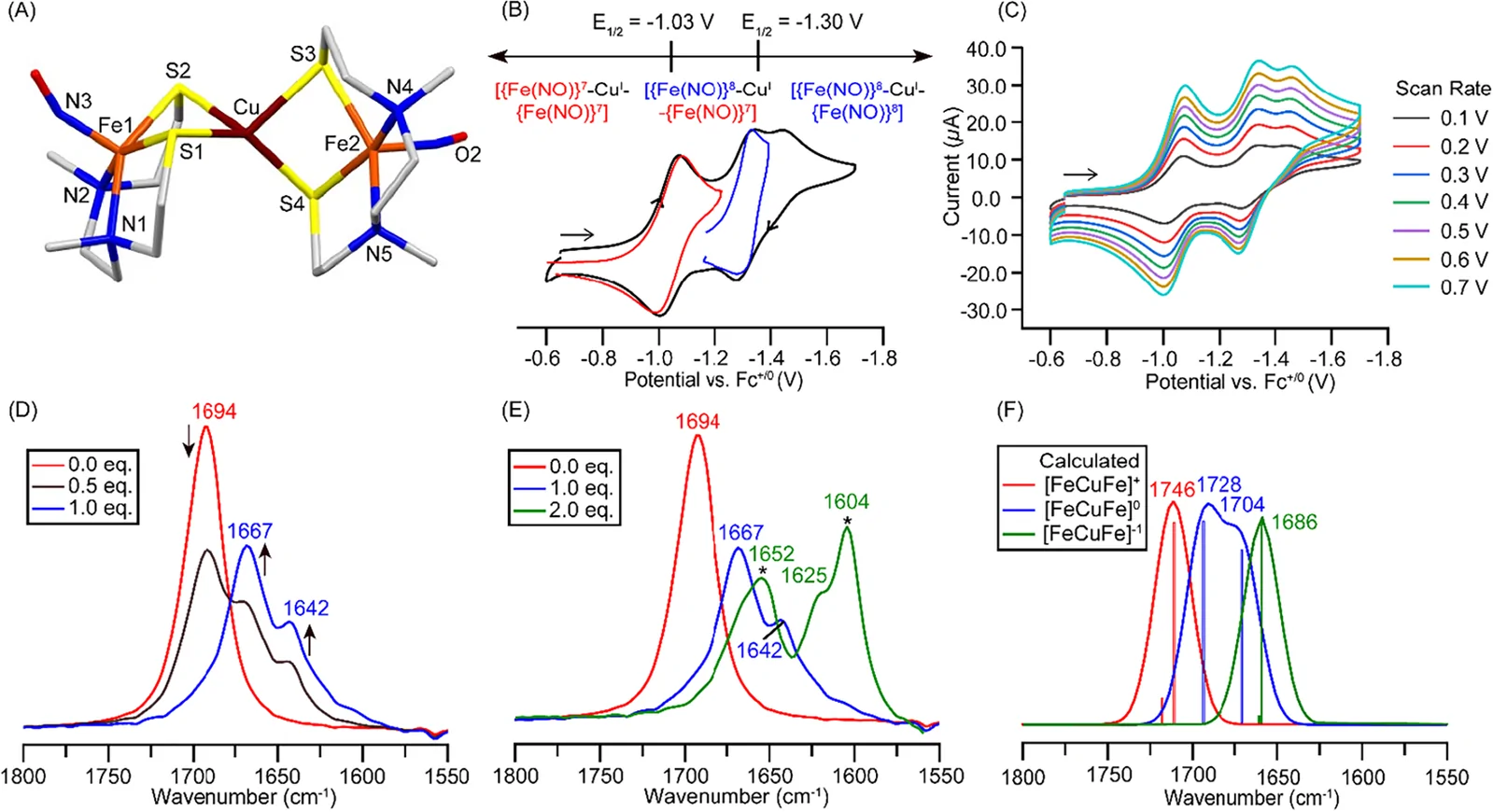
(A) Molecular structure (at 100 K) of **[FeCuFe]** ^**+**^ showing labeling of atoms. (B) A portion of cyclic voltammogram (in CH_3_CN at 295 K) of **[FeCuFe]­[PF**_**6**_ **]** (scan rate 100 mV/s) with 0.1 M (*n*-Bu)_4_N^+^PF_6_^–^ as supporting electrolyte (selected range showing reversibility for first and second reduction are given in red and blue). CV plotted as per polarographic (US) convention. The reference electrode was Ag^0^/AgNO_3_. (C) Scan rate dependence plots for the reversible redox couple. (D) IR spectral changes observed (in CH_3_CN at 295 K) during the reaction of a 10 mM solution of **[FeCuFe]­[PF**_**6**_ **]** with 0–1.0 equiv of 1 M (THF) KHBEt_3_ (red: initial **[FeCuFe]­[PF**_**6**_ **]** complex; blue: 1e^–^ oxidized product). (E) IR spectra (in CH_3_CN at 295 K of 10 mM solutions) comparing **[FeCuFe]­[PF**_**6**_ **]** (red), after 1-electron reduction (blue), and 2-electron reduction (green). (F) Comparison of calculated IR spectra (calculation values from unscaled models).

At the second response (−1.30 V), the expectation is that both [Fe­(NO)]^7^ units take up an electron, leading to the formation of {Fe­(NO)}^8^–Cu^I^–{Fe­(NO)}^8^ or {Fe_2_(NO)_2_}^16^. This assignment is consistent with the previously reported one redox event in the parent (bme-dame)­Fe­(NO) ligand^39^ and the two redox events in group 10-based **[FeM′Fe]** ^**2+**^ congeners,^13^ arising at similar potentials. The linear dependence of current with respect to the square root of scan rate suggests that the two events in both families are fully reversible and governed by diffusion (Figure 6C). Additional measurements focused on the selected potential regions of the first and second reductions further confirmed their reversible behavior; however, the third reductive event overlaps with the second in the Cu­(I) family of trimetallics and was irreversible and remains at this time unassigned. A comparison of the separation between successive redox processes (Δ*E*), provides insight into potential electronic communication between the two Fe­(NO) centers.^55^ In the case of group 10-based **[FeM′Fe]** ^**2+**^ complexes, the difference in *E* values was 0.21 V for M = Ni^II^ and Pt^II^, and 0.25 V for M = Pd^II^. The greater value found for **[FeCuFe]­[PF**_**6**_ **]** of 0.30 V suggests increased delocalization in the copper complex, however the charge difference between the reduced product species (monocation for the **[FeM′Fe]** ^**2+**^ and neutral for the **[FeCuFe]** ^**+**^ species) limits the ability to make a straightforward comparison, given prior reports demonstrating that shifts in redox potential are predominantly governed by electrostatic effects due to the cationic charge.^56,57^ In addition to the two reversible redox events in the **[FeCuFe]** ^**+**^ system, an irreversible event is observed at approximately −1.35 V. Possibilities for the origin of this event include reduction of Cu­(I) to Cu(0) or from a reconfiguration and the formation of {Fe­(NO)}^9^ species, both of which, especially the latter, are rare, and expected to be unstable. While definitive assignment is not possible, this cathodic process appears to generate a small redox feature at −0.50 V in the reverse scan of the full-range cyclic voltammogram (Figure S26). Such behavior could plausibly arise from the reoxidation of a surface-adsorbed Cu(0) species formed during the Cu­(I)/Cu(0) reduction, in line with previous reports.^58^ However, this feature vanished after rinsing the working electrode, indicating the absence of a strongly bound surface species. That is, if Cu(0) was formed, it did not deposit on the electrode. Described below are attempts to chemically “over-reduce” the **[FeCuFe]** ^**+**^ trimetallic with results that discourage speculation on the specific origin of this reduction event.

#### Interrogation of the Electrochemical Assignments/Reduction Products by Vibrational Spectroscopy

While isolation and full characterization of the products from chemical reductions of **[FeCuFe]­[PF**_**6**_ **]** have not yet been accomplished (as was done for the **[Fe**_**2**_ **(NO)**_**3**_ **]** ^**+/0/–**^ system),^16^ we pursued the ν­(NO) vibrational marker as an *in situ* tool for monitoring changes at the {Fe­(NO)} centers with successive reductions. Attempts were therefore made to characterize the one- and two-electron reduced forms of **[FeCuFe]­[PF**_**6**_ **]** via bulk chemical reduction using cobaltocene (Cp_2_Co, *E*_1_/_2_ = −1.33 V) or the moderately powerful reducing agent, KEt_3_BH.^59^ Figure 6D,E displays the changes in the ν­(NO) infrared region of **[FeCuFe]­[PF**_**6**_ **]** as equivalents of KEt_3_BH were added to acetonitrile solutions. Specifically, the intensity of the single ν­(NO) IR band at 1694 cm^–1^ in acetonitrile is seen to decrease upon gradual addition of KEt_3_BH, up to 1.0 equiv, concomitant with the appearance of two new bands at 1667 cm^–1^ and 1642 cm^–1^ that grow at the same rate (Figure 6D). After completion of the reaction (monitored by a color change from brown to violet), the band at 1694 cm^–1^ completely disappeared. The shift to lower wavenumbers is attributed to the increase in electron density in the antibonding orbital of NO. Note that both {Fe­(NO)} centers experience the added electron or negative charge, and the complex, in the one-electron reduced state, can be assigned, according to E-F notation, to {Fe_2_(NO)_2_}^15^. Upon further addition of KEt_3_BH, the intensity of the absorptions at 1667 cm^–1^ and 1642 cm^–1^ decreased and completely vanished after addition of 2.0 equiv of reductant (Figure S27A). During this process, three new bands at 1652 cm^–1^, 1625 cm^–1^, and 1604 cm^–1^ appeared; these changes are accompanied by a color change from violet to olive-green. Further additions up to 3.5 equiv of reductant increase the intensity of the absorption at 1625 cm^–1^ and 1604 cm^–1^, finally decreasing upon further addition of reductant. After addition of 4.0 equiv of reductant, the intensity of bands at 1652 cm^–1^, 1625 cm^–1^, and 1604 cm^–1^ decreased concomitantly with the appearance of new absorption at 1675 cm^–1^.

Cobaltocene was also employed as a reductant, as its redox potential (*E*_1/2_ = −1.33 V vs. Fc/Fc^+^) closely matches the second reduction potential of the complex. We therefore anticipated that controlled reduction might be achieved without inducing over-reduction. Addition of up to 1.0 equiv CoCp_2_ resulted in the same spectral pattern as KEt_3_BH, featuring two absorption bands at 1667 cm^–1^ and 1642 cm^–1^ (Figure S27B). Upon further addition of 1.5 equiv of cobaltocene, the band at 1642 cm^–1^ completely vanished and the intensity of absorption at 1667 cm^–1^ decreased along with the appearance of a new band at 1625 cm^–1^, which we assign to the two-electron-reduced complex. Further addition of cobaltocene resulted in over-reduction, yielding spectral features (1675 cm^–1^, 1652 cm^–1^, and 1604 cm^–1^) identical to those obtained upon reduction with KEt_3_BH.

This latter band, presumed to be from over-reduction, is tentatively assigned to a degradation product known for the metallodithiolate ligand, (N_2_S_2_)­Fe­(NO), and the formation of a member of the [(N_2_S_2_)­Fe­(NO)-Fe­(NO)_2_]^+/0/–^ redox family. This assignment has inspired further mechanistic studies of NO/Fe/S reconfiguration to be reported in a separate paper.^16^

#### Analysis of the Electrochemical Responses of the Vanadyl Trimetallic Complex Cations

Unlike **[FeCuFe]­[PF**_**6**_ **]**, the **[VCuV]­[PF**_**6**_ **]** shows complex redox behavior and no reversible events (Figures S28 and S29). There is an event at 0.39 V which can be attributed to oxidation of the thiolate S atoms, similar to what is observed in the parent complex (bme-dach)­V­(O).^60^ An additional oxidative event at +1.34 V may be assigned to an irreversible Cu­(I) to Cu­(II) oxidation. In the cathodic sweep, an event is observed at −2.00 V, which is more positive than the potential for the “free ligand” vanadyl component, (bme-dach)­V­(O). This may be due to the electron-withdrawing effect of the central Cu in the MS_4_ bridge. On return sweeps, reductions are observed at 0.62 V and −1.79 V, tentatively assigned as Cu­(II) to Cu­(I) and Cu­(I) to Cu(0) reductions in one of the oxidized byproducts. However, none of these possibilities were confirmed or further examined.

#### Assignments of Vibrational Modes by DFT Calculations

Various levels of theoretical calculations were carried out in attempts to assign the experimentally observed IR spectra of **[FeCuFe]** ^**+/0/–1**^ (Figure 6F and Table S6). For the cationic species, two closely spaced ν­(NO) bands resulted: an intense asymmetric stretch at 1746 cm^–1^ and a very weak symmetric stretch at 1753 cm^–1^. The extremely low intensity of the latter explains why only a single IR band is experimentally observed, and is consistent with very small coupling of the two centers. (N.B., the unscaled calculated values differ from the experimental results, commonly expected for computational methods; however, the relative wavenumber differences between the cationic, neutral, and anionic species are clearly maintained). Upon one-electron reduction, the ν­(NO) band shifts to lower wavenumbers and splits into two components of almost equal intensity, giving an asymmetric stretch at 1704 cm^–1^ and a symmetric stretch at 1728 cm^–1^. Further reduction results in an additional shift, yielding once more a band at 1686 cm^–1^ (asym) and a weak symmetric band at 1689 cm^–1^ (sym).

Note that the change in ν­(NO) IR bands to the addition of electrons indicates that the extra electron is slightly delocalized rather than restricted to a single site. This behavior is consistent with a Robin–Day Class II mixed-valence system.^61^ Notably, a change in color from brown (cationic complex) to violet (neutral complex) to green (anionic complex) is observed upon reduction; this visual effect is accompanied by a disappearance of the 485 nm band in the UV–vis spectra recorded in MeCN at −30 °C with concomitant appearance of two new bands at 550 nm and a characteristic intervalence charge transfer (IVCT) broad band at 675 nm upon addition of 1.0 equiv reductant (Figure S30). Further addition up to 2.0 equiv. reductant results in the disappearance of the 550 nm band and the growth of a new absorption at 560 nm, while the band at 675 nm becomes more pronounced. Emergence of the band at 675 nm further supports the delocalization of charge involving the FeNO centers through the copper bridge.^62^ TDDFT calculations reproduce the low-energy band at 675 nm characteristic of an IVCT transition, arising primarily from β­(HOMO–3) → β­(LUMO) and α­(HOMO–1) → α­(LUMO+1) excitations involving both FeNO units (Figure S31). The β­(HOMO–3) is localized on one FeNO center while the β­(LUMO) resides on the other, consistent with intersite electron transfer and is, again, indicative of a Robin–Day Class II mixed-valent system.

This conclusion is further supported by the computed charge and spin distribution (Table 2). In the complex **[FeCuFe]** ^**+**^, each iron center carries a charge of 0.56 and a spin of 1.53. After one-electron reduction, one Fe center shows a charge of 0.55 and a spin of 1.39, while the second adopts 0.62 and 3.01. Likewise, both NO ligands have similar charge/spin values in the neutral state (−0.32/–0.41) but become differentiated upon one electron reduction (−0.34/–0.40 and −0.54/1.20). Small systematic variations in Cu and S charges/spins are also observed. These collective changes reinforce the presence of slight charge and spin delocalization. Further, the second reduction generates **[FeCuFe]** ^**–1**^, having both iron centers carrying the same charge and spins.

**Table 2:** Mulliken Charge and Spin Population Calculated from UTPSSH/6-311+G**/def2TZVP Optimized Geometries of the High-Spin State of **[FeCuFe]** ^**+**^, **[FeCuFe]** ^**0**^, and **[FeCuFe]** ^**–1**^

	**[FeCuFe]** ^**+**^		**[FeCuFe]** ^**0**^		**[FeCuFe]** ^**–1**^	
	Charge	Spin	Charge	Spin	Charge	Spin
**Fe1**	0.56	1.43	0.62	3.01	0.60	3.00
**Fe2**	0.56	1.43	0.55	1.39	0.60	3.00
**(NO)1**	–0.32	–0.41	–0.54	–1.20	–0.54	–1.21
**(NO)2**	–0.32	–0.41	–0.34	–0.40	–0.54	–1.21
**Cu**	0.17	0.02	0.20	0.02	0.23	0.00
**S1**	–0.28	–0.01	–0.49	0.06	–0.52	0.07
**S2**	–0.29	0.01	–0.50	0.09	–0.52	0.07
**S3**	–0.29	0.01	–0.30	0.02	–0.52	0.07
**S4**	–0.28	–0.01	–0.30	–0.01	–0.52	0.07

Analysis of the frontier molecular orbitals reveals significant electronic delocalization that underpins the complicated redox chemistry of these complexes. In **[FeCuFe]** ^**+**^, HOMO and HOMO–1, having small energy differences, primarily originate from FeNO-centered orbitals with significant contribution from the coordinating sulfur and the bridging copper atoms. HOMO and HOMO–1 span both FeNO centers, suggesting delocalization of electron density. While oxidation processes have not been examined in this study, unstable species are expected to be triggered by the instability of Cu­(II) in the current complex. In contrast, reduction, using the LUMO and LUMO+1 as a guide in the **[FeCuFe]** ^**+**^, suggests that sequential insertion of electrons would proceed into FeNO-based orbitals along with minor contributions from sulfur-based orbitals (Figures 5 and S18). LUMO and LUMO+1 are close in energy (Δ*E* = 0.04 eV), confined on both FeNO centers, suggesting that an added electron would delocalize over both FeNO sites. In fact, inspection of frontier molecular orbitals of one- and two-electron-reduced **[FeCuFe]** ^**0**^ and **[FeCuFe]** ^**–1**^ revealed that the energetically close HOMO to HOMO–3, located on both FeNO sites, suggests delocalization of the added electron (Figure S19). These results account for the convoluted features observed in the cyclic voltammograms, the synthetic challenges associated with isolating discrete oxidized and reduced species, and the confusing IR signature of the NO stretching modes in the one-electron reduced species (*vide infra*).

### EPR Studies in Solution

EPR spectroscopy complements the magnetometry in that while the latter is a view of overall magnetism as affected by temperature, the former gives a snapshot of spin location and its surroundings in the molecular structure at a particular temperature. Figure 7 compares the X-band EPR spectra collected for frozen samples (10 mM) of **[FeCuFe]­[PF**_**6**_ **]** and **[VCuV]­[PF**_**6**_ **]** dissolved in acetonitrile. In transverse mode (⊥), both complexes exhibit isotropic signals with minor shifts relative to the free electron *g*-value (2.0023). Samples of **[FeCuFe]­[PF**_**6**_ **]** exhibit an observed *g*-value of 2.03 with a sharp line width (full width at half-maximum (FWHM) of 10 mT). By contrast, the observed isotropic *g*-value decreases to 1.97 for samples of **[VCuV]­[PF**_**6**_ **]**, along with a >4-fold increase in line width (FWHM = 42 mT) relative to **[FeCuFe]­[PF**_**6**_ **]**. This increase in line width can be attributed to unresolved hyperfine originating from the two ^51^V (*I* = 7/2) nuclei.

**Figure 7:**
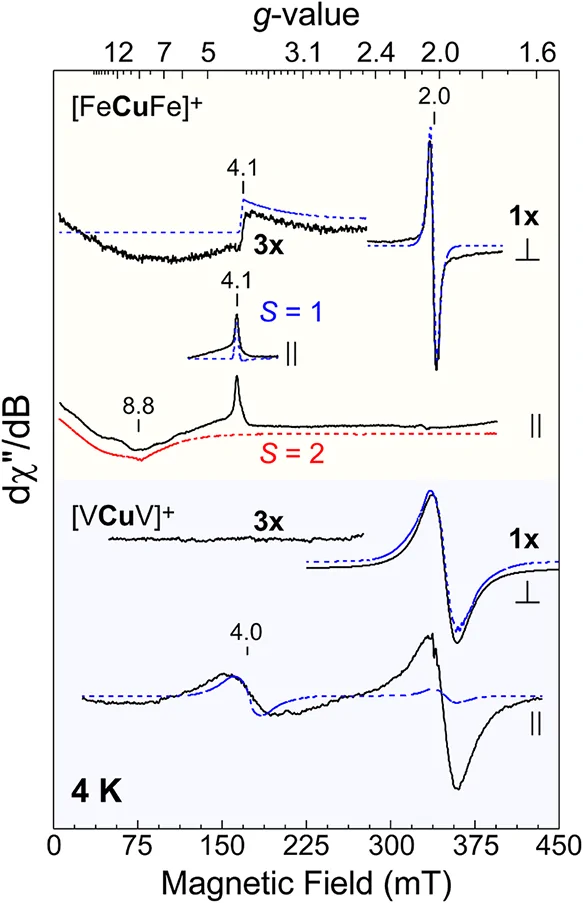
Comparison of perpendicular and parallel mode X-band EPR spectra collected for **[FeCuFe]­[PF**_**6**_ **]** (*top*) and **[VCuV]­[PF**_**6**_ **]** (*bottom*). Analytical EPR simulations (*dashed lines*) performed on both microwave field polarizations are overlaid on experimental results (*solid line*) for comparison. Instrumental settings: microwave frequency, 9.64 GHz ⊥, 9.41 GHz ∥; microwave power, 0.3 mW ⊥, 3.0 mW ∥; modulation amplitude, 0.9 mT; temperature, 4 K. Simulation parameters provided in Table S7.

The intensity of integer-spin transitions is influenced by a variety of factors that introduce mixing of eigenstates.^63^ Since the *m*_s_ values are no longer in the good quantum number regime, the EPR selection rule (D*m*_s_ = ±1) is no longer strictly valid. These “forbidden” transitions can frequently be observed by parallel mode EPR with an observed *g*-value ∼4 × D*m*_s_. For example, an observed *g*-value of 4 would be expected for a transition between the |-1> and |+1> *m*_s_-doublet (D*m*_s_ = 1) of a triplet (*S* = 1) spin system. Consistent with this logic, both **[FeCuFe]­[PF**_**6**_ **]** and **[VCuV]­[PF**_**6**_ **]** samples exhibit transitions at *g* ∼ 4.0 at 4 K. As with the features observed in perpendicular mode, the *g* ∼ 4 resonance observed for samples of **[VCuV]­[PF**_**6**_ **]** exhibits significantly greater line width relative to samples of **[FeCuFe]­[PF**_**6**_ **]**. At elevated temperature, the signal intensity for these transitions in both complexes decreases beyond what can be accounted for by Curie law, suggesting depopulation of a ground |±1> doublet and ferromagnetic coupling. EPR simulations for both complexes (Figure 7, *blue dashed lines*) faithfully reproduce the spectroscopic features observed in both perpendicular and parallel modes. A summary of the spectroscopic parameters used for these simulations is provided in Table S7.

An additional broad feature with a valley centered at *g* ∼ 8.8 is observed in samples of **[FeCuFe]­[PF**_**6**_ **]**, suggesting a transition within a higher D*m*_s_-doublet. EPR simulations for a triplet spin cannot account for this additional low-field feature; however, a simulation for a transition between a |±2> doublet nicely reproduces the low-field trough observed at *g* ∼ 8.8. Similar parallel mode EPR features have been reported previously for *S* = 2 paramagnetic centers.^64–66^

When considering the placement of the {Fe­(NO)}^7^ units at the opposing ends of the **[FeCuFe]­[PF**_**6**_ **]** molecule, it is possible that additional spin couplings are introduced from intermolecular coupling. In fact, close inspection of the solid-state structure of our complex (Figure 2c) reveals a Heisenberg chain-like structure where each NO group on one **[FeCuFe]­[PF**_**6**_ **]** molecule interacts with flanking NO groups on molecules to either side (N_NO_-O_NO_ distances are 3.21 Å and 3.63 Å). As the intermolecular Fe-NO···NO distance (3.2 Å) is nearly half the intramolecular Fe–Fe distance (5.7 Å), formation of higher (*S* = 2) spin-manifolds occurs. In [FeCuFe]­[PF_6_], the intramolecular coupling between the {Fe­(NO)}^7^ spin centers is ferromagnetic and dominates the magnetic behavior, as reflected in the increase of χ_*M*_ *T* at low temperature (*vide supra*). The intermolecular interactions, although associated with a shorter Fe–NO···NO contact (3.2 Å), arise primarily from through-space (dipolar) interactions, as there is no significant orbital overlap between the interacting units. As a result, these intermolecular interactions are weak and antiferromagnetic in nature, leading to the slight decrease in χ*_M_T* at very low temperatures. Therefore, despite the shorter intermolecular distance, the intramolecular exchange remains stronger.

This reasoning is strengthened by the *S* = 2 simulation (*red dashed line*) shown in Figure 7. The possible existence of such intermolecular interactions in solution (at 4 K) provides a reasonable explanation for the observation of transitions within |±2> and |±1> doublets at *g* ∼ 8.8 and 4.1, respectively.

The EPR data for the **[VCuV]­[PF**_**6**_ **]**complex are shown for comparison. Significantly, no low-field transitions are observed to suggest spin manifolds greater than *S* = 1. The simulated spectrum represented by a dashed blue line assumes only a triplet spin state. Within the **[VCuV]­[PF**_**6**_ **]** complex, it is expected that the greater localization of unpaired spin density on the V-site, along with the longer V≡O···O=V distance, contributes to the absence of low-field parallel mode EPR features associated with intramolecular coupling.

## Conclusion

The structural and spectroscopic properties of heterobimetallic complexes in which the metals are bridged by thiolate sulfur have highlighted the ability of sulfur to influence geometry through lone-pair-guided hinge angles in M­(μ-SR)_2_M′.^14,16^ Further, the electron-delocalizing, electron-buffering ability of sulfur leads to stabilization of multiple redox levels similar to π-delocalization in unsaturated organic polypyridyl ligands. Few examples exist that extend such characterization to (μ-SR) bridged trimetallics, particularly in addressing the possibility of communication between distal metals in [M­(μ-SR)_2_·M′·(μ-SR)_2_M] arrangements.

By use of the N_2_S_2_ ligand framework in trimetallic coordination complexes, we have established magnetic exchange and electronic interactions via [Fe­(NO)]^2+^ vs [VO]^2+^, *S* = 1/2, spin probes, allowing comparison of electronic coupling through geometrically distinct bridging metals, square planar^13^ versus tetrahedral (this work), within the same donor ligand environment. The redox-active nature of the nitrosyl ligand enables access to multiple electronic descriptions, including NO^–^, NO^•^, and NO^+^ character, which can be effectively monitored through the ν­(NO) vibrational marker. Infrared spectroscopy suggests that the central metal plays a critical role in mediating charge redistribution in the reduced {Fe­(NO)}^7^–M′–{Fe­(NO)}^8^ system. In the case of copper, it generates a Robin–Day class II mixed-valence species (Table 2). Such distributions of charge and spin *loci* determined by vibrational analysis and computations are linchpins for the interpretation of other experimental results, suggesting that such trimetallics may represent fundamental units of use to the interpretation of electronic and magnetically conducting extended structures.

Comparison of [Fe–NO] and [V≡O] spin probes highlights the fundamental differences in electronic structure. Although [Fe­(NO)]^2+^ and [VO]^2+^ units can be considered formally isoelectronic in terms of spin count, their frontier molecular orbital descriptions are far from being identical. (Scheme 1, Figure S20). In [Fe­(NO)]^2+^, the unpaired spin has predominant d_*z*_^2^ character and is delocalized over the adjacent sulfur atoms. However, the [VO]^2+^ spin is located in the d_*xy*_ orbital, which is localized at the relatively electropositive V­(IV). Such a kind of electron localization is preferred in quantum computing devices as it offers longer spin–spin relaxation time, a critical factor to determine how long information can be stored in a spin orientation.^67–69^

Temperature-dependent magnetic measurements offer promise for establishing the existence of well-separated communication centers of spin-labeled metals. Results from magnetometry (SQUID) for the compilation of S-bridged MS_2_M′S_2_M trimetallic compounds, measured on the resting state redox levels in this report, are compared in Figure 8.

**Figure 8:**
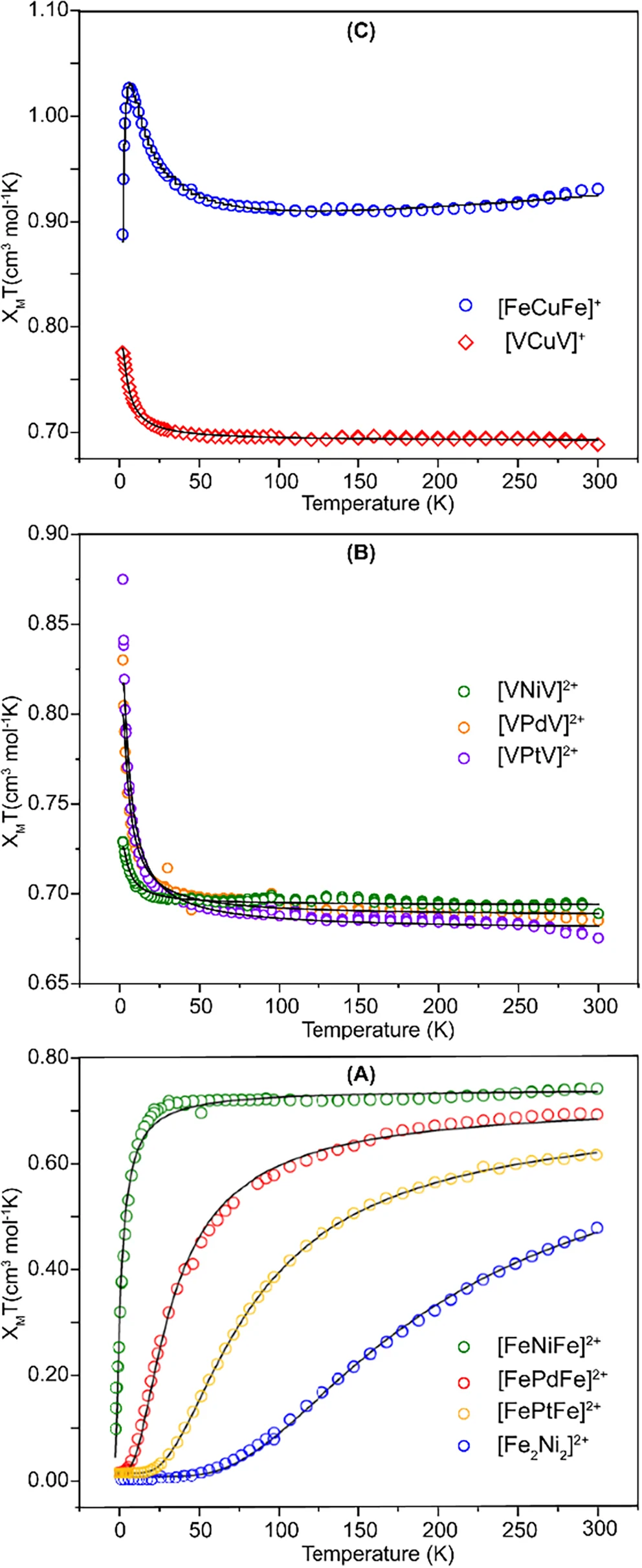
Comparison of temperature-dependent magnetic susceptibilities for the previously reported metal complexes (data sets **A** and **B**, adapted from references 12 and 15) with the metal complexes in this study (data set **C**).

For all compounds except **[FePtFe]** ^**2+**^ and **[FeCuFe]** ^**+**^, the room-temperature χ_M_T values are close to 0.75 cm^3^ K mol^–1^, consistent with the expected value for two uncoupled *S* = 1/2 centers (*g* = 2) at 300 K. In contrast, **[FePtFe]** ^**2+**^ (Data set **A**)^13^ exhibits a significantly lower χ_M_T value (0.49 cm^3^ K mol^–1^), indicative of contributions from strong antiferromagnetic coupling. Data set **C** (this work) for **[FeCuFe]** ^**+**^ displays a χ_M_T value (∼0.90 cm^3^ K mol^–1^) and coupling constant *J* 3.5 cm^–1^. These results demonstrate that the bridging M′ cation plays a decisive role in controlling magnetic exchange, leading to a switch from antiferromagnetic to ferromagnetic coupling upon replacing group 10 metals (Ni^2+^, Pd^2+^, Pt^2+^), having square-planar geometry, with tetrahedral Cu­(I) in the M′S_4_ bridge.

Data set **B**,^15^ for which the distal spin centers relate to the *S* = 1/2 [V≡O]^2+^ in the MN_2_S_2_ metallodithiolate ligand that templates the trimetallic, shows a switch to weak ferromagnetic coupling when connected through square-planar MS_4_ centers. A presentation of these results in tabular form is given in Table 3.

**Table 3:** Summary of Magnetic Results for Sulfur-Bridged M-M′-M Cations^a^

M	M′	M···M (Å)	Magnetism at R.T. (BM)	Coupling at 4 K (J, cm^–1^)	ref
[Fe(NO)]^2+^ *S* = 1/2 [Fe(NO)]^7+^	Ni^2+^	5.911	2.47	–3.00	^13^
	Pd^2+^	6.013	2.37	–23.00	^13^
	Pt^2+^	6.017	1.98	–124.53	^13^
	Cu^+^	5.711	2.47	+3.13	This
**[V≡O]** *S* = 1/2**, d**_***xy***_^**1**^	Ni^2+^	5.979	2.35	+0.28	^15^
	Pd^2+^	6.272	2.34	+0.95	^15^
	Pt^2+^	6.228	2.32	+1.37	^15^
	Cu^+^	5.921	2.35	+1.63	This

a Magnetic moment at room temperature (R.T) taken from magnetometry. Magnetic data for [FeNiFe] and [VNiV] are taken from refs 13 and 15.

Copper-containing systems exhibit distinct electronic behavior, where the orthogonal arrangement of α and β spin density contributions at the Cu­(I) center stabilizes a triplet ground state (Figure 5) and disfavors singlet coupling, opposite to the group 10-bridged system. In addition, the tetrahedral geometry imposed by Cu­(I) introduces twisting that orients the FeNO or VO spin probes at different angles and also reduces effective magnetic exchange through the sulfur donor orbitals.

The evidence from variable-temperature magnetic susceptibility and EPR spectroscopic analyses suggests that magnetic behavior is further influenced by intermolecular spin–spin interactions in the copper system, particularly in the solid state, shown to have intermolecular radical–radical interactions between nitric oxide ligands from two distinct units, creating 1-dimensional chains. Extended packing diagrams in [**FeCuFe]­[PF**_**6**_ **]** crystals reveal double columns of cations in which NO ligands from the individual [FeCuFe]^+^ units interact both within the chain and between chains. Such interactions account for an *S* = 2 signal in the EPR spectra at 4 K.

The intricacies of the electronic structures of metallodithiolate ligands, first realized by Solomon et al.,^70^ portend a fertile field for development of molecular species of wide-ranging possibilities. The in-depth understanding of coordination and physical–inorganic chemistry fuels advancement in this area.

## Experimental Section

### Methods and Materials

All reactions and manipulations were performed either under inert atmosphere in a N_2_ glovebox or using standard Schlenk-line and syringe/rubber septa techniques under N_2_ atmosphere. Dry solvents were purified and degassed via a Bruker solvent system. Reagents were purchased from commercial sources and used as received. The known compounds: (L1 = *N,N*′-bis­(2-mercaptoethyl) diazamethylethane = (bme-dame)),^71^ (L2 = *N,N*′-bis­(2- mercaptoethyl)-1,5-diazacycloheptane = (bme-dach)),^38^ [(bme-dame)­Fe]_2_,^71^ VO­(bme-dach),^40^ Fe­(NO)­dame^45^ were synthesized following the literature reported procedures.

### Synthesis of [(NO)­Fe­(bme-dame)-Cu^I^-(bme-dame)­Fe­(NO)]­[PF_6_], [FeCuFe]­[PF_6_]

In a 20 mL vial, 50 mg (0.171 mmol) of (NO)­Fe­(bme-dame) was dissolved in approximately 8 mL of CH_3_CN. A solution of 31 mg (0.5 equiv, 0.085 mmol) of [Cu­(CH_3_CN)_4_]­PF_6_ dissolved in 5 mL of CH_3_CN was added to the solution of (NO)­Fe­(bme-dame). An immediate color change from dark green to brown was observed. The reaction mixture was stirred for another 30 min. The solution was concentrated, and excess Et_2_O was added to precipitate the product. The solid was redissolved in CH_3_CN and filtered through Celite. Dark brown crystals of the product were grown using vapor diffusion of Et_2_O into a solution of CH_3_CN at room temperature to yield 61 mg of product (90% yield). FTIR (CaF_2_ windows, CH_3_CN, 23 °C): ν­(NO) = 1694 cm^–1^ (s). UV–vis absorption spectrum [CH_3_CN, λ_max_, nm (εM, M^–1^ cm^–1^)]: 364 (4500), 483 (2500), 650 (1000). ESI-MS positive mode: [M]^+^ = 646.97 *m*/*z*. Elemental Analysis Calcd for C_16_H_36_CuF_6_Fe_2_N_6_O_2_PS_4_: C, 24.24; H, 4.58; N, 10.60; Found: C, 24.28; H, 4.56; N, 10.50; EPR (MeCN): *g*_*x*_ = 2.03, *g*_*y*_ = 2.02, *g*_*z*_ = 2.03.

### Synthesis of [(NO)­Fe­(bme-dame)-Cu^I^-(bme-dame)­Fe­(NO)]­[BF_4_], [FeCuFe]­[BF_4_]

A similar procedure was used as in the synthesis of [FeCuFe]­[PF_6_] by using [Cu­(CH_3_CN)_4_]­BF_4_. Yield: 65 mg (88%). FTIR (CaF_2_ windows, CH_3_CN, 23 °C): ν­(NO) = 1695 cm^–1^ (s). UV–vis absorption spectrum [CH_3_CN, λ_max_, nm (εM, M^–1^ cm^–1^)]: 364 (4500), 490 (2550), 658 (1100). ESI-MS positive mode: [M]^+^ = 646.97 *m*/*z*. Elemental Analysis Calcd for C_16_H_36_BCuF_4_Fe_2_N_6_O_2_S_4_: C, 26.15; H, 4.94; N, 11.44; Found: C, 26.10; H, 4.89; N, 11.50.

[FeCuFe]­[BF_4_] can also be synthesized using Cu­(II) salt, Cu­(BF_4_)_2_, which gives the desired product along with [Fe_2_(NO)_3_]­[BF_4_] as a side product.

### [VO­(bme-dach)-Cu^I^–VO­(bme-dach)]­(BF_4_)_2_, [VCuV]­[BF_4_]

In separate vials, VO­(bme-dach) (59.3 mg, 0.208 mmol) was suspended in 3 mL of CH_3_CN and [Cu^II^(MeCN)_4_]­(BF_4_)_2_ (38.5 mg, 0.096 mmol) was dissolved in 3 mL of CH_3_CN. The [Cu^II^(MeCN)_4_]­(BF_4_)_2_ solution was added dropwise to the VO­(bme-dach) suspension, after which a color change from blue-green to brown was observed. The mixture was then stirred for 2 days at room temperature under N_2_. After filtration of the reaction solution through a small Celite plug, brown crystals (42.2 mg, 61%) were grown by vapor diffusion (Et_2_O/CH_3_CN). ESI-MS positive mode: [M]^+^ = 632.99 *m*/*z*. Anal. Calcd for C_18_H_36_N_4_O_2_S_4_V_2_BF_4_Cu + 0.5 Et_2_O (F.W. 758.06): C, 31.69; H, 5.45; N, 7.39, found: C, 31.46; H, 5.42; N, 7.61.

### [VO­(bme-dach)-Cu^I^–VO­(bme-dach)]­(PF_6_), [VCuV]­[PF_6_]

In separate vials inside a glovebox filled with Ar, VO­(bme-dach) (49.7 mg, 0.174 mmol) was suspended in 7 mL of CH_3_CN and [Cu^I^(MeCN)_4_]­(PF_6_) (32.2 mg, 0.086 mmol) dissolved in 2 mL of CH_3_CN. The [Cu^I^(MeCN)_4_]­(PF_6_) solution was added dropwise to the VO­(bme-dach) suspension, and the mixture was stirred for 1.5 h, after which the volume was reduced to 3 mL in vacuo, followed by stirring for an additional 25 h. The reaction solution was then filtered through a small Celite plug. Brown crystals (52.9 mg, 79%) were obtained by vapor diffusion into the filtrate solution (Et_2_O/CH_3_CN). UV–vis absorption spectrum [CH_3_CN, λ_max_, nm (ε_M_, M^–1^ cm^–1^)]: 250 (1680), 430 (670). IR (V≡O): 991 cm^–1^. ESI-MS positive mode: [M]^+^ = 632.99 *m*/*z*. EPR (MeCN): *g*_*x*_ = *2.02, g*_*y*_ = 1.94, *g*_*z*_ = 1.96; *A*_*x*_ = 58, *A*_*y*_ = 220, *A*_*z*_ = 58.

### Physical Measurements

Solution and solid infrared spectra were recorded on a Bruker Tensor 37 Fourier transform IR (FTIR) spectrometer in a CaF_2_ solution cell with a 0.2 mm path length and as thin films, respectively. ^1^H and ^19^F spectra were recorded using the Bruker Avance NEO 400 MHz system with an automated tuning 5 mm broadband iProbe (Acend magnet). Electrospray ionization mass spectrometry (ESI-MS) was performed by the Laboratory for Biological Mass Spectrometry at Texas A&M University. Room-temperature electronic absorption spectra were obtained using a Shimadzu UV-1601PC Spectrophotometer. Low-temperature ultraviolet–visible (UV–Vis) spectroscopy was performed on an Agilent Technologies Cary Series UV–Vis NIR 5000 spectrometer equipped with an ice-cold water chilling system and a magnetic stirrer. Elemental analyses were performed using an Atlantic Microlab, Inc. Mössbauer spectrometer (MS4) with a ^57^Co/Rh radiation source, and the spectra were fitted with the WMOSS software (both from SEE Co.).

### X-ray Crystallography

Single-crystal X-ray diffraction measurements were carried out at a low temperature employing a (three circle or kappa) Bruker AXS (Quest or Venture) with IμS source and a Photon III area detector diffractometer for (Mo Kα radiation, λ = 0.71073 Å or Cu Kα radiation, λ = 1.54178 Å) (NSF-CHE- 9807975, NSF-CHE-0079822 and NSF-CHE-0215838). Crystals (for color, habit, and size, see corresponding CIF files) were mounted on a nylon or Kapton loop and cooled in a cold nitrogen stream (OXFORD Cryosystems (700 or 800) to 110 (2) K). Bruker AXS APEX 3 ^72^ software was used for data collection and reduction. Absorption corrections were applied using SADABS.^73^ Space group assignments were determined by the examination of systematic absences, E-statistics, and successive refinement of the structures. Structures were solved using SHELXT^74^ and refined by least-squares refinement on F2 followed by difference Fourier synthesis (OLEX2, SHELXL).^75,77^ All hydrogen atoms were included in the final structure factor calculation at idealized positions and were allowed to ride on the neighboring atoms with relative isotropic displacement coefficients. Thermal parameters were refined anisotropically for all nonhydrogen atoms to convergence. The MERCURY 2022.1.0 ^76^ interface was used for structure visualization, analysis of bond distances and angles, and drawing ORTEP plots. OLEX2 ^77^ interface was used for structure overlay.

### Cyclic Voltammetry

Cyclic voltammograms were recorded on a CHI600E electrochemical analyzer (HCH Instruments, Inc.). All experiments were performed under an Ar blanket in CH_3_CN or DMF solutions containing a 0.1 M [*n*Bu_4_N]­[PF_6_] electrolyte and 2.0 mM analyte at room temperature. A 0.071 cm^2^ glassy carbon disk was used as the working electrode, platinum wire as the counter electrode, and Ag(0)/AgCl as the reference electrode. The working electrode was cleaned by using polishing pads with alumina slurries after each experiment. All potentials were referenced to the Fc/Fc^+^ couple at 0.00 V.

### Magnetic Measurements (Solid Phase)

DC magnetic measurements were performed on freshly prepared, crushed crystalline samples of **[FeCuFe]­[PF**_**6**_ **]** and **[VCuV]­[BF**_**4**_ **]** (Figure 3). The samples were placed inside a plastic bag, which was tightly rolled and placed inside a plastic straw, and measured at a temperature range from 2 to 300 K in an applied magnetic field of 10,000 and 1000 Oe on a Quantum Design SQUID, Model MPMS with a 7 T magnet. The diamagnetic contribution from the plastic bag was subtracted from the raw data. The molar paramagnetic susceptibilities (χ*_M_T*) were obtained by using Pascal’s constants to subtract the diamagnetic corrections of the atoms from the experimental susceptibilities.^46^ A paramagnetic (*S* = 1/2) impurity of 1% contribution was added to the fits of **[FeCuFe]­[PF**_**6**_ **]** and **[VCuV]­[PF**_**6**_ **]**. Magnetic (SQUID) data was fitted using the PHI software.^48^

### Calculation of Solution-Phase Magnetic Susceptibility Using the Evans Method

The magnetic susceptibility of **[FeCuFe]­[PF**_**6**_ **]** was determined by the Evans method.^47^ ^19^F NMR was measured in a 400 MHz NMR machine at 293 K. In an NMR tube, 14.0 mg of **[FeCuFe]­[PF**_**6**_ **]** was taken and dissolved in 0.5 mL of deuterated CD_3_CN, and 1.0 μL of C_6_F_6_ was added under an argon atmosphere. Next, an inner tube containing a mixture of 0.5 mL of deuterated THF and 1.0 μL of C_6_F_6_ was placed into the NMR tube, and then, ^19^F NMR of the sample was measured at 295 K. The calculated diamagnetic susceptibility, **χ**D, of **[FeCuFe]­[PF**_**6**_ **]** was −0.000327. The complex **[FeCuFe]­[PF**_**6**_ **]** shows the magnetic moment value of 2.57 μ_B_ consistent with the presence of 2 unpaired electrons (Figure S15).

### Computational Methodology

All calculations were performed using the Gaussian 16, revision C.01, package.^78^ The geometry optimizations were initiated from the coordinates taken from the crystal structure of the complexes using Becke’s three-parameter hybrid functional B3LYP,^79^ TPSSH^80^ and TPSSTPSS^80^ in conjunction with Truhlar and co-workers’ SMD solvation model^81^ in acetonitrile. We have also performed calculations in the gas phase to ensure that there is no significant effect of solvent or packing interactions on the stability of the crystal geometry (Figure S17). Ahlrichs and co-workers’ triple-ζ quality DEF2-TZVP basis set^82^ was employed for Fe and Cu, while Petersson and co-workers’ 6–311++G** basis set,^83^ including polarization and diffusion functions, was used for all other atoms. For cases where SCF convergence was challenging, a quadratic convergence algorithm was applied. Stationary points were confirmed by their calculated vibrational frequencies (zero imaginary frequency). We also utilized the D2 version of Grimme’s dispersion hybrid functional UB97D,^84^ which provided similar results. **[FeCuFe]** ^**0**^ and **[FeCuFe]** ^**–1**^ were optimized with a spin multiplicity of quartet and quintet. Due to convergence difficulties in the TPSSTPSS calculations, optimization was carried out at the TPSSH level for **[FeCuFe]** ^**0**^ and **[FeCuFe]** ^**–1**^. UV–vis spectra were calculated by TDDFT calculation. IR spectra were calculated from the optimized geometry of **[FeCuFe]** ^**+**^, **[FeCuFe]** ^**0**^, and **[FeCuFe]** ^**–1**^ at the UTPSSH level of theory (Figure S27). Visualization of the molecular orbitals and corresponding diagrams was performed using Chemcraft software.^85^

### Electrochemical Assignments of Reduction Products by Vibrational Spectroscopy

The chemical reduction of **[FeCuFe]­[PF**_**6**_ **]** was done using cobaltocene (Cp_2_Co, *E*_1/2_ = −1.33 V) or KEt_3_BH inside the glovebox at −20 °C. In a 20 mL vial, a 20 mmol solution of **[FeCuFe]­[PF**_**6**_ **]** in dry acetonitrile was cooled down to −20 °C. Then, equivalents of reductant were added and stirred for 10 min at 0 °C before transferring the solution to an IR cell for measurement. This was followed for each reduction step (Figure 6).

### Low-Temperature Electronic Absorption Spectroscopic Studies of Reduction Products

To probe electronic communication between the two {FeNO}^7^ units upon sequential reduction, low-temperature UV–vis spectra were recorded in MeCN at −30 °C, focusing on the charge transfer region using a 500 μM sample concentration. Since one-electron reduction of this system can generate either a localized or a delocalized mixed-valent species, spectral changes were monitored during stepwise addition of reductant (Figure S26).

In a typical experiment, a 500 μM MeCN solution (3 mL) of was added into a 1 cm path length cuvette inside the glovebox and was sealed using a rubber septum. Upon cooling down inside the UV–vis cryostat stabilized at −30 °C, equivalents of reductant were added and stirred for 30 s before being monitored by UV–vis spectroscopy.

### CW-EPR Measurement

Samples for EPR studies were prepared at concentrations of 1 mM and 10 mM in dry acetonitrile and were sealed with a rubber septum inside the glovebox. EPR tube was taken out of the box, frozen in liquid nitrogen, and then shipped to the University of Alabama for EPR studies. CW EPR experiments were performed at the University of Alabama EPR facility using a Bruker ELEXSYS E540 X-band spectrometer (Bruker-Biospin, Billerica, MA). Cryogenic measurements were made on a frozen solution (CH_3_CN) for **[FeCuFe]­[PF**_**6**_ **]** and **[VCuV]­[PF**_**6**_ **]** using a ColdEdge Stinger closed-loop liquid helium cryosystem inserted into an Oxford ESR900 cryostat. A LakeShore 336 temperature controller was used to regulate sample temperature. EPR simulations were calculated using SpinCount (ver. 8.2.9361.22857) developed by Professor Michael Hendrich at Carnegie Mellon University by utilizing the general spin Hamiltonian as shown in eqs 2–4.

For both **[FeCuFe]­[PF**_**6**_ **]** and **[VCuV]­[PF**_**6**_ **]**, electronic spins are equivalent (*S*_1_ = *S*_2_ = 1/2). However, for **[VCuV]­[PF**_**6**_ **]**, nuclear spins (*I*_1_ = *I*_2_ = 7/2) were included to account for the increased line width. For these complexes, the spin Hamiltonian can be expressed as follows

2 $$Ĥ=-2JS_{1}S_{2}+{Ĥ}_{dip}+{Ĥ}_{1}+{Ĥ}_{2}$$

In the above expression, ***J*** represents the isotropic Heisenberg–Dirac–van Vleck electronic exchange constant between each paramagnetic center (PC). Dipolar interactions are introduced by *Ĥ*_dip_, and *Ĥ*_*i*_ (*i* = 1, 2) are the intrinsic spin Hamiltonians corresponding to each PC. The dipolar spin–spin interaction is calculated using eq 3

3 $${Ĥ}_{\mathrm{dip}}=\frac{\mu_{0}}{4\pi }\beta^{2}\left[\frac{\left(S_{1}\cdot g_{1}\right)\cdot \left(S_{2}\cdot g_{2}\right)}{r^{3}}-\frac{3\cdot \left(S_{1}\cdot g_{1}\cdot r\right)\cdot \left(S_{2}\cdot g_{2}\cdot r\right)}{r^{5}}\right]$$

where ***r*** represents the internuclear vector, ***g***_***1***_ and ***g***_***2***_ are the intrinsic *g*-tensors for individual vanadyl ions, and b is the electron Bohr magneton. The dipolar interaction is calculated using the distance separating the two PCs (r). Eq 4 presents the spin Hamiltonian for individual site *Ĥ*_*i*_ (*i* = 1, 2). As the PC in both **[FeCuFe]­[PF**_**6**_ **]** and **[VCuV]­[PF**_**6**_ **]** has an electronic spin (*S*_1_ = *S*_2_ = 1/2), there are no intrinsic zero-field splitting (SDS) contributions from individual sites in the Hamiltonian. Consequently, the energy for each site simplifies down to the sum of electronic Zeeman and nuclear hyperfine terms (for ^51^V PCs).

4 $${Ĥ}_{i}={\beta S}_{i}\cdot {\mathrm{g̃}}_{i}\cdot B+S_{i}\cdot {Ã}_{i}\cdot I_{i}\left(i=1,2\right)$$

Here, ***g̃***_***i***_ and ***Ã***_***i***_ represent the intrinsic ***g*** and ***A***-tensors for individual sites, respectively. The hyperfine coupling form is treated by the second-order perturbation theory.^86,87^

Simulations were generated with consideration of all intensity factors, both theoretical and experimental, to allow for the determination of species concentration. The only unknown factor relating the spin concentration to signal intensity was an instrumental factor that is specific to the microwave detection system. This factor was determined by a 1.0 mM Cu­(EDTA) spin standard prepared from a copper atomic absorption standard solution purchased from Sigma-Aldrich. All samples were prepared in custom-made 4 mm quartz EPR tubes made at the UA glassblowing facility. For all analytical EPR measurements, data was collected under nonsaturating conditions at a modulation frequency of 100 kHz (Figure 7).

## Supplementary Material



## Data Availability

The data underlying this study are available in the published article and its Supporting Information.

## Notes

Supporting crystal structure data and data collection parameters, along with additional spectroscopic and magnetic data and Cartesian coordinates of optimized geometries (PDF)
