# Supplementary material for: Twists, Turns, and Spins: Copper-Guided Electronic Communication in Sulfur-Bridged Trimetallic Complexes
Source: ACS Org Inorg Au. 2026 Jul 31;6(5):636–53. doi: 10.1021/acsorginorgau.6c00057 (PMC13650954; doi:10.1021/acsorginorgau.6c00057)
Supplement: Supplementary file 1 [file gg6c00057_si_001.pdf]

Twists, Turns, and Spins: Copper-Guided Electronic Communication in Sulfur-Bridged Trimetallic Complexes

Akhil K. Singh,[†] Dakota D. Jones,[†] Aruzhan Abdikaiym,[‡] Naushad Ahmed,[†] Sarnali Sanfui,[†]Joseph H. Reibenspies[†], Yisong Guo,[§] Brad S. Pierce,[‡] Kim R. Dunbar,[†] Michael B. Hall,[†] and Marcetta Y. Darensbourg[*†]

†Department of Chemistry Texas A&M University, College Station, TX 77843

‡ Department of Chemistry & Biochemistry, University of Alabama, Tuscaloosa, Alabama 35487, USA

§ Department of Chemistry, Carnegie Mellon University, Pittsburgh, Pennsylvania 15213, USA

To whom correspondence should be addressed:

Marcetta Y. Darensbourg
E-mail: marcetta@chem.tamu.edu

Contents	Page
Figure S1. Infrared spectra of (NO)Fe(bme-dame) and [FeCuFe][PF_6] in CH_3CN at 298 K.	S3
Figures S2 & S3. ESI-MS spectra of [FeCuFe][PF_6] and [VCuV][BF_4].	S4
Figure S4. UV-vis spectra of [FeCuFe][PF_6] and [FeCuFe][BF_4] in CH_3CN at 298K.	S5
Figure S5. UV-vis spectra of [VCuV][PF_6] in CH_3CN at 298K.	S5
Tables S1-S2. Crystal data and data collection parameters.	S6-S8
Table S3. Selected structural and geometrical parameters.	S9
Figures S6-S11. Packing diagram of [FeCuFe][PF_6], [FeCuFe][BF_4] and [VCuV][BF_4] obtained from X-ray crystallography.	S10-S13
Figure S12. Selected bond angles of [FeCuFe][PF_6], [VCuV][BF_4], [FeNiFe][2BF_4] and [VNiV][2BF_4].	S14
Figure S13-S16 elipsoid plots	S14-S16
Figure S17. Molecular structures (at 100 K) of parent Fe(NO)dame and V(O)dame ligands showing S-M-S angles. H-atoms have been omitted for clarity.	S17
Figure S18. Overlay of Molecular structures of [FeNiFe][BF_4] and [FeCuFe][PF_6].	S17
Figure S19. ^{19}F NMR spectrum of [FeCuFe][PF_6] at 293 K, referenced to hexafluorobenzene for Evans Method determination of magnetic susceptibility.	S18
Figure S20. Zero field ^{57}Fe Mössbauer spectra of [FeCuFe][PF_6] recorded at 5 K.	S18
Figure S21. UTPSSTPSS/6-311++G**/def2TZVP optimized triplet state geometries of $[FeCuFe]^+$ and $[VCuV]^+$.	S19
Table S4. Relative energies and spin-squared operator, $\langle S^2 \rangle$ of the plausible spin multiplicities of $[FeCuFe]^+$.	S19
Table S5. Relative energies of the plausible spin multiplicities of $[VCuV]^+$. All the ΔE+ZPE values are relative to triplet (3) state.	S20
Table S6. A comparison of IR stretching vibration calculated from UTPSSH/6-311+G**/def2TZVP optimized geometries of $[FeCuFe]^+$, $[FeCuFe]^0$, and $[FeCuFe]^{-1}$.	S20
Figure S22. Frontier molecular orbitals of $[FeCuFe]^+$ and $[VCuV]^+$ (triplet).	S21
Figure S23. Frontier molecular orbitals of UTPSSH/6-311+G**/def2TZVP optimized geometry of $[FeCuFe]^0$ and $[FeCuFe]^{-1}$.	S22
Figure S24: Qualitative molecular orbital diagrams and spin density plots (isovalue = 0.003 au) for (NO)Fe(bme-dame) ligand and (O)V(bme-dame) ligand.	S22
Figure S25. Coupling models and spin density distribution plots of $[FeCuFe]^+$, $[FeNiFe]^{2+}$, and $[VCuV]^+$ calculated for open-shell singlet, and triplet states.	S23
Figure S26. Cyclic voltammogram (in CH_3CN at 295 K) of [FeCuFe][PF_6].	S24
Figure S27. IR spectral changes observed (in CH_3CN at 295 K) during the reaction of a 10 mM solution of [FeCuFe][PF_6] with (A) 1.0-4.0 equivalent of 1 M (THF) $KHBEt_3$.	S25
Figures S28 & S29. Cyclic voltammogram (in CH_3CN at 295 K) of [VCuV][PF_6].	S26
Figure S30. Electronic absorption spectral changes observed (in CH_3CN at −30 °C) during the reaction of a 0.5 mM solution of [FeCuFe][PF_6] with $KHBEt_3$.	S27
Figure S31. Absorption spectrum and oscillator strengths along with main orbital configurations, obtained from TD-DFT calculations at the optimized geometry of $[FeCuFe]^0$ and $[FeCuFe]^{-1}$.	S28
Table S7. EPR simulation parameters for triplet (S = 1) [M'CuM'][PF_6] complexes.	S29
Cartesian Coordinates of optimized geometries.	S29-S33

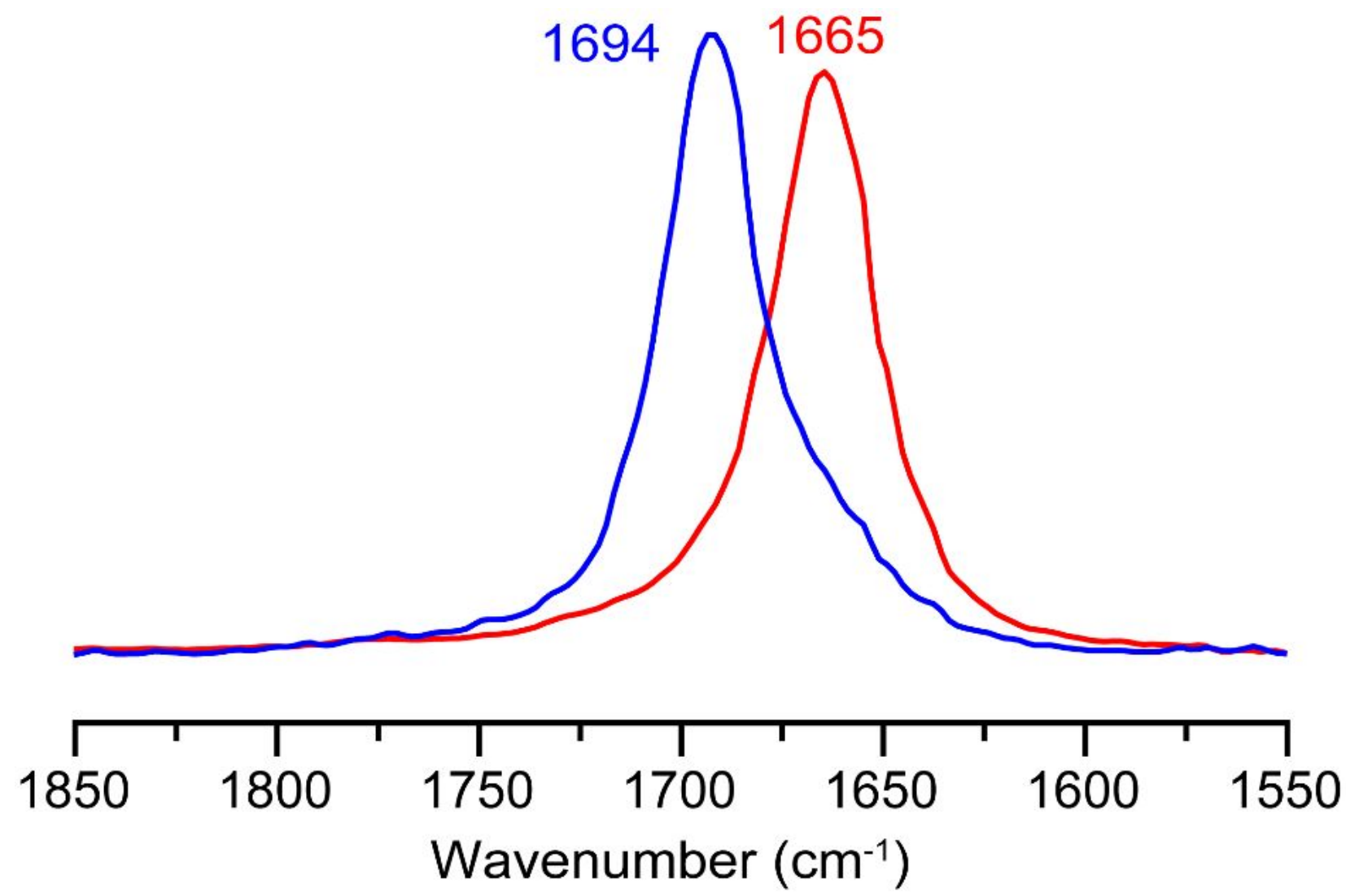

Figure S1. Infrared spectra of **(NO)Fe(bme-dame)** (red) and **[FeCuFe][PF$_6$]** (blue) in CH_3CN at 298 K.

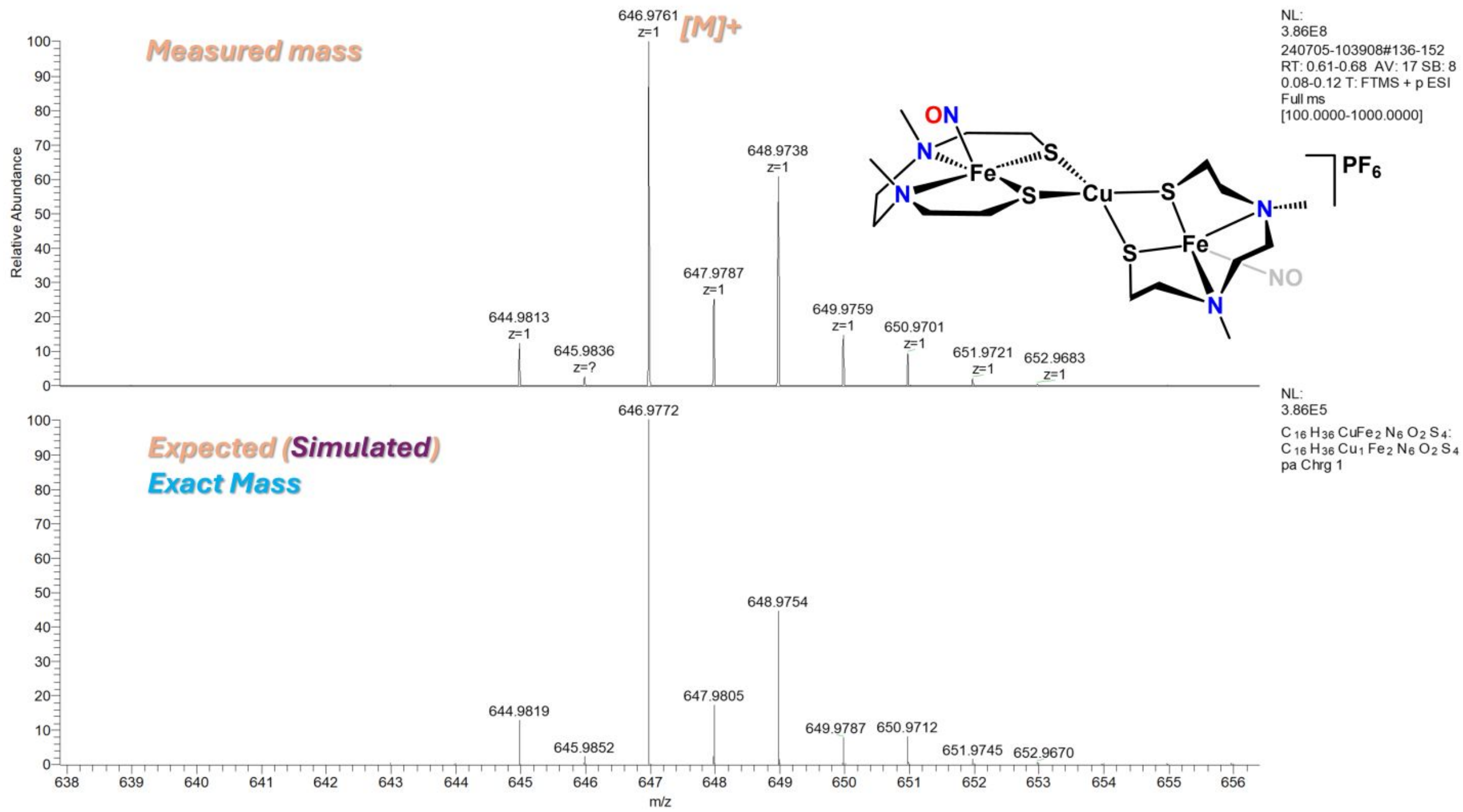

Figure S2. Isotopic distribution pattern of the experimental (top) and simulated (bottom) ESI-MS spectrum (positive ion mode) of **[FeCuFe][PF_6].**

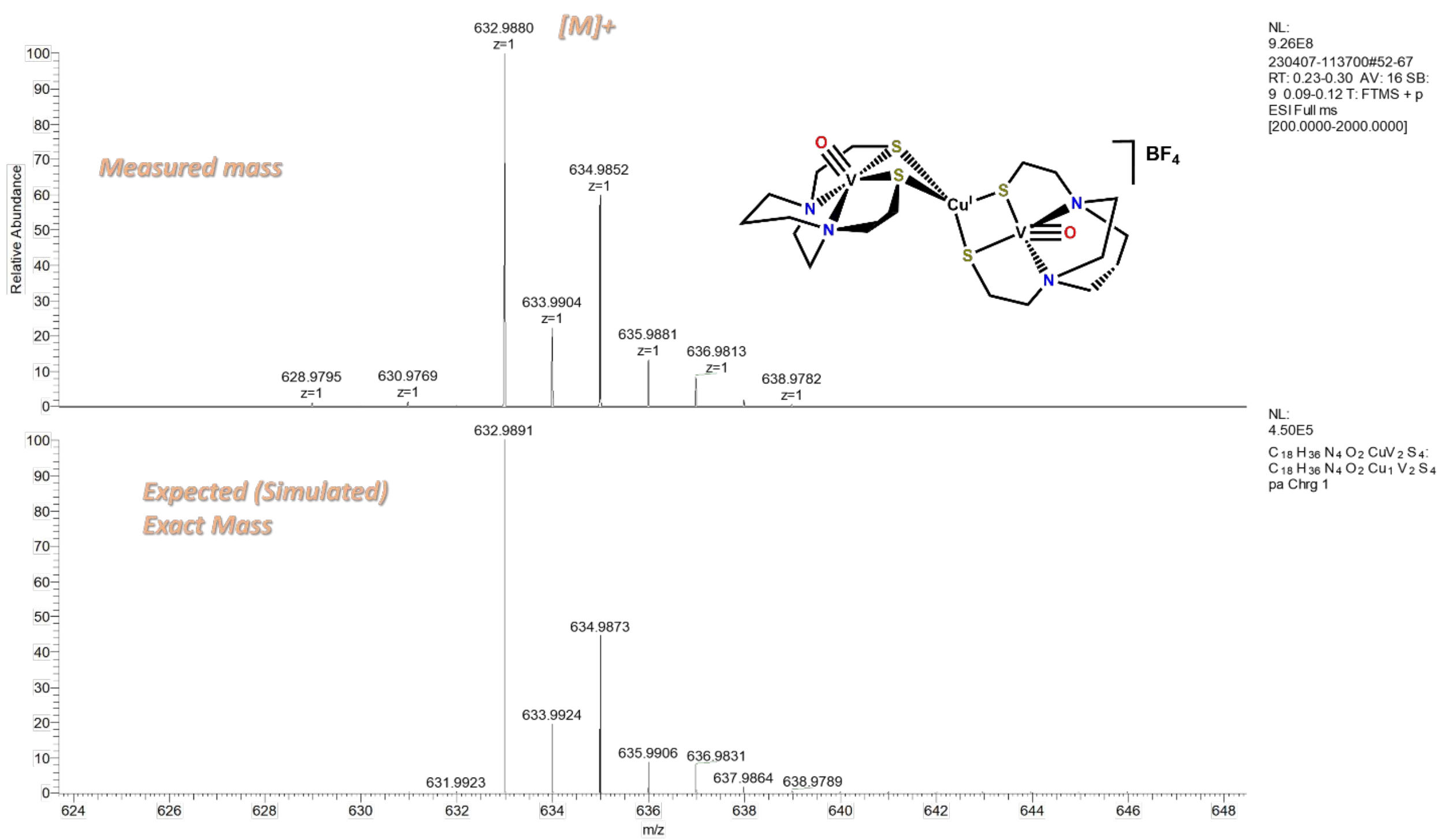

Figure S3. Isotopic distribution pattern of the experimental (top) and simulated (bottom) ESI-MS spectrum (positive ion mode) of **[VCuV][BF_4].**

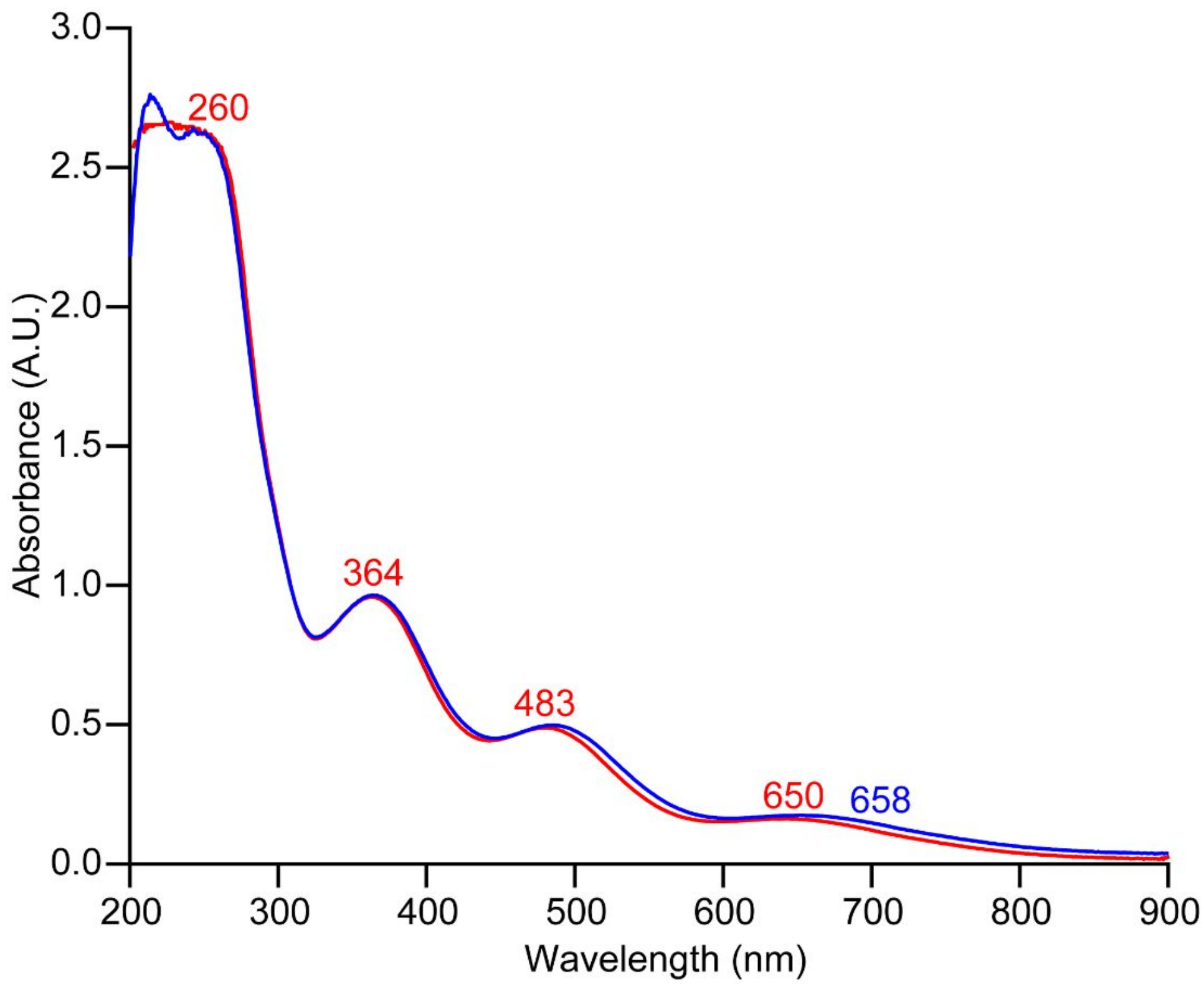

Figure S4. UV-vis spectra of **[FeCuFe][PF_6]** (red) and **[FeCuFe][BF_4]** (blue) in CH_3CN at 298K.

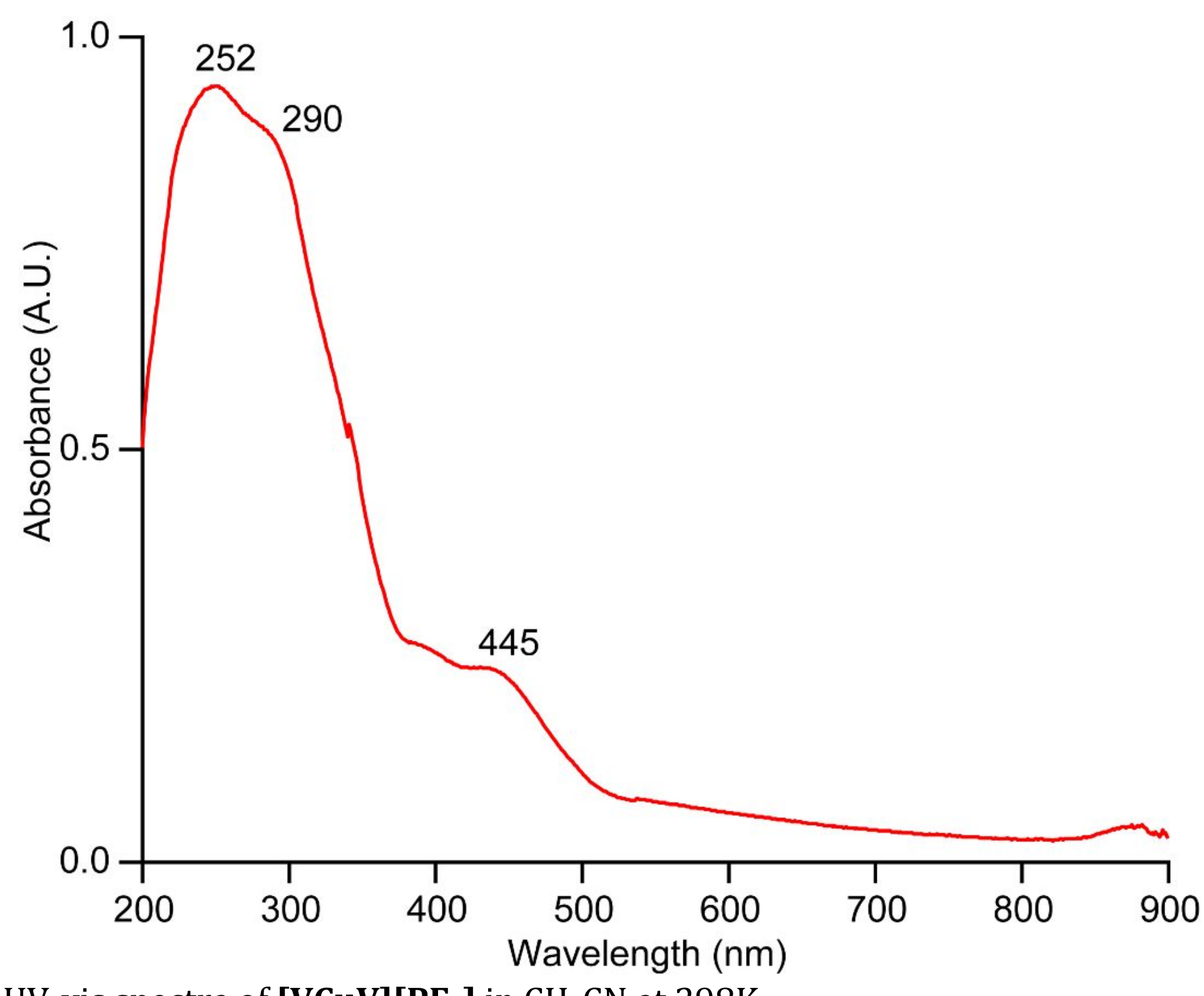

Figure S5. UV-vis spectra of **[VCuV][PF_6]** in CH_3CN at 298K.

Table S1. Crystal data and data collection parameters.

	**[FeCuFe][PF$_6$]**	**[FeCuFe][BF$_4$] •CH$_3$CN**	**[VCuV][BF$_4$]**	**[VCuV][PF$_6$] •0.45 Et$_2$O**
Formula	$C_{16}H_{36}CuF_6Fe_2N_6O_2PS_4$	$C_{18}H_{39}BCuF_4Fe_2N_7O_2S_4$	$C_{18}H_{36}BCuF_4N_4O_2S_4V_2$	$C_{18}H_{36}BCuF_4N_4O_2S_4V_2$•0.45 Et_2O
T, (K)	100.00(10)	100.00(10)	100(2)	110
Formula weight	785.27	775.874	720.98	812.49
Crystal system	Monoclinic	Monoclinic	Monoclinic	Monoclinic
Space group	$P2_1/c$	$P2_1/c$	$P2_1/n$	$P2_1/n$
a, Å	15.6472(2)	17.1793(3)	7.6100(15)	7.6525(6)
b, Å	11.88730(10)	12.7678(2)	27.710(6)	28.918(2)
c, Å	15.9521(2)	14.2100(2)	14.340(3)	14.4370(12)
α , deg	90	90	90	90
β, deg	102.939(1)	101.434(2)	98.66(3)	100.366(4)
γ, deg	90	90	90	90
V, Å^3	2891.86(6)	3054.99(9)	2989.4(11)	3142.7(4)
Radiation (λ, Å)	Cu Kα (λ = 1.54184)	Cu Kα (λ = 1.54184)	Cu Kα (λ = 1.54184)	Cu Kα (λ = 1.54178)
Z	4	4	4	4
d_{calcd}, g.cm^{-3}	1.804	1.687	1.602	1.717
F(000)	1603.6	1586.1	1472.0	1660.0
μ, mm^{-1}	15.748	11.372	1.645	9.223
No. of unique data	6218	6028	5498	6381
No. of parameters, refined	347	357	319	418
GOF on F^2	1.071	1.028	1.117	1.111
$R1^a$ [$I > 2\sigma(I)$]	0.0457	0.0410	0.0688	0.0672
$R1^a$ (all data)	0.0477	0.0454	0.0901	0.0816
$wR2^b$(all data)	0.1172	0.1044	0.1336	0.1499

$$^{a}R1 = \frac{\sum||F_o|-|F_c||}{\sum|F_o|}; \quad ^{b}wR2 = \sqrt{\frac{\sum[w(F_o{}^2-F_c{}^2)^2]}{\sum[w(F_o{}^2)^2]}}$$

Table S2. Selected Bond Distances (Å) and Bond angles (°) of complexes.

**Bond length, (Å)**	**[FeCuFe][PF_6]**	**[FeCuFe][BF_4] •CH_3CN**	**[VCuV][BF_4]**	**[VCuV][PF_6]•0.45 Et_2O**
Cu1-Fe1	2.8938(5)	2.8179(7)		
Cu1-Fe2	2.8681(5)	2.9021(7)		
Fe1-S1	2.2734(6)	2.2696(9)		
Fe1-S2	2.2596(6)	2.2454(9)		
Fe1-N1	2.0676(19)	2.065(3)		
Fe1-N2	2.048(2)	2.069(3)		
Fe1-N3	1.690(2)	1.694(3)		
Fe2-S3	2.2351(7)	2.2500(9)		
Fe2-S4	2.2569(7)	2.2710(9)		
Fe2-N4	2.055(2)	2.073(3)		
Fe2-N5	2.049(2	2.060(3)		
Fe2-N6	1.688(2)	1.690(3)		
O1-N3	1.166(3)	1.179(4)		
O2-N6	1.177(3)	1.184(4)		
Cu1-S1	2.2639(6)	2.2776(9)	2.2878(19)	2.284(2)
Cu1-S2	2.3479(6)	2.3754(9)	2.3417(18)	2.343(2)
Cu1-S3	2.3497(7)	2.3547(9)	2.3002(19)	2.333(2)
Cu1-S4	2.2604(7)	2.2832(9)	2.3338(18)	2.292(2)
V1-S1			2.376(2)	2.373(2)
V1-S2			2.391(2)	2.382(2)
V1-N1			2.127(5)	2.133(5)
V1-N2			2.126(5)	2.118(4)
V1-O1			1.585(5)	1.592(5)
V2-S3			2.3942(18)	2.367(2)
V2-S4			2.372(2)	2.393(2)
V2-N3			2.119(5)	2.122(4)

V2-N4			2.121(4)	2.115(6)
V2-O2			1.595(4)	1.581(4)
Bond angles, (deg)				
Fe2-Cu1-Fe1	164.770(16)	161.21(2)		
S1-Cu1-Fe1	50.518(17)	51.58(2)		
S1-Cu1-Fe2	120.47(2)	127.13(3)		
S2-Cu1-Fe1	49.746(17)	50.36(2)		
S2-Cu1-Fe2	145.19(2)	143.29(3)		
S3-Cu1-Fe2	49.511(18)	49.35(2)		
S4-Cu1-Fe1	126.10(2)	116.46(3)		
S4-Cu1-Fe2	50.532(18)	50.23(2)		
O1-N3-Fe1	156.1(2)	150.6(3)		
O2-N6-Fe2	151.1(2)	156.1(3)		
S1-Cu1-S2	88.79(2)	86.47(3)	96.71(7)	96.60(6)
S1-Cu1-S3	116.54(3)	123.80(3)	138.24(6)	106.17(6)
S2-Cu1-S3	102.48(2)	102.20(3)	107.36(8)	109.05(6)
S1-Cu1-S4	140.76(3)	140.12(4)	106.85(7)	138.65(7)
S2-Cu1-S4	118.29(3)	115.24(3)	109.74(6)	107.80(6)
S3-Cu1-S4	86.52(3)	85.70(3)	138.24(6)	96.89(6)
V1-S1-Cu1			79.96(6)	80.20(6)
V1-S2-Cu1			78.57(6)	78.84(6)
V2-S3-Cu1			78.00(6)	77.93(5)
V2-S4-Cu1			77.80(6)	78.20(5)

Table S3. Selected structural and geometrical parameters.

Complex	FeCuFe•PF_6		FeCuFe•BF_4		VCuV•PF_6		VCuV•BF_4	
	core-I	core-II	core-I	core-II	core-I	core-II	core-I	core-II
**V/Fe-N (Å)**[a]	2.057(2)	2.052(2)	2.067(1)	2.066(1)	2.126(5)	2.118(5)	2.126(5)	2.120(5)
**V/Fe-S (Å)**[b]	2.266(1)	2.246(1)	2.257(1)	2.260(1)	2.378(2)	2.380(2)	2.383(2)	2.317(2)
**$\Delta N_2S_2^M$ (Å)**[c]	0.514	0.497	0.502	0.535	0.697	0.705	0.696	0.707
**M-M' (Å)**[d]	2.894(1)	2.868(1)	2.817(1)	2.902(1)	2.956(1)	3.001(1)	2.955(2)	2.997(2)
**∠Fe-N-O**[e]	156.1(2)	151.1(2)	156.1(1)	150.6(1)	---		---	
**M-M (Å)**[f]	5.711		5.644		5.921		5.907	
**Td angle (°)**[g]	87.45		85.68		80.67		80.35	
**Hinge angle (°)**[h]	72.42, 76.02		86.71, 89.37		63.19, 67.79		63.97, 68.92	
**Interplanar angle (°)**[i]	38.28		38.71		56.89		54.78	

[a] Average distance between metals (Fe or V) and nitrogen of N_2S_2 core. [b] Average distance between metals (Fe or V) and sulfur from N_2S_2 core. [c] Average displacement of metals (Fe or V) from the least-squares plane of N_2S_2 core. [d] Distance between Cu or Ni and terminal metals. [e] Fe-N-O angle. [f] Non-bonding distance between metals in N_2S_2 cores. [g] Angle between two tetrahedron plane ($M'S_2$) formed by copper. [h] the dihedral angle (θ) between the N_2S_2 and CuS_2 mean planes. [i] Angle between two intramolecular least-squares planes of N_2S_2 cores. Structural data for [FeNiFe] and [VNiV] is reproduced from ref 13 and 15 with permission of authors and in accordance with journal policy. Available under a CC-BY NC 3.0 license.

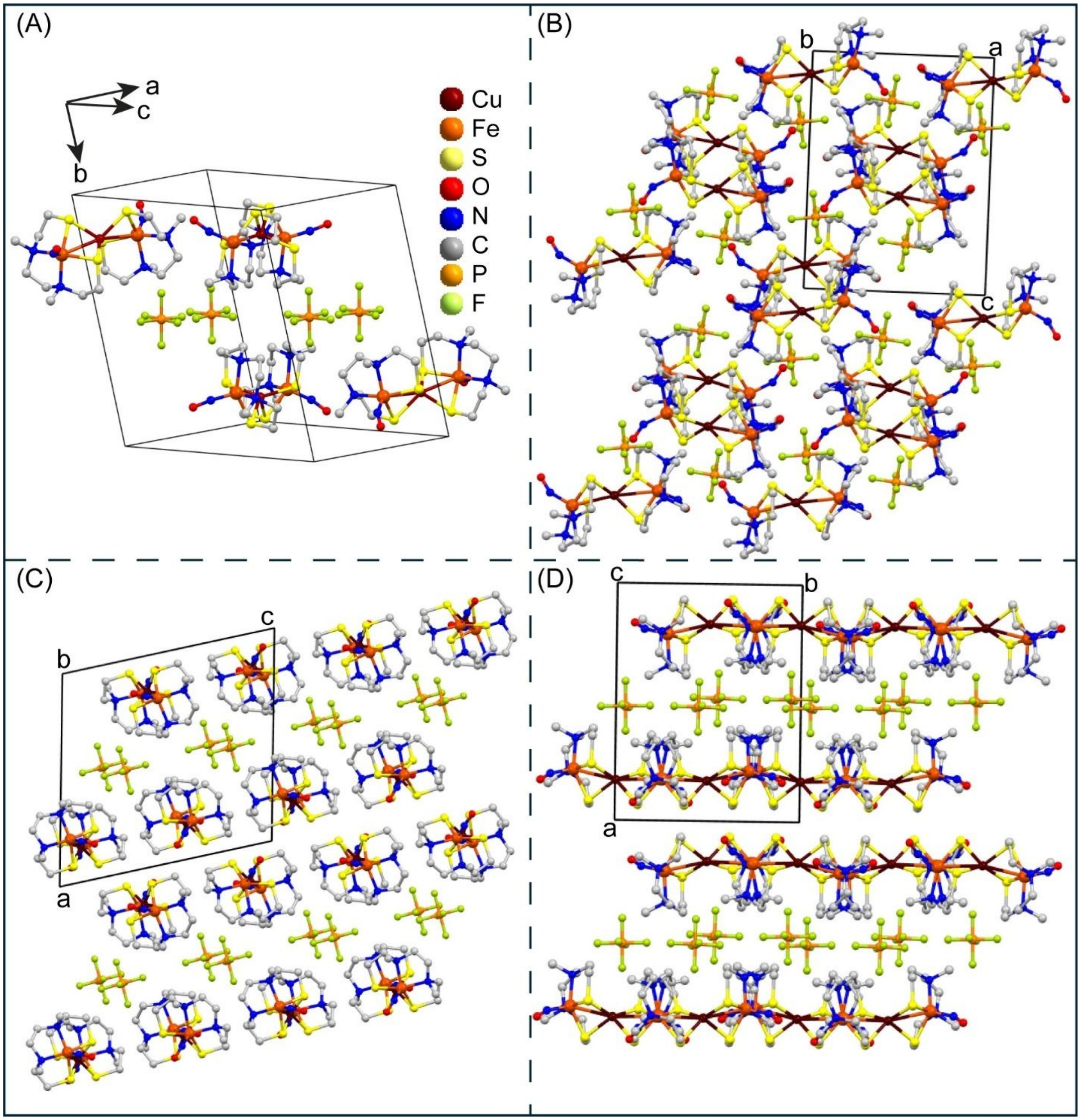

Figure S6. Extended packing of **[FeCuFe][PF$_6$]** in the (A) unit cell, (B) along bc plane, (C) along ac plane and (D) along ab plane at 100 K (H atoms have been omitted for clarity).

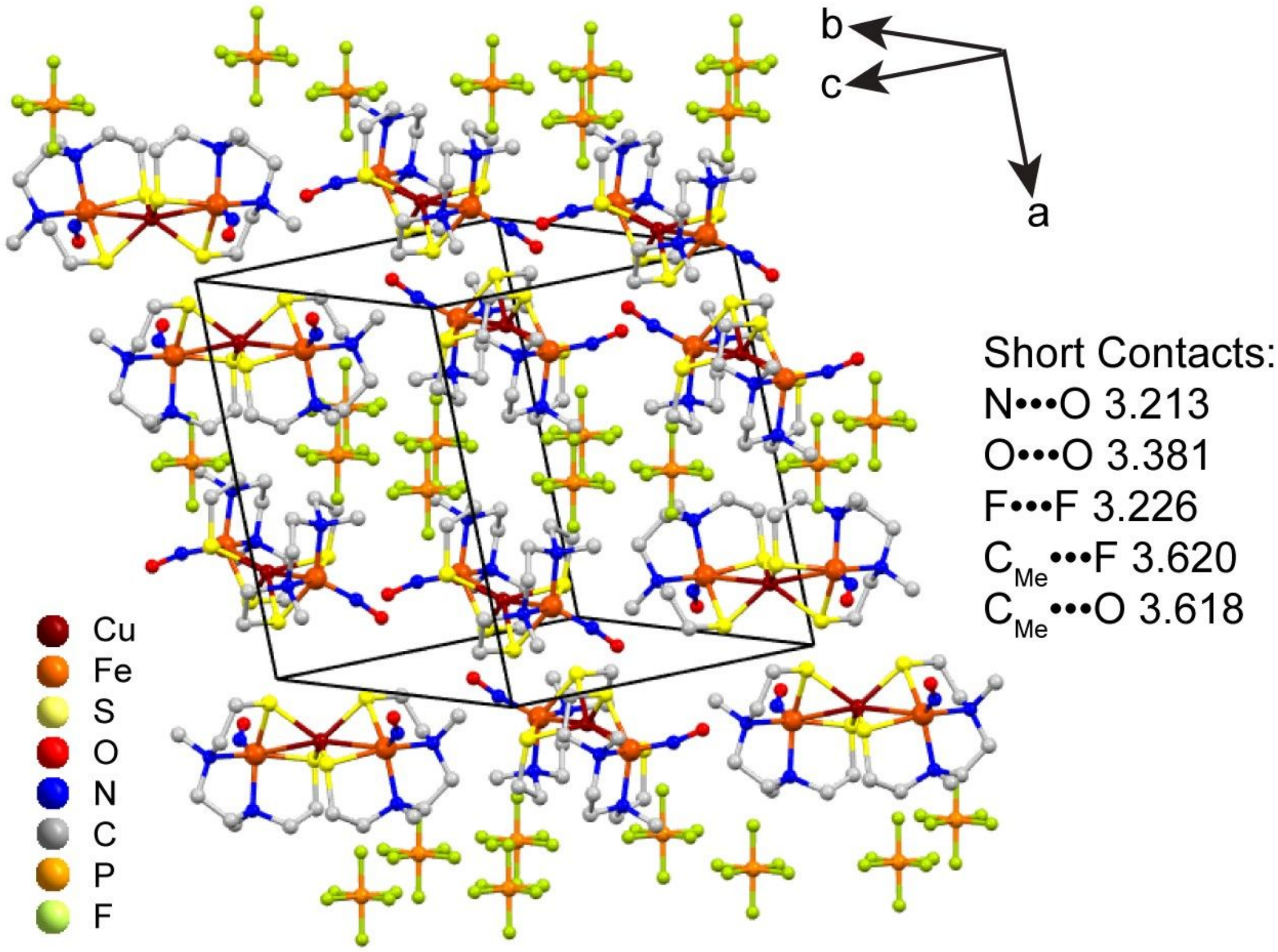

Figure S7. Extended packing of **[FeCuFe][PF$_6$]** in the unit cell at 100 K (H atoms have been omitted for clarity); Distances are in Å.

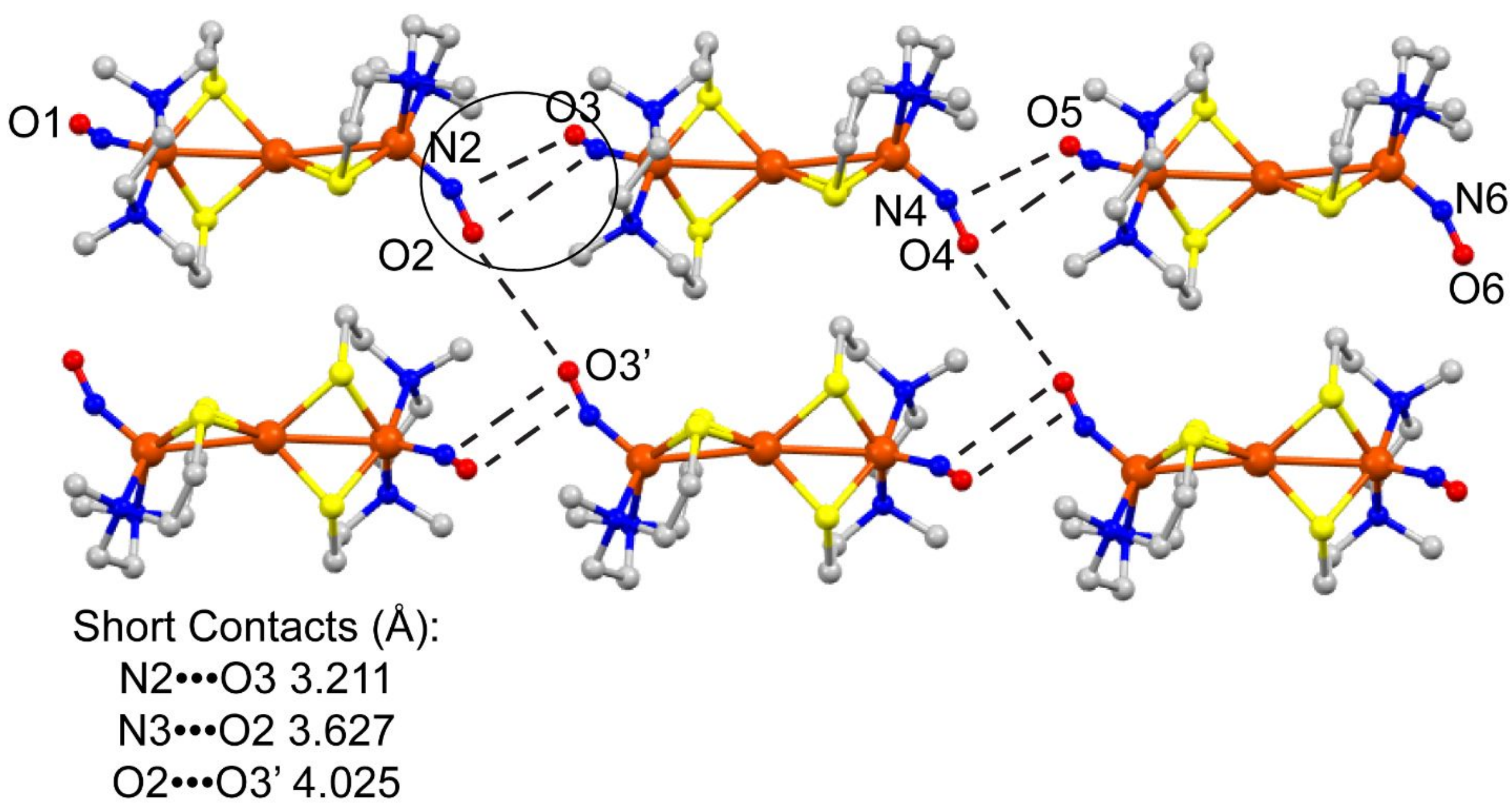

Figure S8. Linear chain structure of **[FeCuFe][PF$_6$]** showing short contacts with NO•••NO interactions at 100 K (H atoms and counter ions have been omitted for clarity).

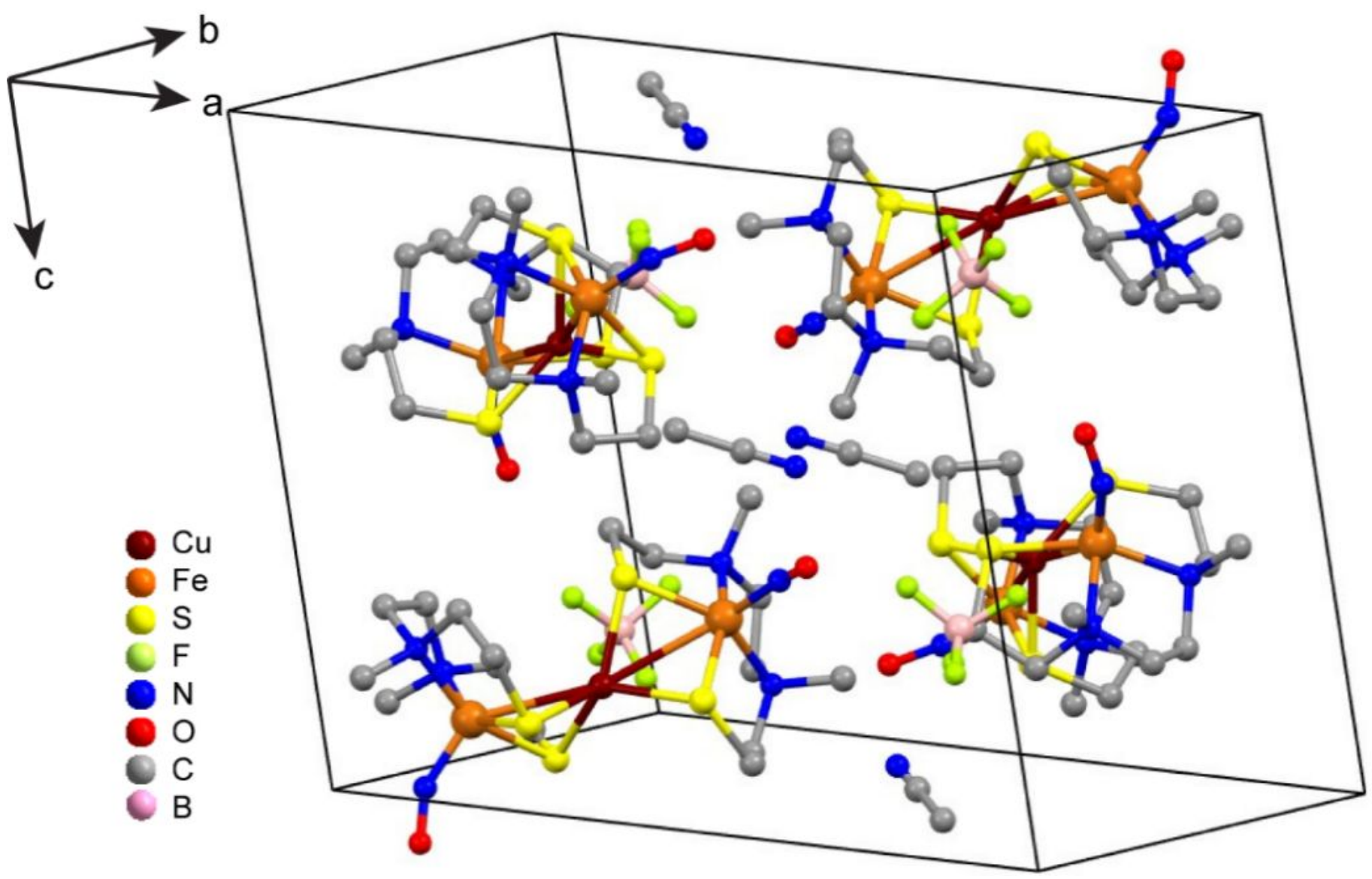

Figure S9. Diagram illustrating the packing of **[FeCuFe][BF$_4$] •CH$_3$CN** in the unit cell at 100 K (H atoms have been omitted for clarity).

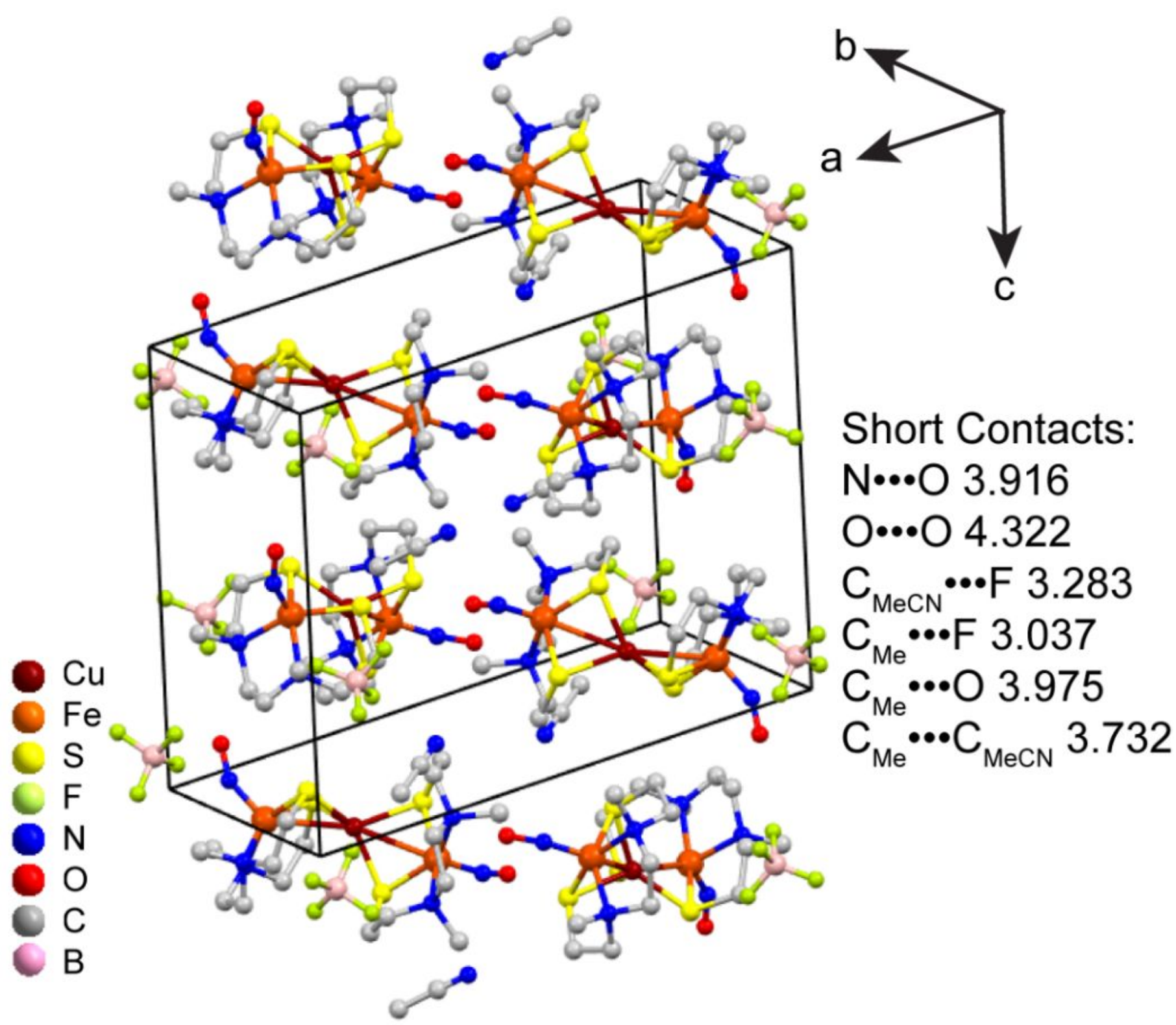

Figure S10. Extended packing of **[FeCuFe][BF$_4$] •CH$_3$CN** in the unit cell at 100 K (H atoms have been omitted for clarity); Distances are in Å.

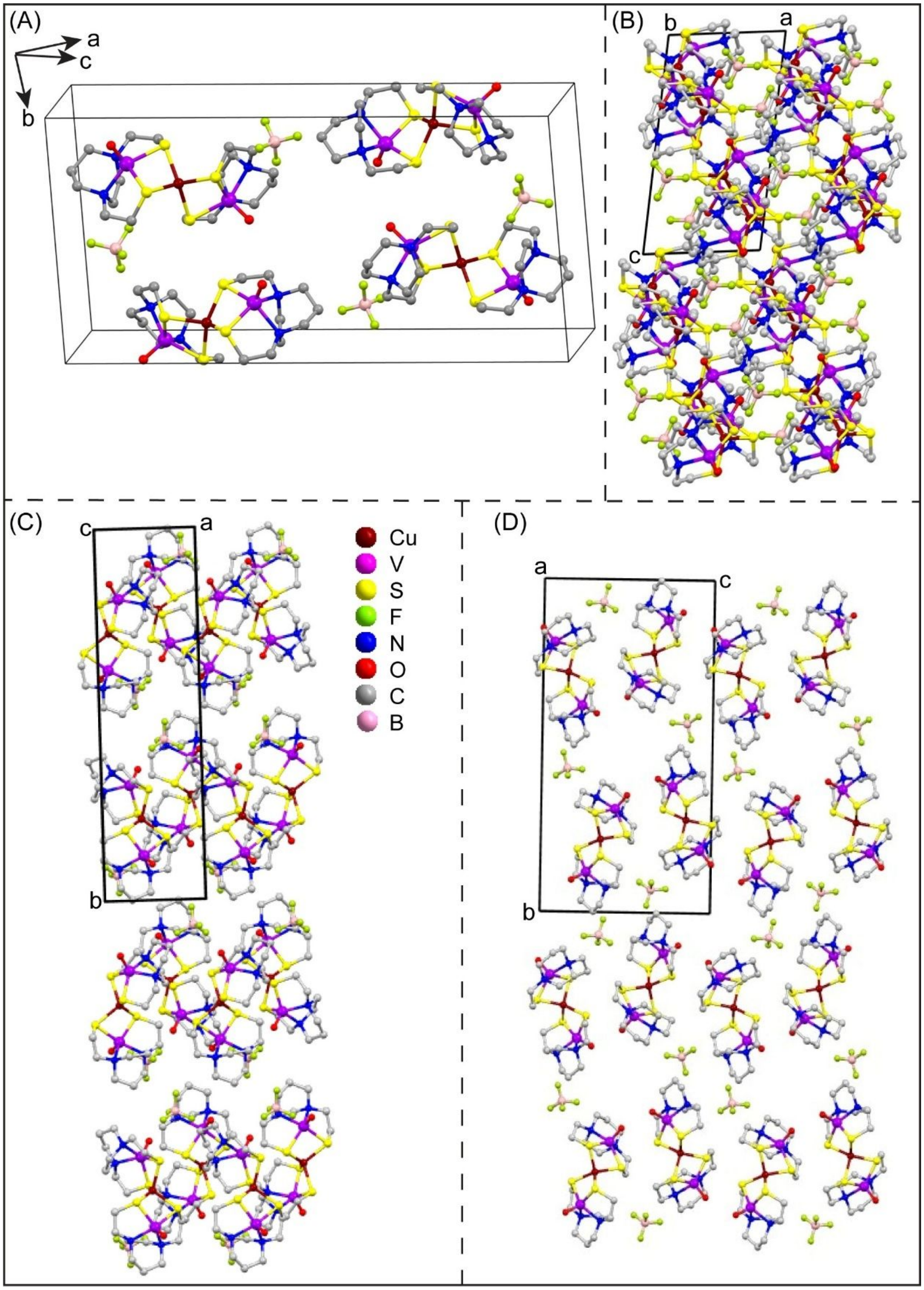

Figure S11. Extended packing of **[VCuV][BF$_4$]** in the (A) unit cell, (B) along ac plane, (C) along ab plane and (D) along bc plane at 100 K (H atoms have been omitted for clarity).

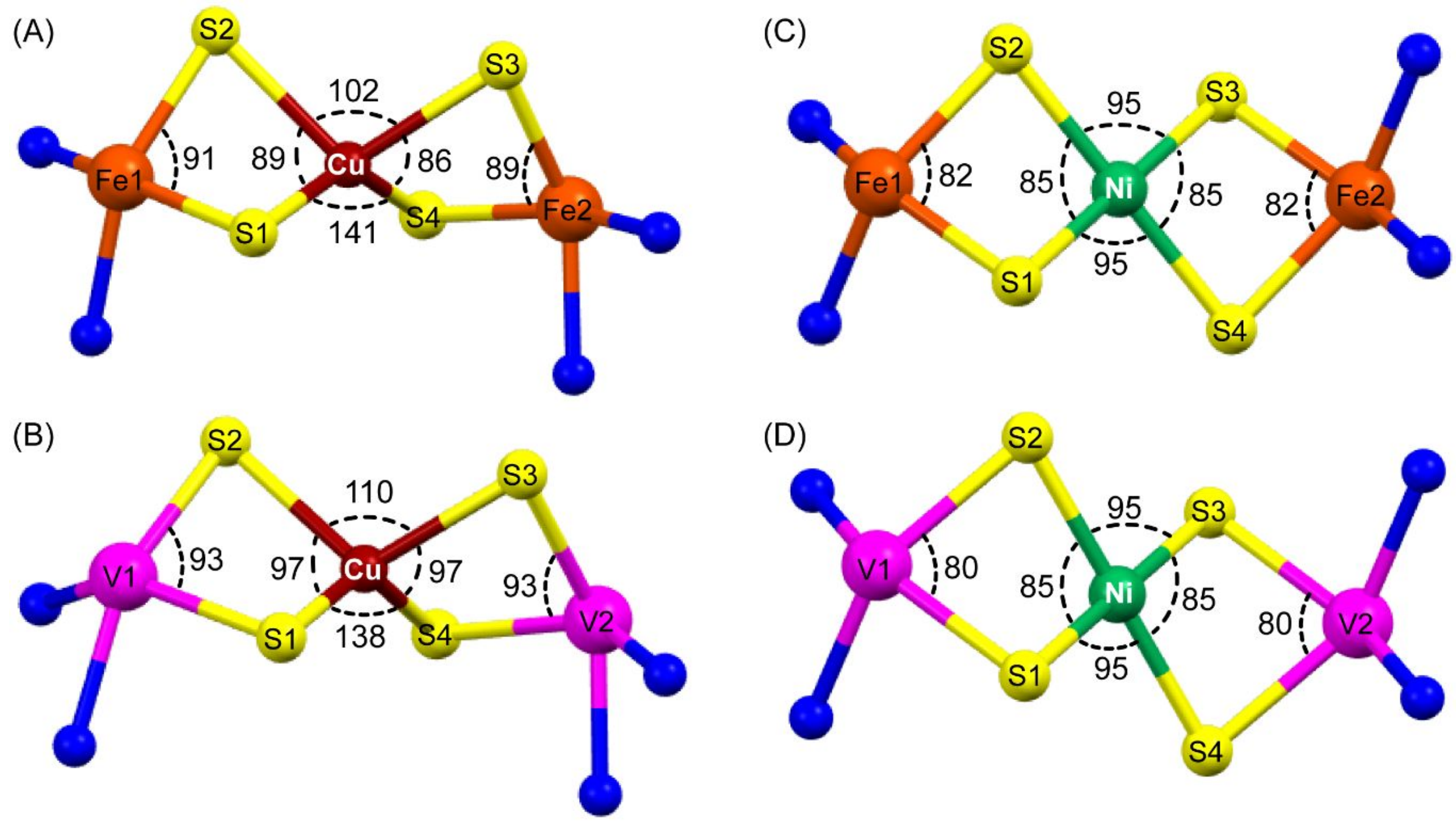

Figure S12. Selected bond angles (deg) of (A) **[FeCuFe][PF$_6$]**, (B) **[VCuV][BF$_4$]**, (C) **[FeNiFe][2BF$_4$]** and (D) **[VNiV][2BF$_4$].** Structures of [FeNiFe] and [VNiV] are reproduced from ref 13 and 15 with permission of authors and in accordance with journal policy. Available under a CC-BY NC 3.0 license.

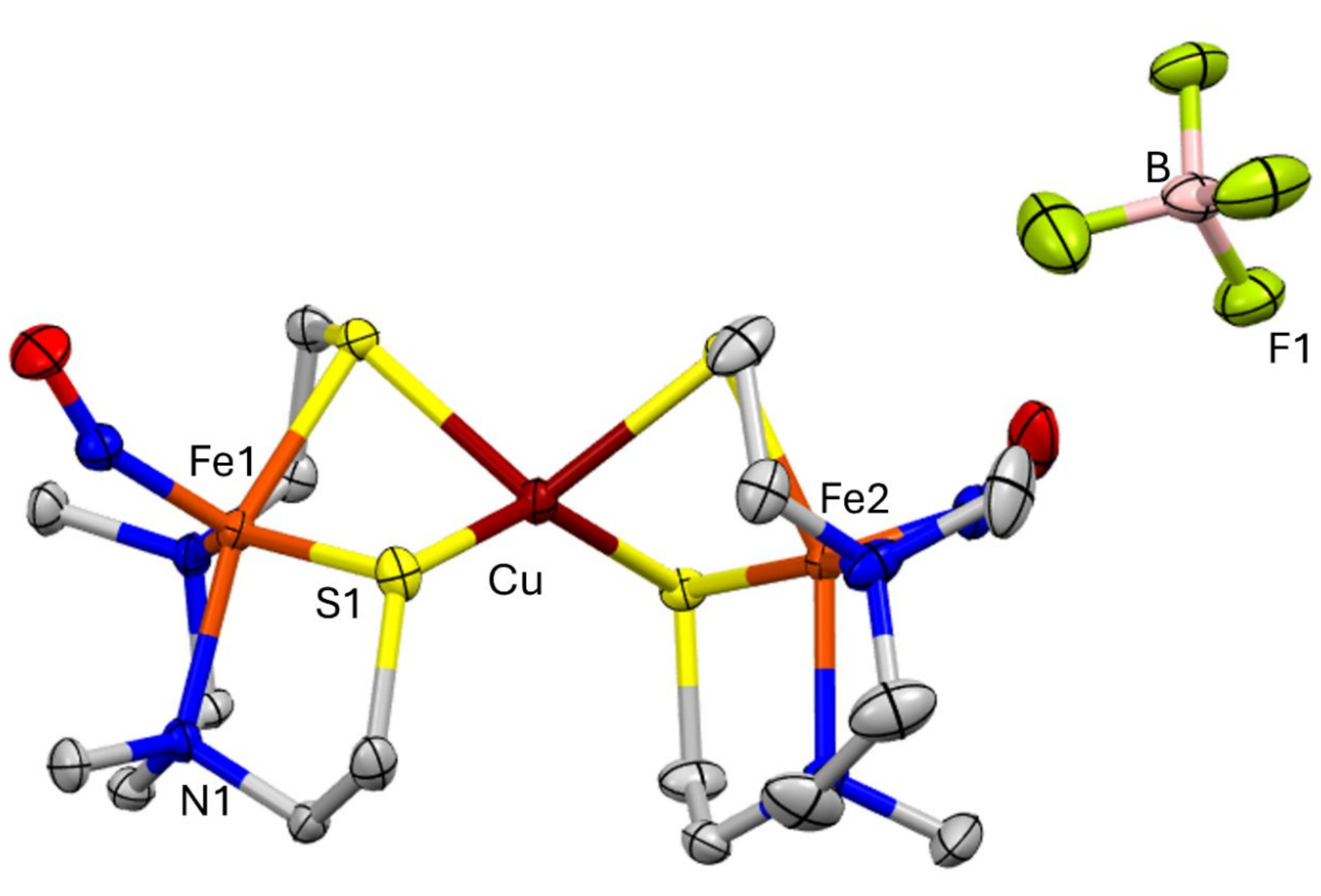

Figure S13. Abbreviated ellipsoid plots *(50% thermal contour)* at 100 K, for **[FeCuFe][BF_4].**

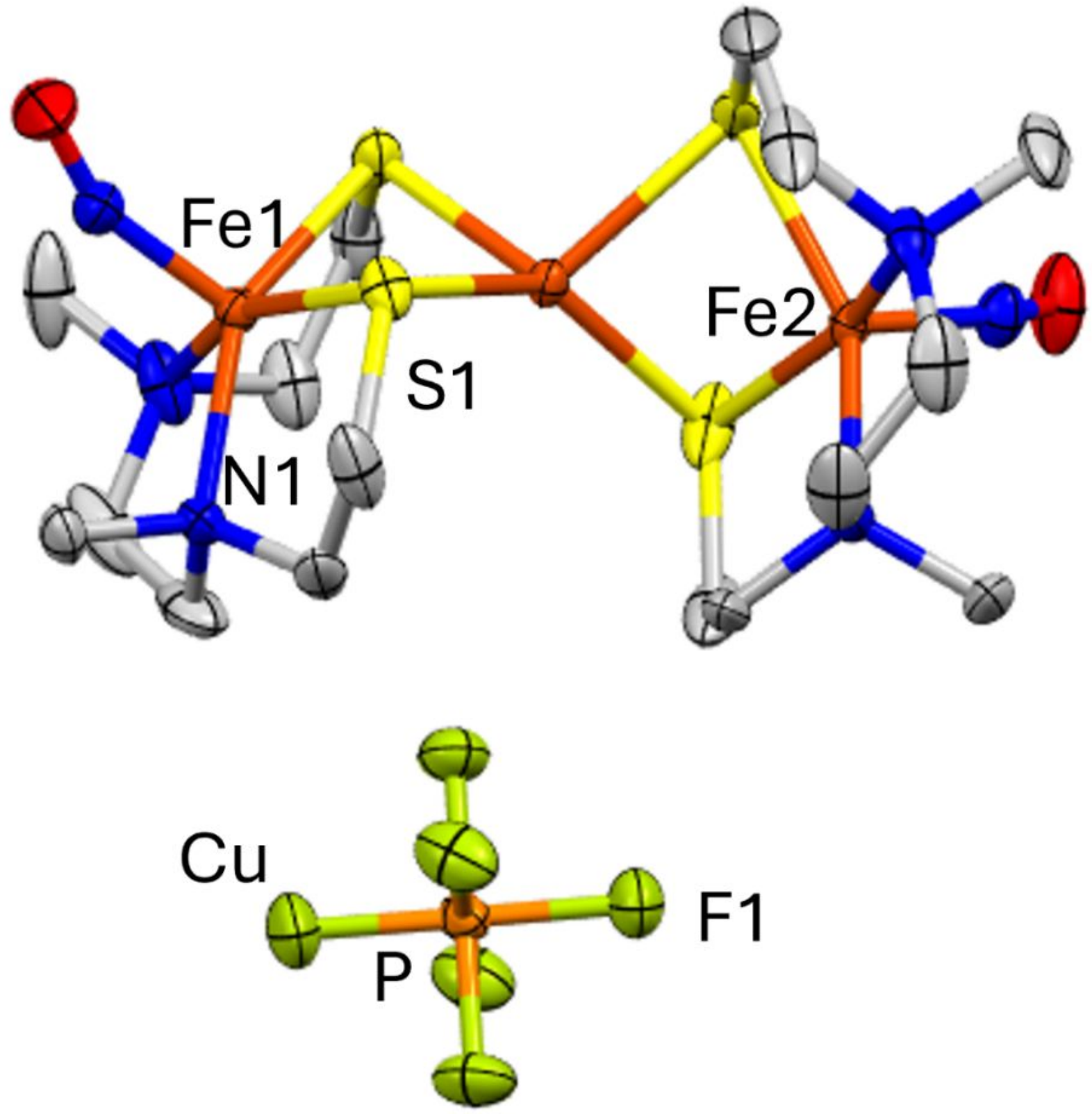

Figure S14. Abbreviated ellipsoid plots *(50% thermal contour)* at 100 K, for **[FeCuFe][PF_6].**

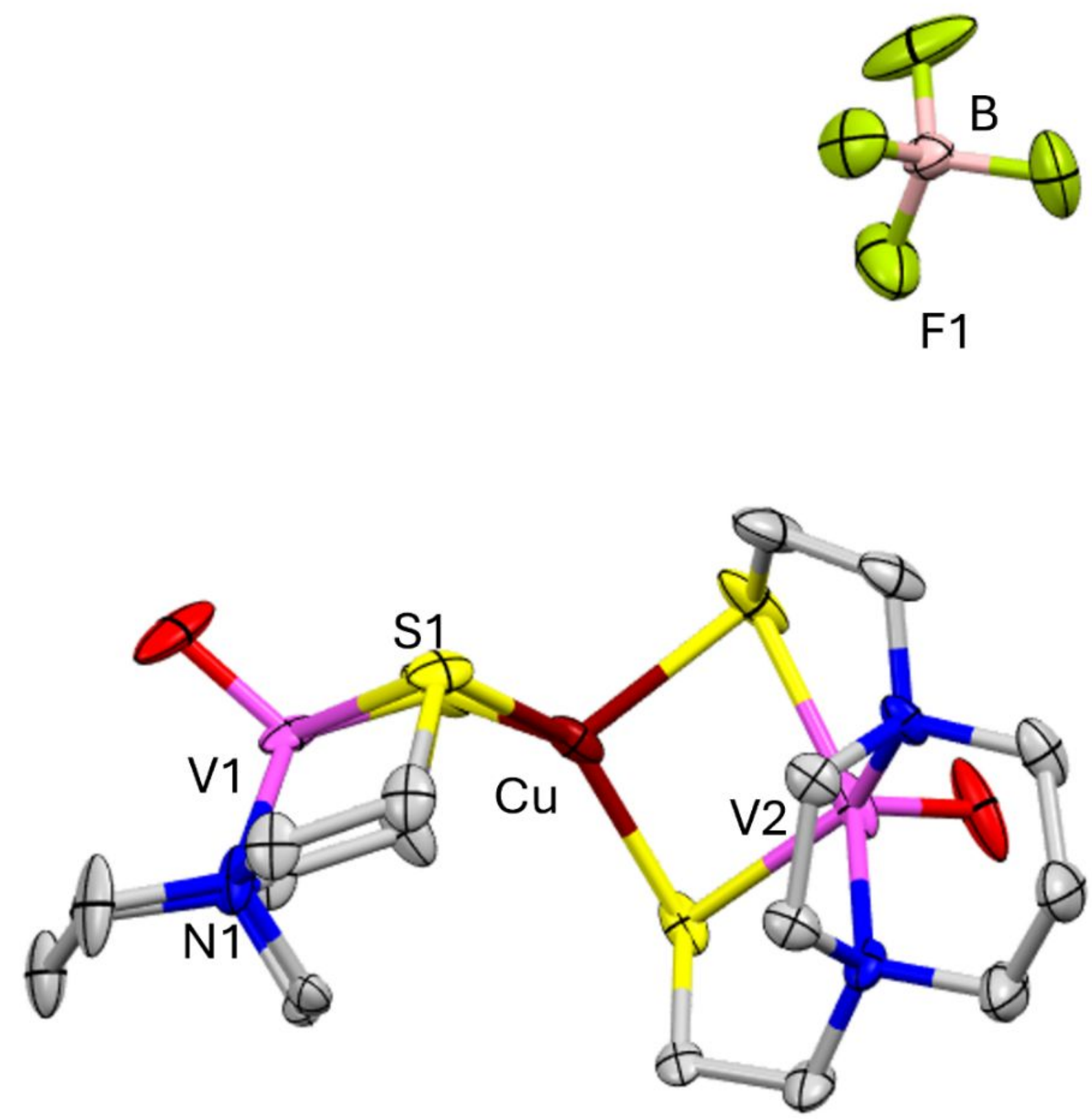

Figure S15. Abbreviated ellipsoid plots *(50% thermal contour)* at 100 K, for **[VCuV][BF_4].**

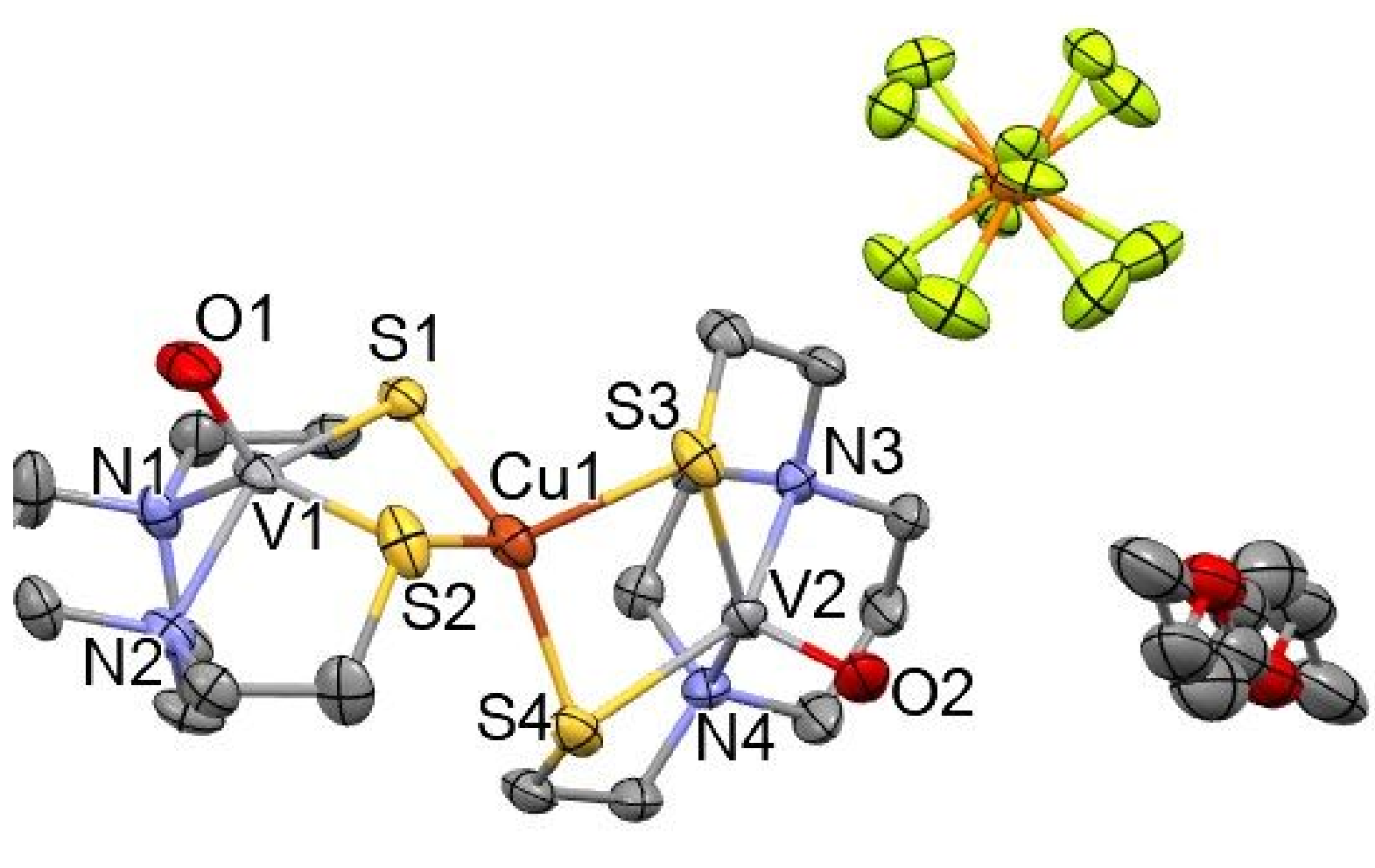

Figure S26. Abbreviated ellipsoid plots *(50% thermal contour)* at 100 K, for **[VCuV][PF_6]**.

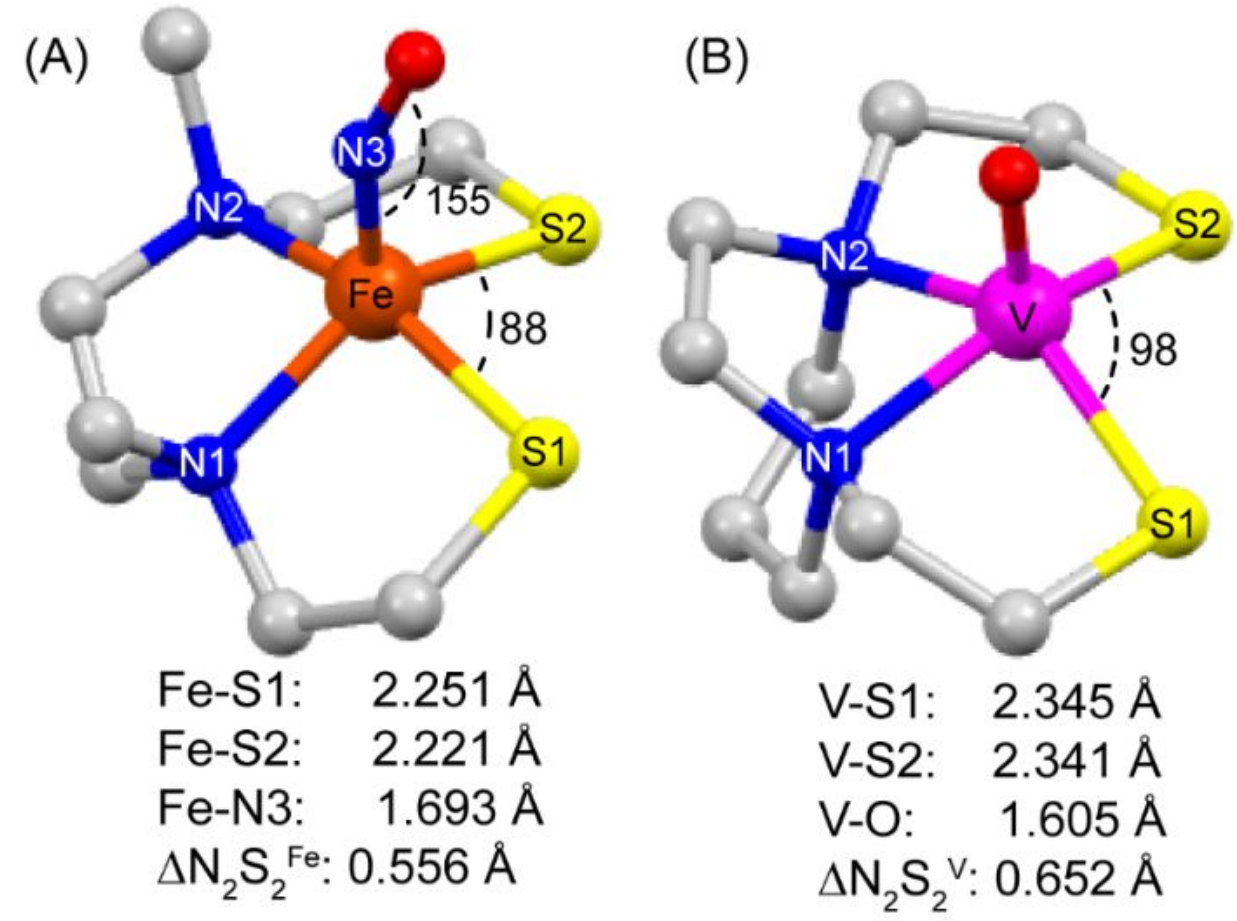

Figure S17. Molecular structures (at 100 K) of (A) parent **Fe(NO)dame** and (B) **V(O)dach** ligands showing S-M-S angles (deg). H-atoms have been omitted for clarity. Reproduced from ref 40, 45. Copyright 1977, 2011, ACS with permission of authors and in accordance with journal policy. Available under a CC-BY NC 3.0 license.

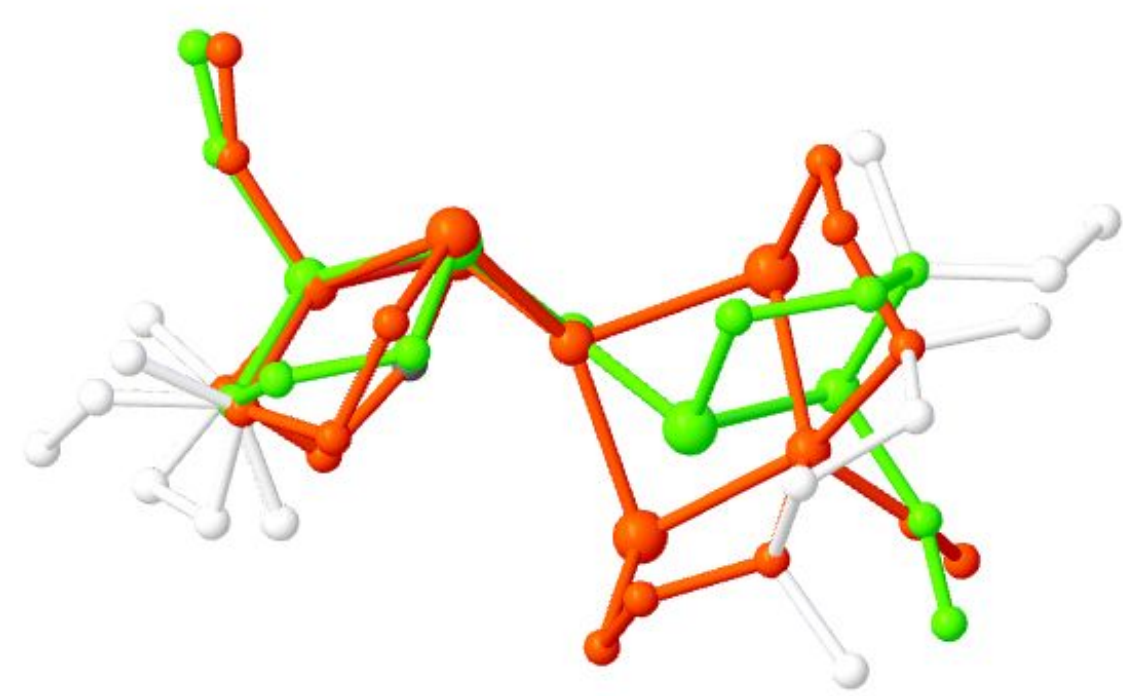

Figure S18. Overlay of Molecular structures of **[FeNiFe][BF_4]** (green) and **[FeCuFe][PF_6]** (red). Structure of [FeNiFe] is reproduced from ref 13. Copyright 2023, RSC.

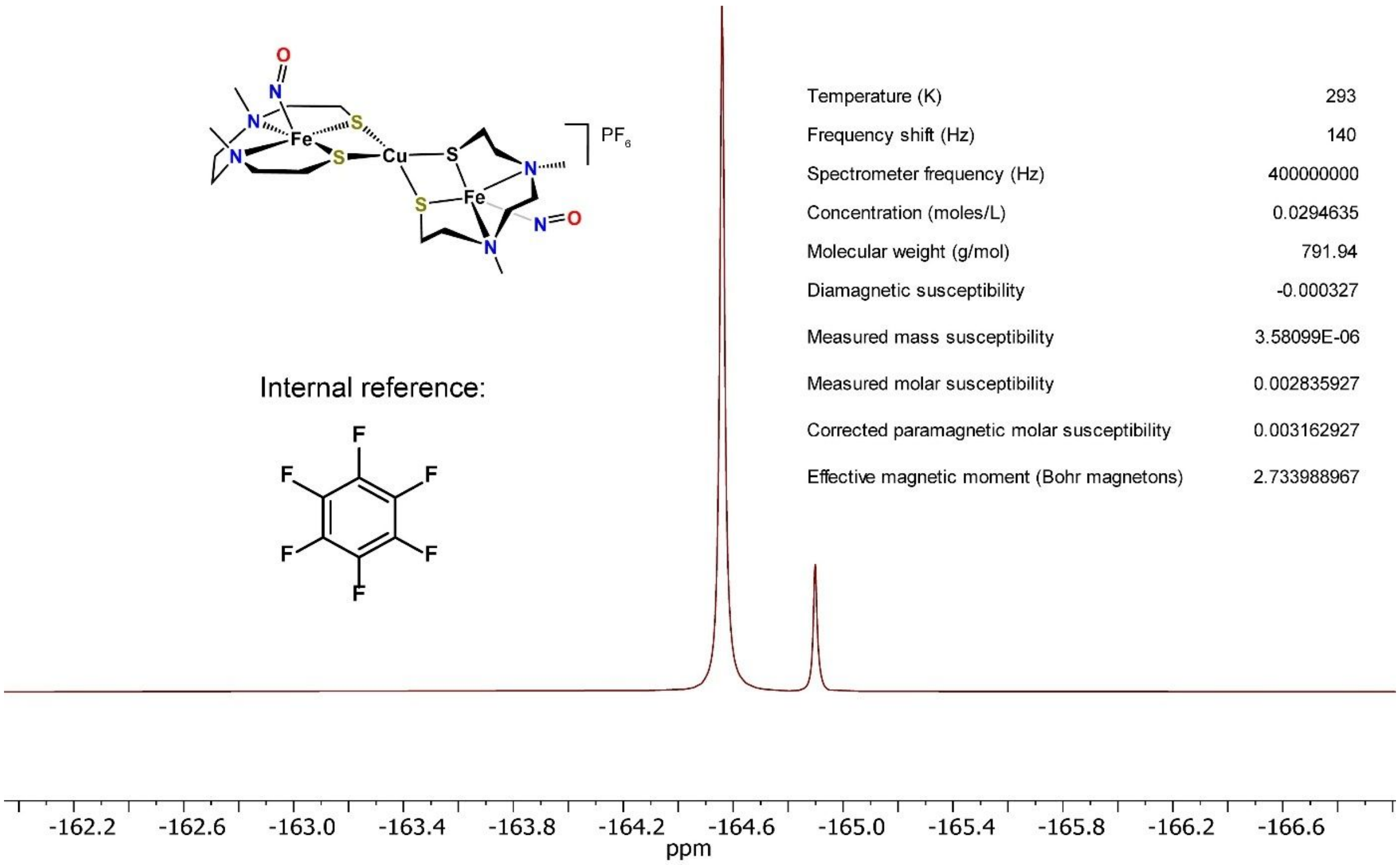

Figure S19. ^{19}F NMR spectrum of **[FeCuFe][PF$_6$]** at 293 K using a 400 MHz NMR Bruker instrument under N_2 referenced to hexafluorobenzene for Evans Method determination of magnetic susceptibility.

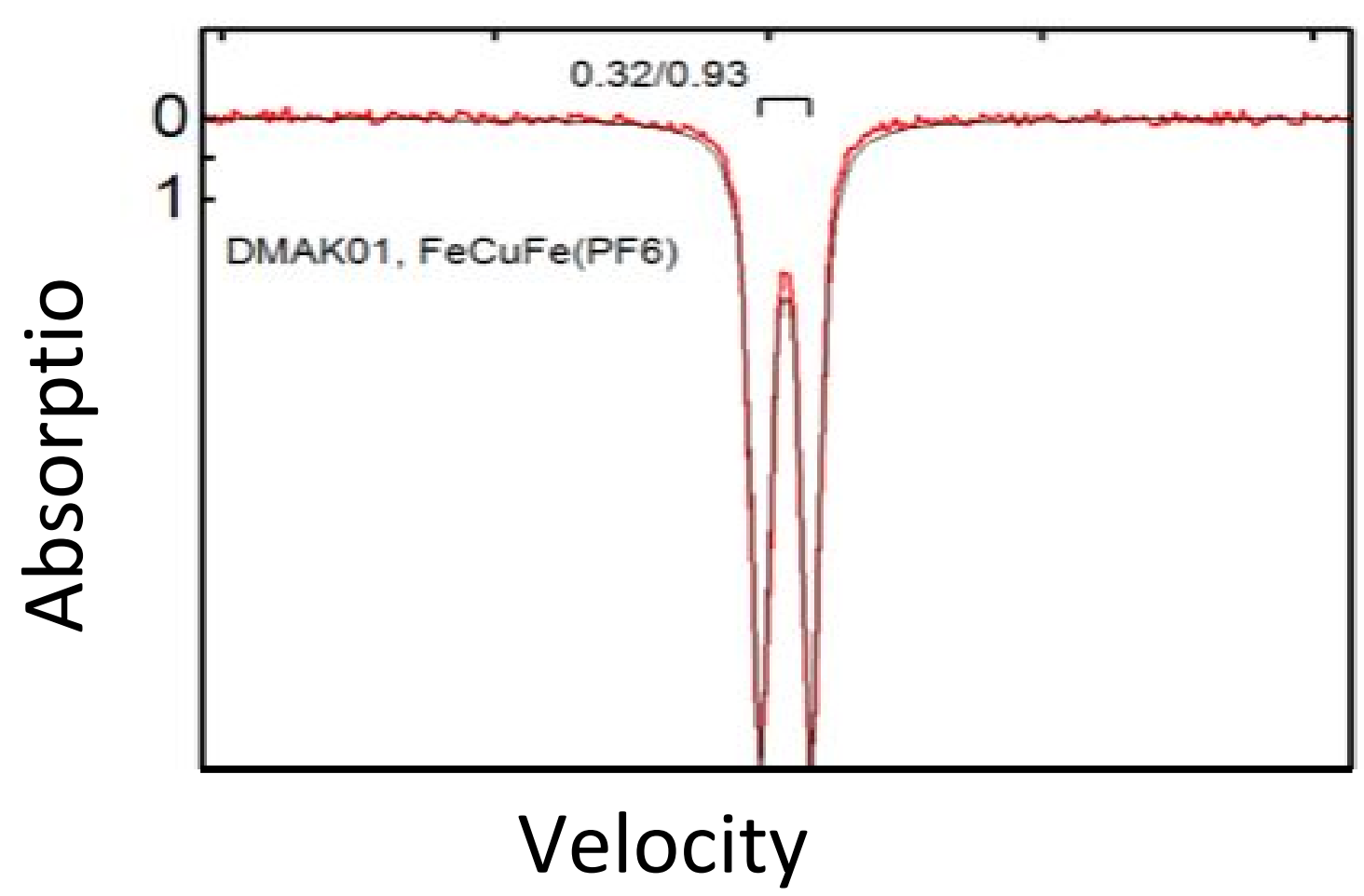

Figure S20. Zero field ^{57}Fe Mössbauer spectra of **[FeCuFe][PF$_6$]** recorded at 5 K.

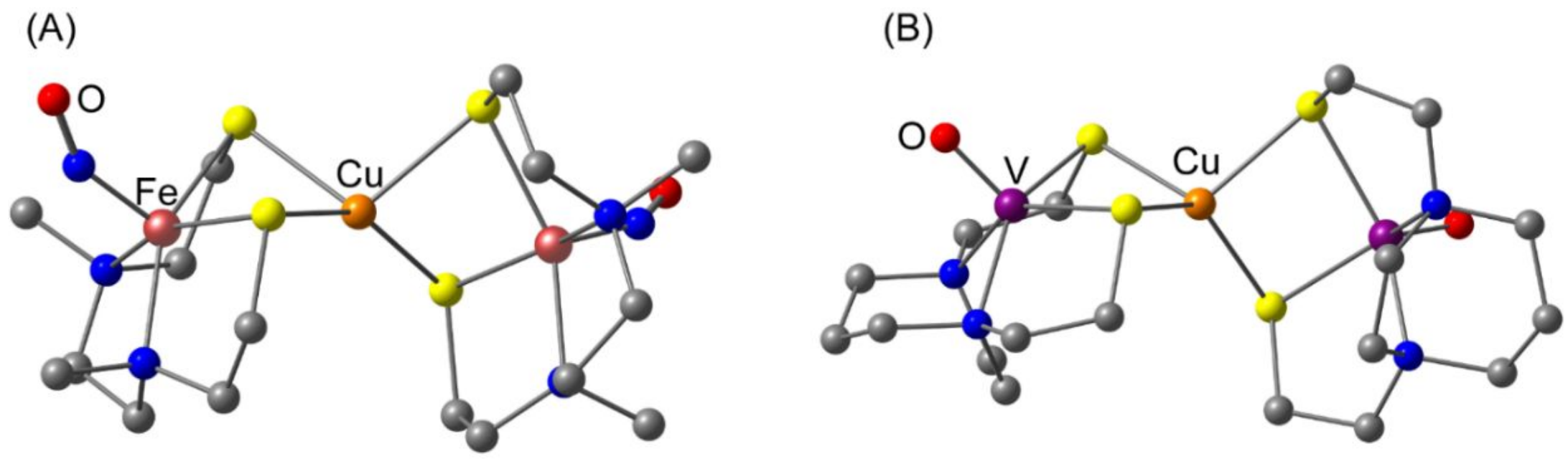

$\Delta N_2S_2^{Fe}$	0.488 [0.497]	$\Delta N_2S_2^{V}$	0.687 [0.707]
Fe-Cu	2.889 [2.893]	V-Cu	3.017 [2.997]
Fe-Fe	5.730 [5.711]	V-V	6.012 [5.911]
Fe-N-O angle	150.71 [156.10]	V-O	1.606 [1.595]
Td angle	87.90 [87.45]	Td angle	88.02 [80.35]
Interplanar angle	41.67 [36.28]	Interplanar angle	53.68 [54.78]

Figure S21. UTPSSTPSS/6-311++G**/def2TZVP optimized triplet state geometries of (A) **[FeCuFe]⁺** and (B) **[VCuV]⁺** with bond lengths in Å and angles in degrees. Parentheses contain the experimental value obtained from the respective X-ray structures. T_d angle: Angle between two tetrahedron planes ($M'S_2$) formed by copper. Interplanar angle: Angle between two intramolecular least-squares planes of N_2S_2 core.

Table S4. Relative energies (Kcal/mol) and spin-squared operator, $\langle S^2 \rangle$ of the plausible spin multiplicities of **[FeCuFe]⁺** along with coupling constant, J (cm^{-1}). All the ΔE+ZPE values are relative to triplet state.

Functional		Triplet	BS-Singlet	Quintet	Septet
**UB3LYP**	ΔE	0.00	0.02	-3.90	-7.76
	ΔE+ZPE	0.00	0.03	-4.49	-5.67
	$\langle S^2 \rangle$	3.58	2.58	7.69	13.80
	J	-6.9			
**UB97D**	ΔE	0.00	0.08	1.17	2.84
	ΔE+ZPE	0.00	-0.11	0.64	1.21
	$\langle S^2 \rangle$	2.27	1.27	6.66	13.05
	J	-26.0			
**UTPSSH**	ΔE	0.00	0.0	4.2	8.26
	ΔE+ZPE	0.00	0.03	0.98	6.24
	$\langle S^2 \rangle$	2.36	1.36	6.86	13.35
	J	0.16			
**UTPSSTPSS**	ΔE	0.00	0.00	12.27	24.26
	ΔE+ZPE	0.00	0.01	10.81	22.17
	$\langle S^2 \rangle$	2.05	1.05	6.35	12.65

J	1.95

Table S5. Relative energies (Kcal/mol) of the plausible spin multiplicities of **[VCuV]⁺** along with coupling constant, J (cm^{-1}). All the ΔE+ZPE values are relative to triplet state.

	**[FeCuFe]⁺**	**[FeCuFe]⁰**	**[FeCuFe]⁻¹**
**Calculated $\nu(NO)_{sym}$ $\nu(NO)_{asym}$**	$1753_{(493)}$, <u>$1746_{(3826)}$</u>	<u>$1728_{(1933)}$,</u> $1704_{(1655)}$	$1689_{(287)}$, $1686_{(2379)}$
**Experimental**	1694	1667, 1642	1652,* 1625

Table S6. A comparison of IR stretching vibration (cm^{-1}) calculated from UTPSSH/6-311+G**/def2TZVP optimized geometries of [FeCuFe]⁺, [FeCuFe]⁰ , and [FeCuFe]⁻¹. Harmonic frequencies (cm^{-1}), IR intensities (KM/Mole) given as subscripts.

**Functional**	**Triplet**		**BS-Singlet**
**UTPSSTPSS**	ΔE	0.00	0.01
	ΔE+ZPE	0.00	0.01
	$\langle S^2 \rangle$	2.02	1.02
	J	2.22	

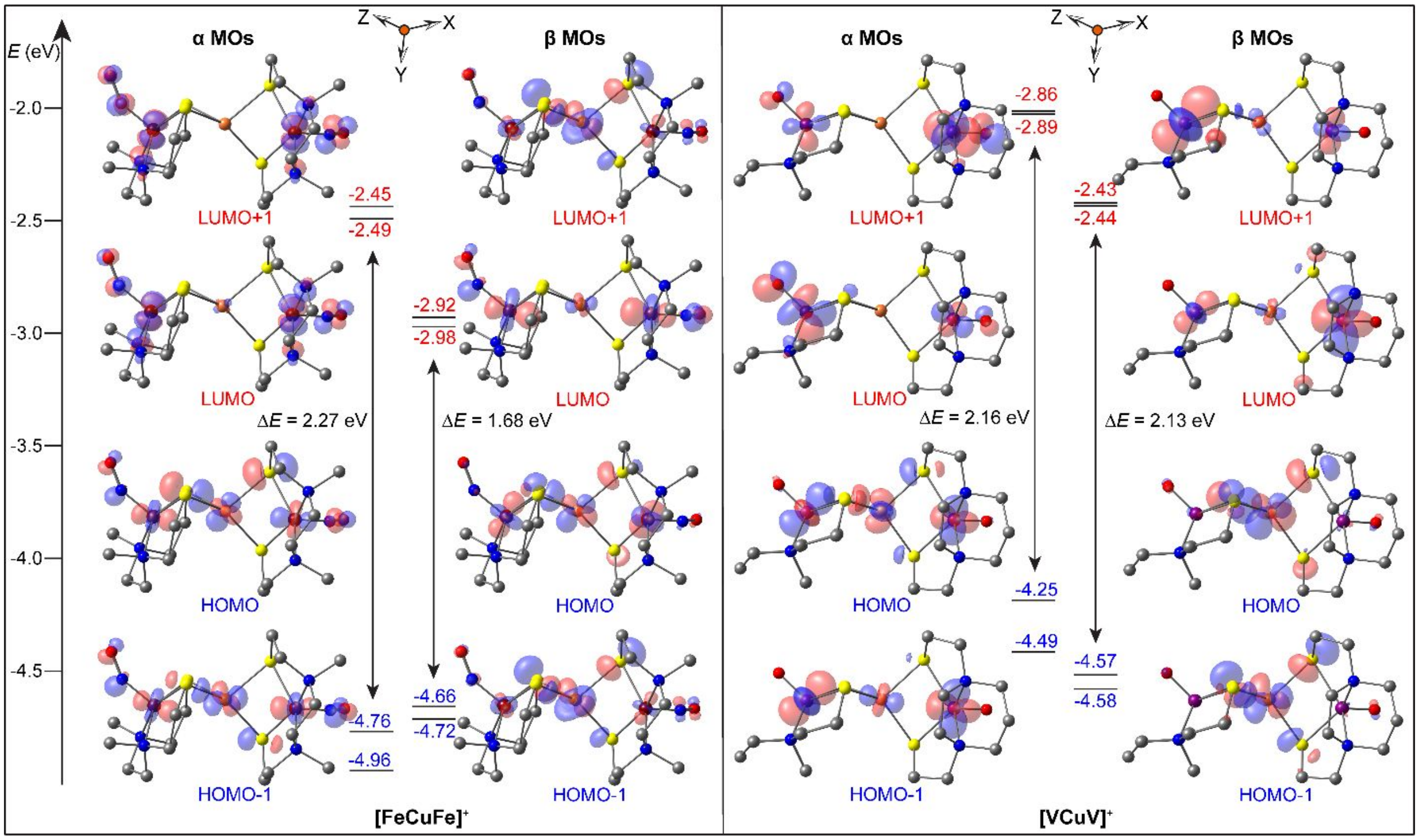

Figure S22. Frontier molecular orbitals of **[FeCuFe]**$^{+}$ and [**VCuV]**$^{+}$ (triplet). The MO surfaces are plotted at an isosurface value of 0.05 au and energies are in eV.

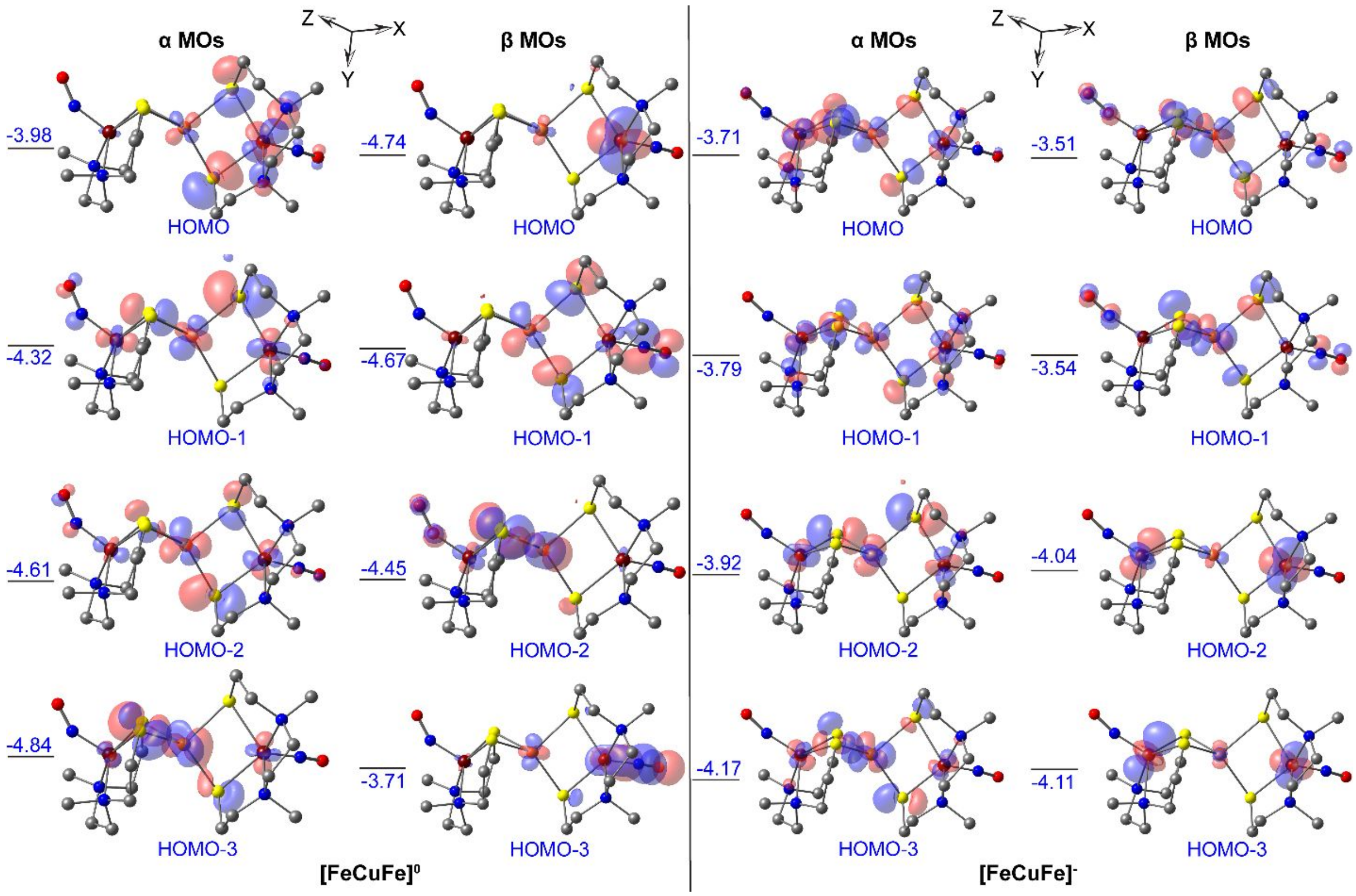

Figure S23. Selected Kohn-Sham orbitals of UTPSSH/6-311+G**/def2TZVP optimized geometry of **[FeCuFe]^0** and **[FeCuFe]$^{-1}$.** The MO surfaces are plotted at an isosurface value of 0.05 au and energies are in eV.

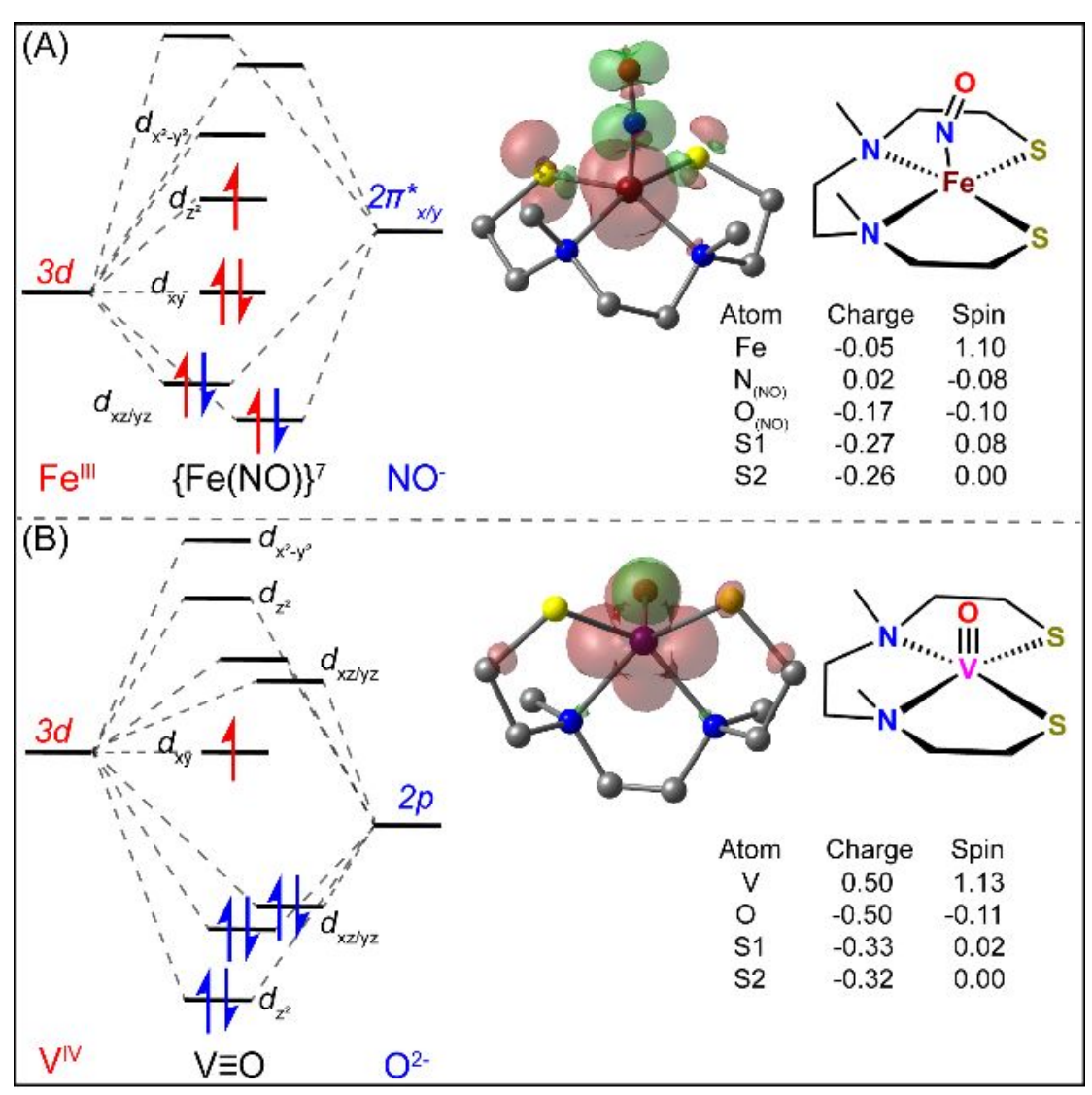

Figure S24: Qualitative molecular orbital diagrams (left) and spin density plots (isovalue = 0.003 au) (center) for (A) (NO)Fe(bme-dame) ligand (B) (O)V(bme-dame) ligand. Red arrows are metal-based electrons, while blue arrows are nitrosyl based or oxide based, N_2S_2 ligand orbitals and corresponding electron filling is omitted for clarity; Spin density plots showcasing signification spin delocalization on to the dithiolates in (NO)Fe(bme-dame) complex.

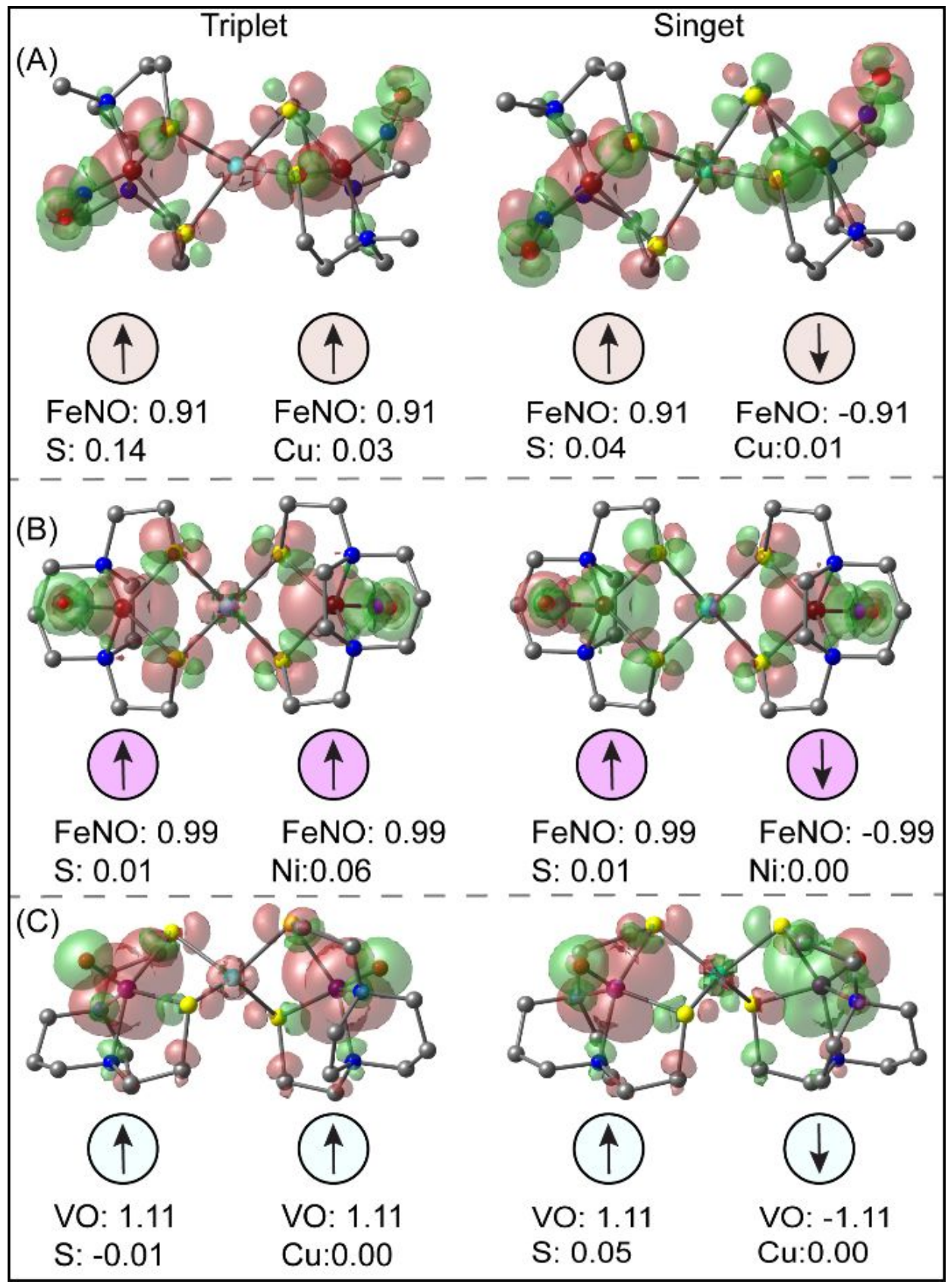

Figure S25. Coupling models and spin density distribution plots of (A) **[FeCuFe]⁺**, (B) **[FeNiFe]²⁺**, and (C) **[VCuV]⁺** calculated for open-shell singlet, and triplet states, plotted at an isosurface value of 0.001 au.

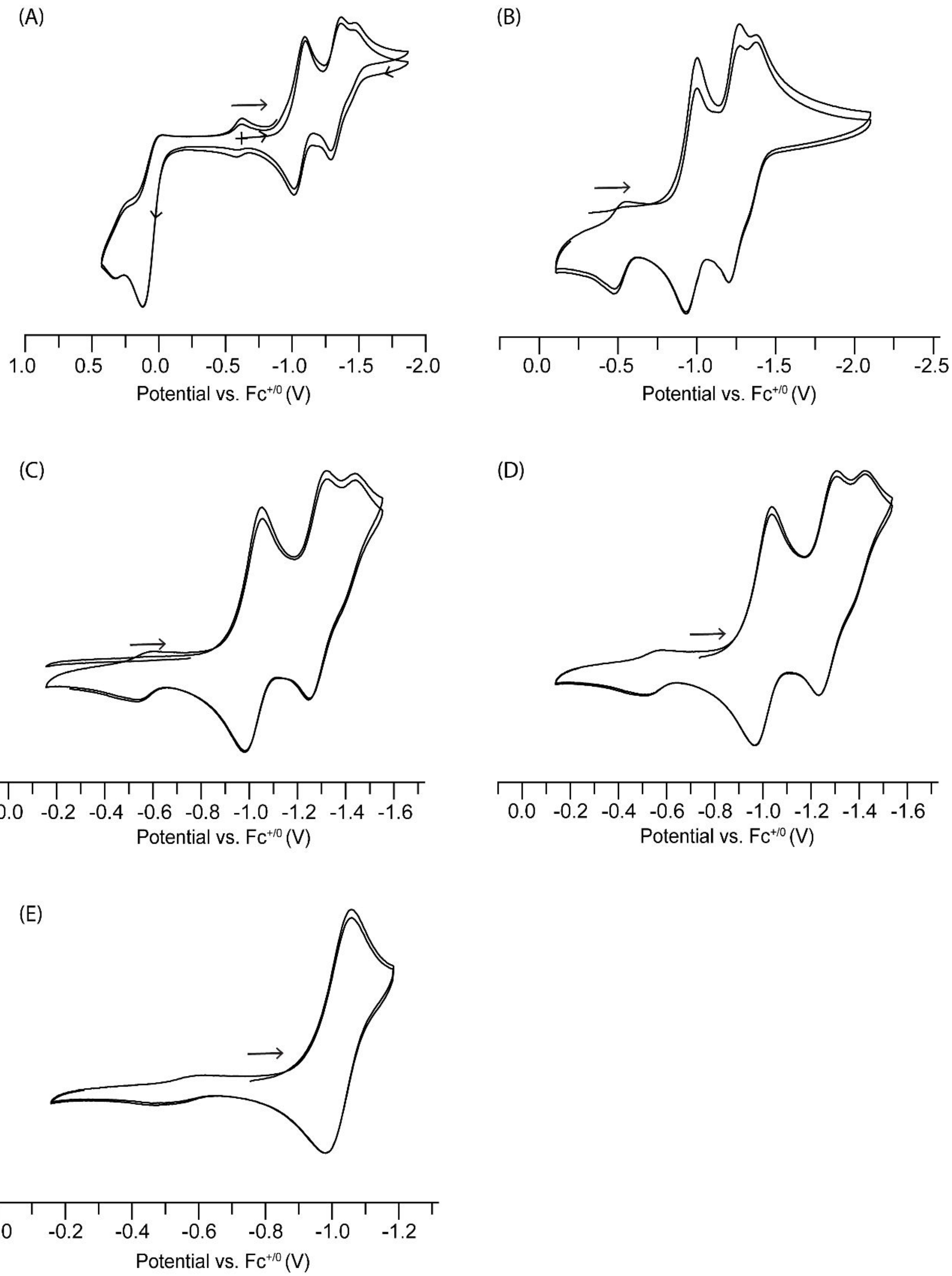

Figure S26. Cyclic voltammogram (in CH_3CN at 295 K) of **[FeCuFe][PF_6]** (scan rate 100 mV/s) with 0.1 (M) tetra(*n*-butyl) ammonium hexafluorophosphate as supporting electrolyte. CV plotted as per Polarographic (US) Convention. The reference electrode was $Ag/AgNO_3$. The voltammograms were recorded at different potential window to check the origin of a small redox event in the reverse scan at -0.5 V. This feature was not present at the starting or if we limit a window only for first reduction. This suggest that species generated at second or third reduction the responsible for this feature.

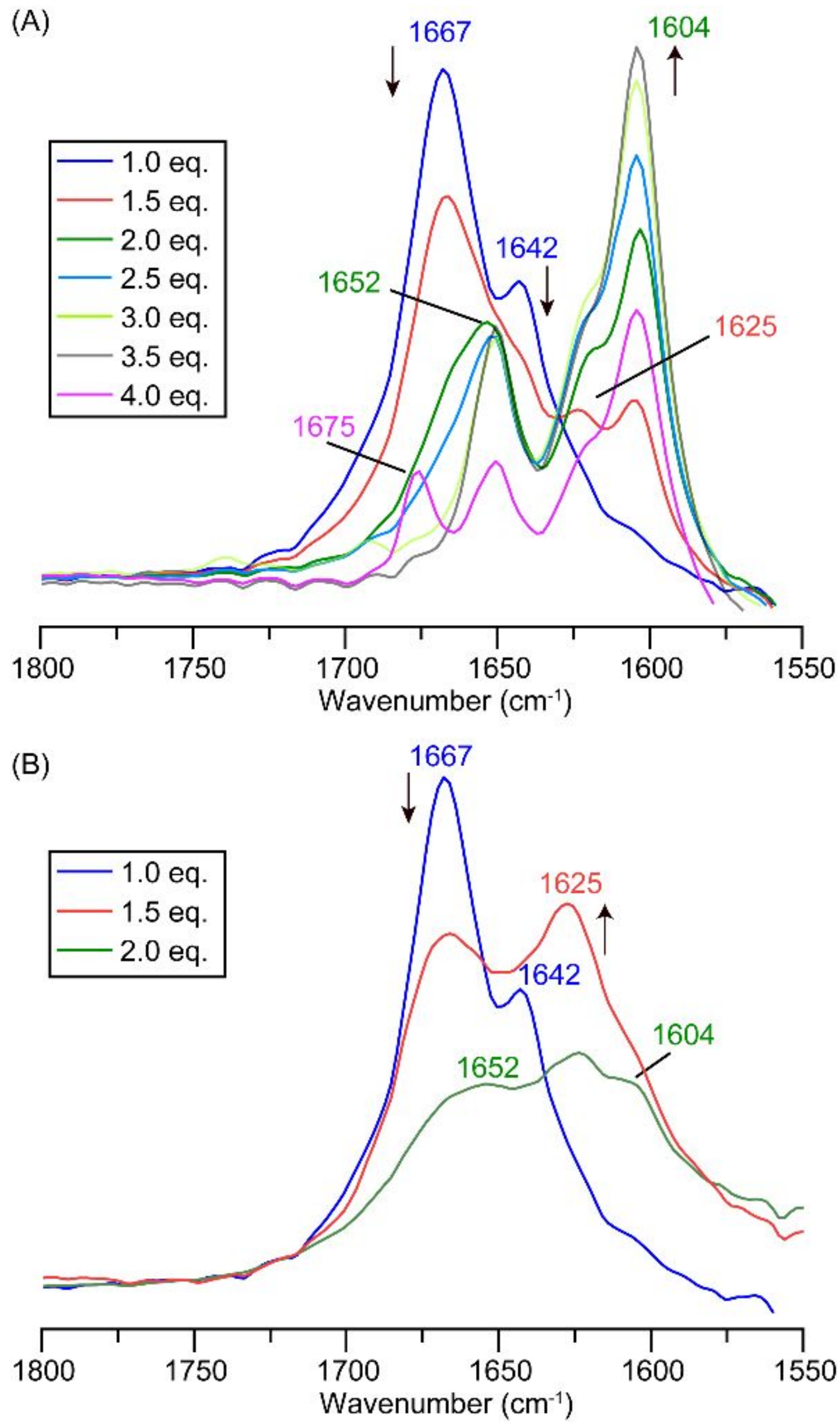

Figure S27. IR spectral changes observed (in CH_3CN at 295 K) during the reaction of a 10 mM solution of **[FeCuFe][PF_6]** with (A) 1.0-4.0 equivalent of 1 M (THF) $KHBEt_3$ (blue: 1e- oxidized complex; green: 2e- oxidized complex pink: final product); arrows indicate the direction of absorptions. (B) with $CoCp_2$.

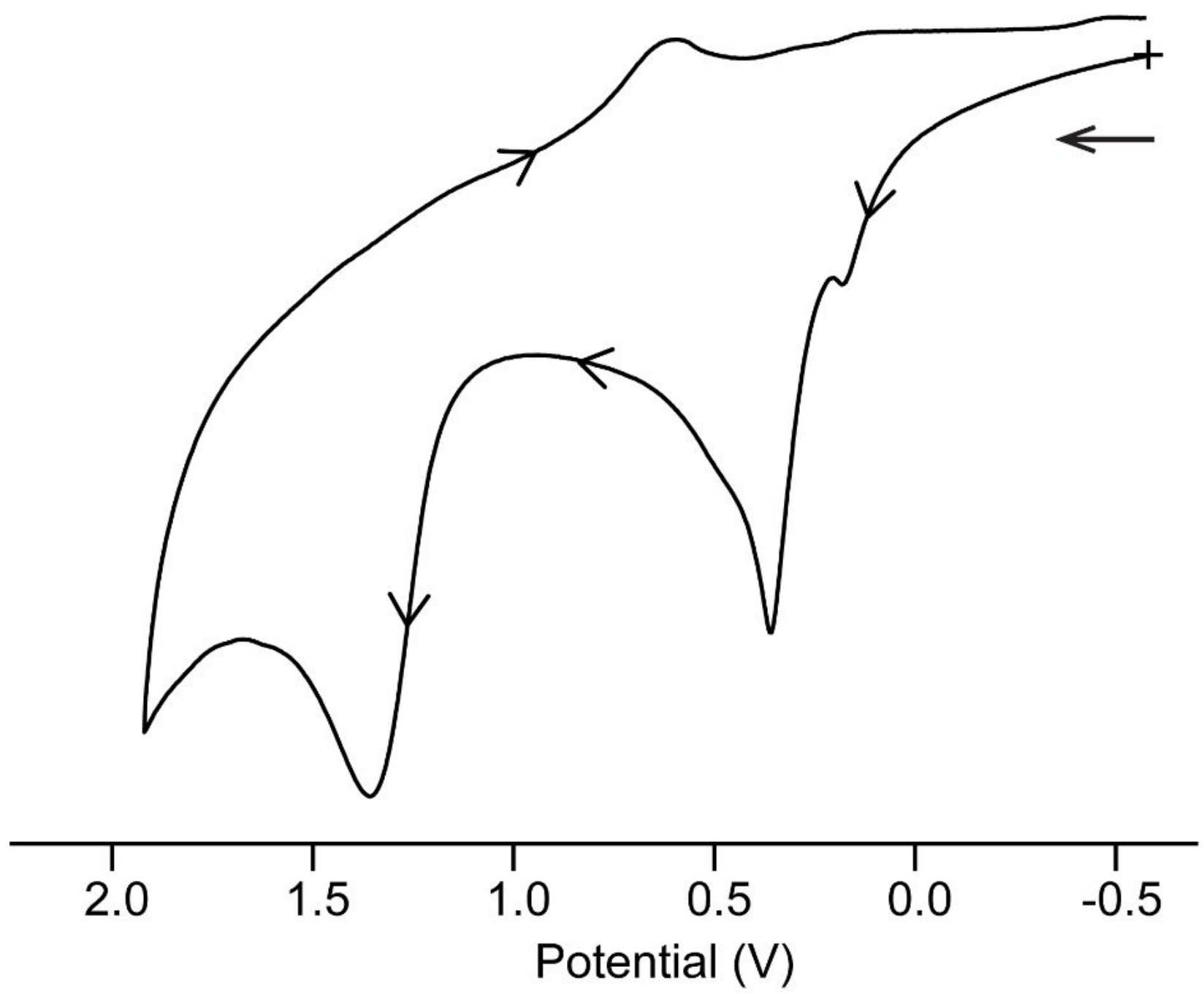

Figure S28. A portion of cyclic voltammogram (in CH_3CN at 295 K) of **[VCuV][PF$_6$]** (scan rate 100 mV/s) with 0.1 (M) tetra(*n*-butyl) ammonium hexafluorophosphate as supporting electrolyte. The reference electrode was Ag/$AgNO_3$. CV plotted as per Polarographic (US) Convention.

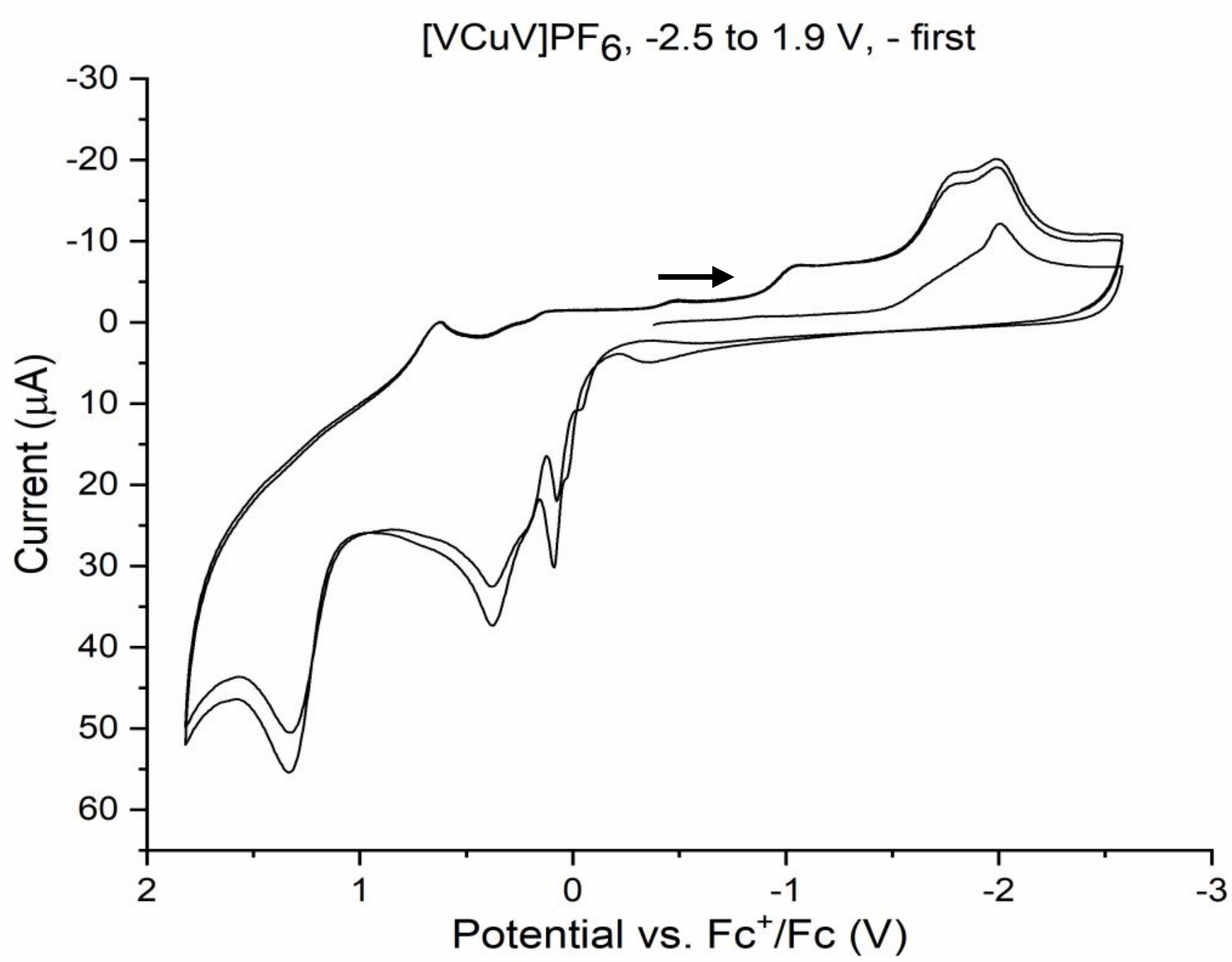

Figure S29. Cyclic voltammogram (in CH_3CN at 295 K) of **[VCuV][PF$_6$]** (scan rate 100 mV/s) with 0.1 (M) tetra(*n*-butyl) ammonium hexafluorophosphate as supporting electrolyte. The reference electrode was Ag/$AgNO_3$. CV plotted as per Polarographic (US) Convention.

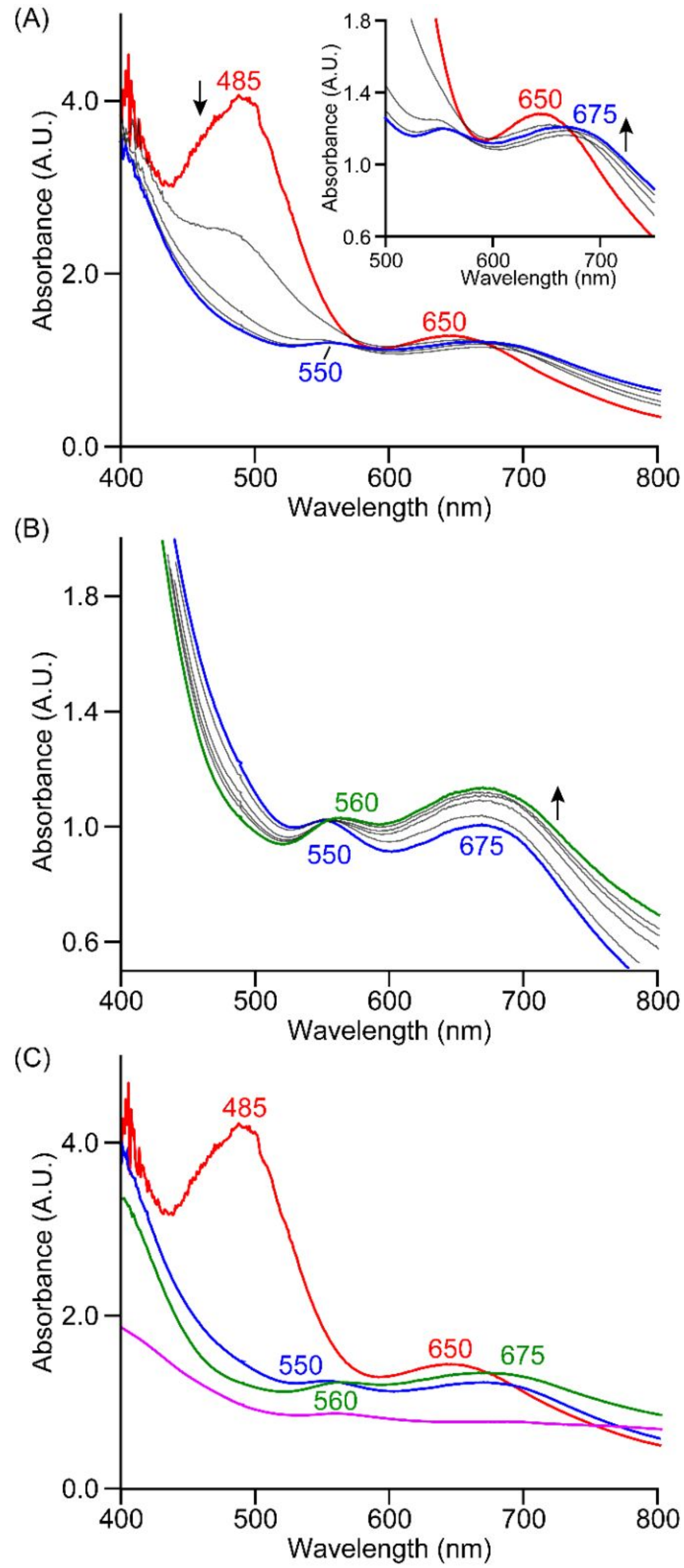

Figure S30. Electronic absorption spectral changes observed (in CH_3CN at −30 °C) during the reaction of a 0.5 mM solution of **[FeCuFe][PF_6]** with $KHBEt_3$ (A) 0-1.0 equivalent, (red: initial cationic complex; blue: 1e- reduced product); (B) 1.0-2.0 equivalent, (blue: 1e- reduced complex; green: 2e- reduced product. Inset shows the expanded region for charge transfer bands, and arrows indicate the direction of peak transition. (C) Comparison of UV-vis spectra (in CH_3CN at −30 °C) for 0.5 mM solutions of **[FeCuFe]$^+$** (red), **[FeCuFe]^0** (blue), **[FeCuFe]$^-$** (green) and **[FeCuFe]$^-$** (pink) upon warming up to room temperature.

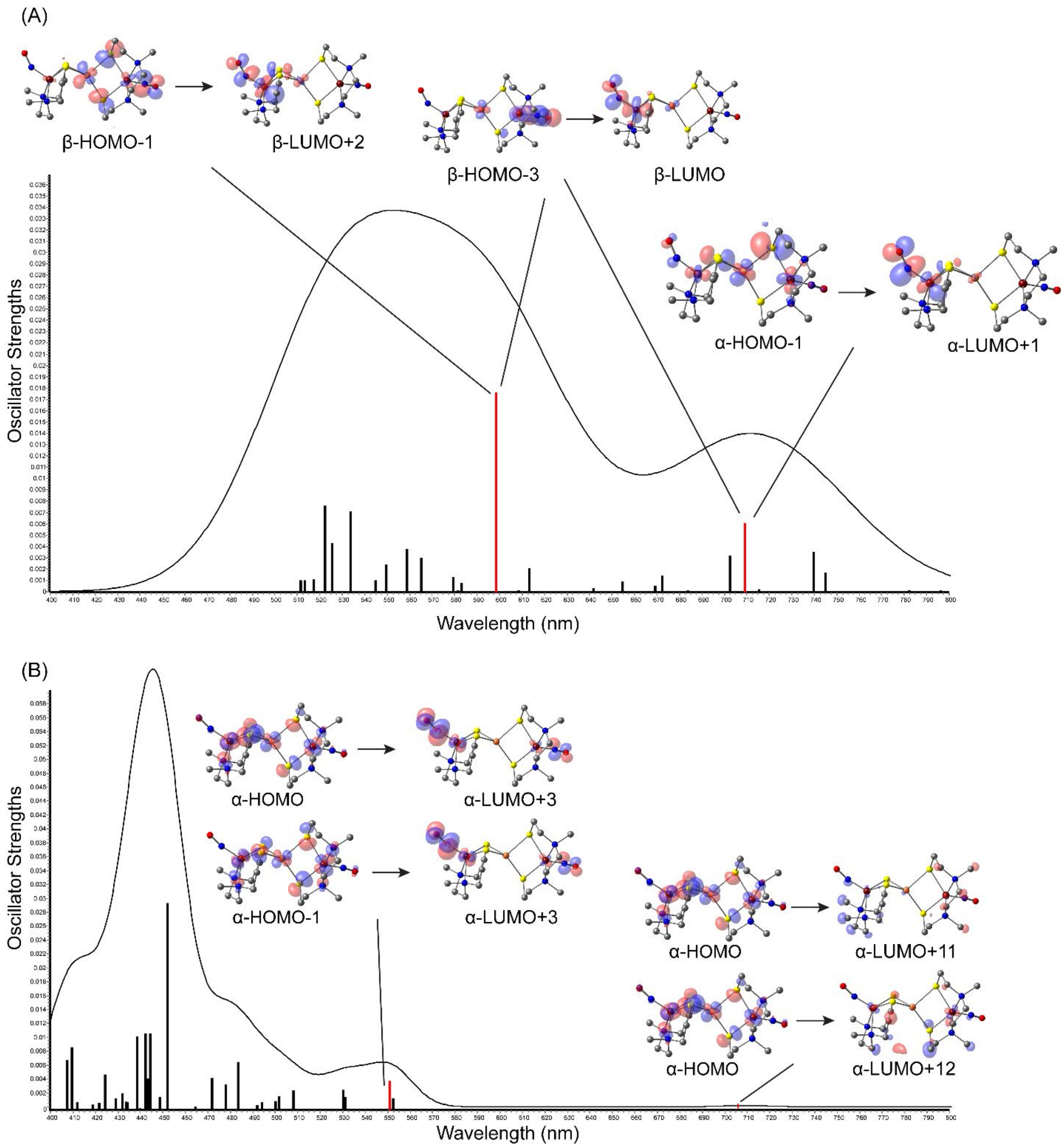

Figure S31. Absorption spectrum and oscillator strengths along with main orbital configurations, obtained from TD-DFT calculations at UTPSSH/6-311+G**/def2TZVP optimized geometry of (A) **[FeCuFe]^{0}** and (B) **[FeCuFe]$^{-1}$**.

Table S7. EPR simulation parameters for triplet (S = 1) **[M'CuM'][PF$_6$]** complexes. For brevity, only system g- and A-values are provided rather than the tensors intrinsic to each V-site. All simulations assume an intrinsic spectral linewidth (s_B) equal to the experimental modulation amplitude (0.9 mT).

[M'CuM'][PF$_6$]	(g_1, g_2, g_3)	(A_1, A_2, A_3) [MHz]	s(g_1, g_2, g_3)	J [cm^{-1}]	r (Å)
Fe-NO (S = 1)*	2.03, 2.02, 2.03	-, -, -	0.05, 0.02, 0.05	3.0	5.7
V (S = 1)	2.02, 1.94, 1.96	58, 220, 58	0.02, 0.01, 0.01	1.6	6.0

* EPR simulation parameters for **[FeCuFe][PF$_6$]** intramolecular coupling. Here, equation **D** was used to calculate the EPR spectrum from an isolated S = 2 manifold rather than a pair of coupled spins. S = 2, $g_{x,y,z}$ = (2.065, 2, 2); |D| = 0.3 cm^{-1}; E/D = 0.018; s_B = 0.9 mT.

Equation D.

$$\hat{H} = D\left(\hat{S}_z^2 - \hat{S}^2/3\right) + E\left(\hat{S}_x^2 - \hat{S}_y^2\right) + g\mu_B\vec{B}\cdot\hat{S}$$

Cartesian Coordinates of optimized geometries:

Complex [FeCuFe]$^+$ (UB3LYP/6-311+G**/def2-TZVP), solvent: CH_3CN, Charge: 1, Multiplicity: 1

29	0.000065000	-0.607190000	0.000105000
26	3.034858000	-0.244672000	0.486350000
26	-3.034754000	-0.244431000	-0.486328000
16	-1.230673000	0.150078000	-1.863408000
16	-1.940705000	-2.079998000	0.345805000
16	1.940698000	-2.079955000	-0.346363000
16	1.230980000	0.149376000	1.863731000
8	4.482624000	-1.522936000	2.589488000
7	-3.258765000	1.871511000	-0.366997000
7	3.258792000	1.871423000	0.367585000
7	4.138349000	-0.270811000	-1.309001000
7	4.170593000	-0.834816000	1.677646000
7	-4.170241000	-0.834094000	-1.678073000
7	-4.138708000	-0.271245000	1.308605000
8	-4.482180000	-1.521904000	-2.590179000
6	-4.358527000	2.348465000	-1.251280000
6	4.609387000	1.128506000	-1.566663000
6	-1.394300000	1.975315000	-2.014123000
6	3.590942000	2.138874000	-1.065147000
6	-2.573368000	-2.061390000	2.073309000
6	1.982776000	2.557298000	0.742277000
6	-3.591265000	2.138521000	1.065737000
6	-1.982736000	2.557609000	-0.741281000
6	4.358674000	2.348333000	1.251739000
6	-3.208753000	-0.718051000	2.397008000
6	5.316410000	-1.179504000	-1.249612000
6	-4.609830000	1.127986000	1.566669000
6	-5.316707000	-1.179989000	1.248720000
6	3.208254000	-0.717253000	-2.397420000
6	1.394599000	1.974563000	2.015027000
6	2.572896000	-2.060676000	-2.074043000
1	-4.121627000	2.122191000	-2.289951000
1	-5.291138000	1.844357000	-1.002513000
1	-4.492219000	3.431247000	-1.137309000
1	5.562562000	1.257272000	-1.051156000
1	4.800620000	1.278006000	-2.636069000
1	-0.405199000	2.402952000	-2.195502000
1	-2.009866000	2.194952000	-2.889624000
1	2.664597000	2.063823000	-1.639187000
1	3.974308000	3.159016000	-1.194245000
1	-3.280018000	-2.884757000	2.197840000
1	-1.736667000	-2.239667000	2.752475000
1	2.157964000	3.637539000	0.842534000
1	1.278969000	2.401663000	-0.078188000
1	-2.665070000	2.063261000	1.639990000
1	-3.974658000	3.158625000	1.195063000
1	-2.157999000	3.637866000	-0.841227000
1	-1.279044000	2.401783000	0.079248000
1	4.122101000	2.121676000	2.290400000
1	5.291328000	1.844507000	1.002572000
1	4.492112000	3.431180000	1.138085000
1	-2.426964000	0.037799000	2.498561000
1	-3.762145000	-0.764274000	3.345760000
1	5.994818000	-0.837913000	-0.468092000
1	4.995007000	-2.191588000	-1.009177000
1	5.842380000	-1.183440000	-2.211988000
1	-5.562890000	1.256935000	1.050996000
1	-4.801297000	1.277120000	2.636085000
1	-5.995014000	-0.838202000	0.467200000
1	-4.995202000	-2.191979000	1.008024000
1	-5.842836000	-1.184266000	2.211007000
1	2.426447000	0.038622000	-2.498630000
1	3.761509000	-0.763189000	-3.346267000
1	0.405520000	2.402109000	2.196729000
1	2.010321000	2.193938000	2.890485000
1	3.279484000	-2.884020000	-2.199083000
1	1.736006000	-2.238661000	-2.753051000

Complex [FeCuFe]$^+$ (UB3LYP/6-311+G**/def2-TZVP), solvent: CH_3CN, Charge: 1, Multiplicity: 3

29	0.000751000	-0.604379000	-0.000014000
26	-3.033717000	-0.243697000	-0.487010000
26	3.034200000	-0.244701000	0.486122000
16	1.230611000	0.149870000	1.863845000
16	1.940661000	-2.080795000	-0.346735000
16	-1.939732000	-2.081009000	0.342016000
16	-1.229144000	0.156741000	-1.861359000
8	-4.475183000	-1.516948000	-2.597524000
7	3.258891000	1.870728000	0.367423000
7	-3.261005000	1.871712000	-0.362791000
7	-4.139743000	-0.276598000	1.306464000
7	-4.166856000	-0.832496000	-1.681753000
7	4.169049000	-0.835736000	1.678091000
7	4.138680000	-0.271617000	-1.308469000
8	4.479112000	-1.522690000	2.591402000
6	4.358082000	2.346927000	1.252867000
6	-4.614491000	1.121032000	1.566475000
6	1.393205000	1.975341000	2.013241000

6	-3.597165000	2.134877000	1.069895000
6	2.572869000	-2.060724000	-2.074450000
6	-1.984907000	2.560282000	-0.732117000
6	3.592702000	2.138342000	-1.064931000
6	1.982682000	2.557072000	0.740619000
6	-4.359357000	2.349357000	-1.248424000
6	3.208500000	-0.717270000	-2.397207000
6	-5.315618000	-1.187889000	1.243315000
6	4.610904000	1.127401000	-1.565681000
6	5.316075000	-1.181169000	-1.248847000
6	-3.209886000	-0.723459000	2.395049000
6	-1.392962000	1.982592000	-2.005481000
6	-2.572199000	-2.065338000	2.069672000
1	4.120264000	2.120066000	2.291200000
1	5.290721000	1.842695000	1.004441000
1	4.492103000	3.429731000	1.139608000
1	-5.566661000	1.248868000	1.048864000
1	-4.808680000	1.267536000	2.635761000
1	0.403578000	2.402431000	2.193022000
1	2.007661000	2.196087000	2.889237000
1	-2.671952000	2.060492000	1.645890000
1	-3.983107000	3.153825000	1.200578000
1	3.279339000	-2.884094000	-2.199925000
1	1.735976000	-2.238219000	-2.753586000
1	-2.161000000	3.640686000	-0.829257000
1	-1.283474000	2.402797000	0.090009000
1	2.666879000	2.064008000	-1.639893000
1	3.976847000	3.158273000	-1.193309000
1	2.157909000	3.637370000	0.840343000
1	1.279977000	2.400968000	-0.080715000
1	-4.120344000	2.124859000	-2.286996000
1	-5.292083000	1.844243000	-1.002107000
1	-4.493966000	3.431863000	-1.132990000
1	2.426861000	0.038838000	-2.497942000
1	3.761758000	-0.762808000	-3.346064000
1	-5.993700000	-0.845945000	0.461650000
1	-4.991550000	-2.198624000	1.000816000
1	-5.842922000	-1.195359000	2.204942000
1	5.563741000	1.255455000	-1.049368000
1	4.803105000	1.276890000	-2.634917000
1	5.994534000	-0.840052000	-0.467162000
1	4.993887000	-2.193015000	-1.008492000
1	5.842257000	-1.185431000	-2.211103000
1	-2.429385000	0.033487000	2.498100000
1	-3.763918000	-0.771904000	3.343296000
1	-0.403433000	2.410955000	-2.182721000
1	-2.006363000	2.205366000	-2.881702000
1	-3.277519000	-2.889966000	2.193392000
1	-1.735134000	-2.243019000	2.748536000

Complex $[FeCuFe]^+$ (UB97D/6-311+G**/def2-TZVP), solvent: CH_3CN, Charge: 1, Multiplicity: 1

29	-0.032268000	-0.721016000	-0.088843000
26	-2.930173000	-0.326753000	-0.494540000
26	2.858380000	-0.315776000	0.468959000
16	1.031608000	0.059724000	1.831414000
16	1.859267000	-2.198007000	-0.321289000
16	-1.913211000	-2.225987000	0.224259000
16	-1.179302000	0.025334000	-1.963883000
8	-4.574869000	-1.549109000	-2.392333000
7	2.997066000	1.801912000	0.285363000
7	-3.010962000	1.795469000	-0.288165000
7	-3.890072000	-0.323878000	1.371547000
7	-4.128136000	-0.823087000	-1.560964000
7	3.976903000	-0.839905000	1.606580000
7	3.934306000	-0.321685000	-1.331746000
8	4.347135000	-1.569122000	2.471732000
6	4.055935000	2.328198000	1.198355000
6	-4.326597000	1.082792000	1.672807000
6	1.106605000	1.902163000	1.903512000
6	-3.282930000	2.065514000	1.163030000
6	2.393837000	-2.133030000	-2.089355000
6	-1.704259000	2.413730000	-0.683223000
6	3.362387000	2.077757000	-1.144348000
6	1.682801000	2.447655000	0.604739000
6	-4.111306000	2.351244000	-1.131390000
6	2.973119000	-0.757870000	-2.404997000
6	-5.084450000	-1.220206000	1.405327000
6	4.414820000	1.077547000	-1.598343000
6	5.111632000	-1.240444000	-1.294382000
6	-2.870375000	-0.782471000	2.380324000
6	-1.218885000	1.869041000	-2.018781000
6	-2.336936000	-2.165640000	2.022101000
1	3.795293000	2.080062000	2.231191000
1	5.012402000	1.853007000	0.962014000
1	4.139968000	3.421426000	1.081908000
1	-5.284647000	1.246849000	1.166473000
1	-4.491393000	1.208596000	2.754648000
1	0.092386000	2.296224000	2.055640000
1	1.710244000	2.197678000	2.771531000
1	-2.341285000	1.933233000	1.709199000
1	-3.618598000	3.104522000	1.310561000
1	3.124413000	-2.930859000	-2.275507000
1	1.515854000	-2.312823000	-2.722616000
1	-1.801893000	3.513394000	-0.703213000
1	-0.986267000	2.131278000	0.093969000
1	2.452546000	1.972236000	-1.747626000
1	3.728885000	3.110265000	-1.260495000
1	1.805001000	3.544372000	0.643203000
1	1.007838000	2.192714000	-0.220107000
1	-3.917891000	2.107711000	-2.180056000
1	-5.061468000	1.892764000	-0.842299000
1	-4.166308000	3.444902000	-1.001309000
1	2.158187000	-0.024159000	-2.436084000
1	3.496531000	-0.756270000	-3.377215000
1	-5.820556000	-0.844241000	0.686881000
1	-4.789230000	-2.231638000	1.112564000
1	-5.517471000	-1.231055000	2.418862000
1	5.344701000	1.220438000	-1.036063000
1	4.645057000	1.205605000	-2.667797000
1	5.807922000	-0.880185000	-0.529775000
1	4.779828000	-2.246785000	-1.024209000
1	5.606961000	-1.256739000	-2.278891000
1	-2.040872000	-0.065297000	2.356437000
1	-3.329366000	-0.774990000	3.384372000
1	-0.204479000	2.237796000	-2.224965000
1	-1.864982000	2.186599000	-2.847689000
1	-3.067430000	-2.952802000	2.249514000
1	-1.424146000	-2.363061000	2.598549000

Complex $[FeCuFe]^+$ (UB97D/6-311+G**/def2-TZVP), solvent: CH_3CN, Charge: 1, Multiplicity: 3

29	-0.000462000	-0.644851000	-0.002088000
26	-2.877392000	-0.244430000	-0.526279000
26	2.876378000	-0.243138000	0.527280000
16	1.068399000	0.117729000	1.914428000
16	1.889184000	-2.143885000	-0.245703000
16	-1.889833000	-2.144350000	0.248133000
16	-1.072452000	0.111711000	-1.918952000
8	-4.392940000	-1.452098000	-2.537943000
7	2.994237000	1.873628000	0.337938000
7	-2.992993000	1.872867000	-0.341263000
7	-3.924691000	-0.241802000	1.292510000
7	-4.016354000	-0.754425000	-1.650015000
7	4.011491000	-0.751664000	1.655531000
7	3.928630000	-0.245456000	-1.288761000
8	4.384335000	-1.447662000	2.546346000
6	4.061518000	2.412758000	1.233191000
6	-4.384884000	1.162622000	1.567793000
6	1.121497000	1.961197000	1.980496000
6	-3.328741000	2.150354000	1.095582000
6	2.397054000	-2.075166000	-2.021210000
6	-1.678042000	2.506015000	-0.682951000
6	3.334447000	2.147669000	-1.098552000
6	1.678831000	2.508648000	0.674324000
6	-4.062411000	2.410872000	-1.234640000
6	2.958940000	-0.695264000	-2.348424000
6	-5.112756000	-1.147237000	1.272437000
6	4.390997000	1.157919000	-1.565570000
6	5.115658000	-1.152100000	-1.263805000
6	-2.952555000	-0.690417000	2.350456000
6	-1.124686000	1.955039000	-1.989298000
6	-2.393394000	-2.071756000	2.024661000
1	3.816000000	2.168688000	2.270773000
1	5.018174000	1.943195000	0.986530000
1	4.135820000	3.505887000	1.109387000
1	-5.322757000	1.316372000	1.021997000
1	-4.594616000	1.293283000	2.641162000
1	0.102289000	2.340793000	2.138387000
1	1.728783000	2.267494000	2.842280000
1	-2.409446000	2.030062000	1.681457000
1	-3.678657000	3.187555000	1.220586000
1	3.131967000	-2.866467000	-2.218004000
1	1.511520000	-2.262858000	-2.641738000
1	-1.790430000	3.603734000	-0.719561000
1	-0.993277000	2.243687000	0.130582000
1	2.416734000	2.027127000	-1.686878000
1	3.685826000	3.184227000	-1.224692000
1	1.791929000	3.606377000	0.708565000
1	0.996281000	2.244808000	-0.140618000
1	-3.819885000	2.164540000	-2.272394000
1	-5.018685000	1.942400000	-0.984462000
1	-4.135689000	3.504303000	-1.112858000
1	2.135915000	0.029389000	-2.371582000
1	3.470054000	-0.691960000	-3.327159000
1	-5.816332000	-0.779161000	0.518275000
1	-4.795863000	-2.157080000	0.997453000
1	-5.593387000	-1.158271000	2.264316000
1	5.327414000	1.311881000	-1.017306000
1	4.604024000	1.286163000	-2.638585000
1	5.817731000	-0.783282000	-0.508610000
1	4.796961000	-2.161059000	-0.987658000
1	5.598839000	-1.165603000	-2.254416000
1	-2.128380000	0.033082000	2.369178000
1	-3.460752000	-0.684174000	3.330679000
1	-0.105682000	2.333639000	-2.150849000
1	-1.734145000	2.259635000	-2.850167000
1	-3.128873000	-2.861698000	2.224770000
1	-1.506625000	-2.259436000	2.643441000

Complex $[FeCuFe]^+$ (UTPSSTPSS/6-311+G**/def2-TZVP), solvent: CH_3CN, Charge: 1, Multiplicity: 1

29	-0.000019000	-0.619354000	-0.000458000
26	2.834152000	-0.239936000	0.458989000
26	-2.833901000	-0.238610000	-0.459622000
16	-1.110903000	0.006269000	-1.934702000
16	-1.833823000	-2.093507000	0.380708000
16	1.833733000	-2.092680000	-0.385981000
16	1.111514000	0.000940000	1.935204000
8	4.264969000	-1.637314000	2.383659000
7	-3.035897000	1.837597000	-0.498260000
7	3.036315000	1.836043000	0.503213000
7	3.845570000	-0.137161000	-1.350523000
7	3.966610000	-0.875588000	1.515902000
7	-3.965973000	-0.871409000	-1.518684000
7	-3.846274000	-0.140665000	1.349726000
8	-4.263839000	-1.630829000	-2.388637000
6	-4.207216000	2.248441000	-1.338877000
6	4.278116000	1.292429000	-1.561654000
6	-1.287403000	1.823552000	-2.271616000
6	3.259176000	2.229441000	-0.932317000
6	-2.343464000	-1.959240000	2.162825000
6	1.794510000	2.510572000	1.023708000
6	-3.258595000	2.226788000	0.938401000
6	-1.794295000	2.513714000	-1.017171000
6	4.207545000	2.244682000	1.345016000

6	-2.884790000	-0.561761000	2.432747000
6	5.053687000	-1.023087000	-1.379630000
6	-4.278193000	1.288527000	1.564815000
6	-5.054871000	-1.026059000	1.375621000
6	2.883239000	-0.554701000	-2.434155000
6	1.287708000	1.817422000	2.276517000
6	2.341940000	-1.952933000	-2.168119000
1	-4.021131000	1.961798000	-2.375864000
1	-5.108170000	1.735463000	-0.997703000
1	-4.351105000	3.336221000	-1.273768000
1	5.259853000	1.411755000	-1.093653000
1	4.393890000	1.503676000	-2.633499000
1	-0.310515000	2.225754000	-2.560611000
1	-1.968503000	1.943067000	-3.121325000
1	2.292829000	2.145709000	-1.441630000
1	3.592357000	3.275104000	-1.002102000
1	-3.080929000	-2.739729000	2.377073000
1	-1.459340000	-2.139870000	2.782876000
1	2.003305000	3.576625000	1.202280000
1	1.039055000	2.425535000	0.236112000
1	-2.292346000	2.140812000	1.447538000
1	-3.591094000	3.272452000	1.011379000
1	-2.003534000	3.580065000	-1.193400000
1	-1.038763000	2.427378000	-0.229790000
1	4.021687000	1.954788000	2.381132000
1	5.108616000	1.733053000	1.002130000
1	4.351084000	3.332704000	1.283214000
1	-2.056630000	0.155503000	2.433414000
1	-3.397925000	-0.514680000	3.406484000
1	5.756257000	-0.682483000	-0.615456000
1	4.757904000	-2.049500000	-1.153346000
1	5.529561000	-0.980415000	-2.369368000
1	-5.259762000	1.409692000	1.096941000
1	-4.394133000	1.496769000	2.637231000
1	-5.756873000	-0.682856000	0.612086000
1	-4.759509000	-2.051964000	1.146506000
1	-5.531228000	-0.986020000	2.365239000
1	2.055166000	0.162654000	-2.431917000
1	3.395649000	-0.504702000	-3.408119000
1	0.310732000	2.218766000	2.566422000
1	1.968771000	1.935067000	3.126510000
1	3.079130000	-2.732864000	-2.385364000
1	1.457293000	-2.131513000	-2.788020000

Complex $[FeCuFe]^{+}$ (UTPSSTPSS/6-311+G**/def2-TZVP), solvent: CH_3CN, Charge: 1, Multiplicity: 3

29	-0.000056000	-0.614205000	0.001771000
26	2.828008000	-0.238194000	0.458990000
26	-2.827956000	-0.240712000	-0.457786000
16	-1.108202000	-0.001363000	-1.935392000
16	-1.831641000	-2.097287000	0.390147000
16	1.831269000	-2.099175000	-0.378661000
16	1.108425000	0.009083000	1.935458000
8	4.253909000	-1.612946000	2.404243000
7	-3.032614000	1.834113000	-0.504513000
7	3.032726000	1.837008000	0.494829000
7	3.840425000	-0.144090000	-1.351023000
7	3.958997000	-0.869732000	1.519690000
7	-3.958824000	-0.877846000	-1.515284000
7	-3.840349000	-0.136409000	1.351908000
8	-4.253411000	-1.625455000	-2.396237000
6	-4.205383000	2.239772000	-1.345766000
6	4.272734000	1.284546000	-1.568828000
6	-1.286200000	1.814479000	-2.279783000
6	3.254149000	2.224051000	-0.942665000
6	-2.338377000	-1.952476000	2.172223000
6	1.792385000	2.514996000	1.014273000
6	-3.254330000	2.229476000	0.930685000
6	-1.792437000	2.509484000	-1.027780000
6	4.205476000	2.248021000	1.333521000
6	-2.878429000	-0.553328000	2.436074000
6	5.048599000	-1.030034000	-1.375591000
6	-4.272728000	1.293338000	1.562157000
6	-5.048482000	-1.022284000	1.380772000
6	2.878379000	-0.566608000	-2.432918000
6	1.286297000	1.826758000	2.270054000
6	2.337907000	-1.964157000	-2.161521000
1	-4.019754000	1.949348000	-2.381797000
1	-5.105259000	1.726814000	-1.001745000
1	-4.350837000	3.327571000	-1.284935000
1	5.254956000	1.406124000	-1.102429000
1	4.387396000	1.491181000	-2.641678000
1	-0.310097000	2.216345000	-2.571782000
1	-1.968357000	1.929560000	-3.129252000
1	2.287346000	2.137617000	-1.450608000
1	3.586839000	3.269540000	-1.017371000
1	-3.076253000	-2.731086000	2.391945000
1	-1.453617000	-2.130318000	2.792226000
1	2.002790000	3.581358000	1.188993000
1	1.036141000	2.428281000	0.227632000
1	-2.287567000	2.146162000	1.439216000
1	-3.587247000	3.275306000	0.999318000
1	-2.003114000	3.574804000	-1.208399000
1	-1.036135000	2.427336000	-0.240706000
1	4.020218000	1.963454000	2.371237000
1	5.105516000	1.733530000	0.992238000
1	4.350421000	3.335520000	1.266409000
1	-2.049827000	0.163377000	2.432788000
1	-3.390785000	-0.501452000	3.410013000
1	5.750924000	-0.685721000	-0.612838000
1	4.752664000	-2.055312000	-1.144385000
1	5.524768000	-0.992053000	-2.365391000
1	-5.255040000	1.412327000	1.095285000
1	-4.387225000	1.505766000	2.633899000
1	-5.750615000	-0.681989000	0.616036000
1	-4.752423000	-2.048735000	1.154993000
1	-5.524932000	-0.979193000	2.370227000
1	2.049966000	0.150333000	-2.433479000
1	3.390741000	-0.520132000	-3.407122000
1	0.310170000	2.230079000	2.559933000
1	1.968493000	1.946514000	3.118837000
1	3.075514000	-2.744179000	-2.377097000
1	1.453031000	-2.145047000	-2.780478000

Complex $[FeCuFe]^{+}$ (UTPSSTPSS/6-311+G**/def2-TZVP), solvent: Gas phase, Charge: 1, Multiplicity: 3

29	-0.000278000	-0.495116000	-0.000047000
26	2.810536000	-0.217374000	0.496510000
26	-2.810718000	-0.217669000	-0.496462000
16	-1.064102000	0.245284000	-1.910639000
16	-1.763657000	-2.102627000	0.146719000
16	1.763311000	-2.102479000	-0.146015000
16	1.063600000	0.246791000	1.909892000
8	4.218168000	-1.401885000	2.587336000
7	-3.068006000	1.853651000	-0.279021000
7	3.068583000	1.853659000	0.278155000
7	3.859993000	-0.364898000	-1.296296000
7	3.908213000	-0.749086000	1.652638000
7	-3.908944000	-0.749675000	-1.651945000
7	-3.859525000	-0.363793000	1.297062000
8	-4.219314000	-1.402945000	-2.586183000
6	-4.233864000	2.343690000	-1.080219000
6	4.329606000	1.013404000	-1.674020000
6	-1.293534000	2.084141000	-2.011191000
6	3.319620000	2.045542000	-1.189761000
6	-2.325423000	-2.229591000	1.910236000
6	1.833248000	2.606455000	0.685814000
6	-3.317834000	2.046217000	1.188995000
6	-1.832762000	2.605939000	-0.687943000
6	4.234007000	2.343823000	1.079939000
6	-2.914569000	-0.896781000	2.348099000
6	5.045160000	-1.274130000	-1.178753000
6	-4.328142000	1.014943000	1.674426000
6	-5.045206000	-1.272468000	1.180542000
6	2.915265000	-0.897851000	-2.347549000
6	1.293119000	2.085707000	2.009125000
6	2.325282000	-2.230215000	-1.909391000
1	-4.029315000	2.188742000	-2.141504000
1	-5.128229000	1.772008000	-0.824368000
1	-4.406788000	3.413634000	-0.886869000
1	5.304262000	1.174016000	-1.201470000
1	4.475198000	1.089430000	-2.761916000
1	-0.329433000	2.556287000	-2.236781000
1	-1.967163000	2.296167000	-2.849665000
1	2.358446000	1.901256000	-1.698268000
1	3.671547000	3.068080000	-1.400887000
1	-3.043852000	-3.052423000	1.995497000
1	-1.453299000	-2.473685000	2.526003000
1	2.053708000	3.686752000	0.726015000
1	1.087616000	2.421202000	-0.096581000
1	-2.356364000	1.901250000	1.696760000
1	-3.668797000	3.069097000	1.400071000
1	-2.053047000	3.686247000	-0.728745000
1	-1.086729000	2.421031000	0.094150000
1	4.028835000	2.189223000	2.141151000
1	5.128472000	1.771989000	0.824769000
1	4.407154000	3.413688000	0.886342000
1	-2.104471000	-0.166667000	2.461564000
1	-3.456529000	-0.984963000	3.304994000
1	5.737019000	-0.855798000	-0.443223000
1	4.716109000	-2.253953000	-0.827283000
1	5.549150000	-1.372984000	-2.151754000
1	-5.303033000	1.175877000	1.202467000
1	-4.472968000	1.091598000	2.762377000
1	-5.737241000	-0.854122000	0.445185000
1	-4.716798000	-2.252599000	0.829329000
1	-5.548751000	-1.370660000	2.153841000
1	2.105605000	-0.167357000	-2.461691000
1	3.457611000	-0.986695000	-3.304166000
1	0.328894000	2.558082000	2.233683000
1	1.966197000	2.298377000	2.847876000
1	3.043297000	-3.053441000	-1.994316000
1	1.453139000	-2.474001000	-2.525250000

Complex $[VCuV]^{+}$ (UTPSSTPSS/6-311+G**/def2-TZVP), solvent: CH_3CN, Charge: 1, Multiplicity: 1

29	0.013463000	-0.868421000	0.001200000
23	2.926156000	-0.585582000	0.735376000
23	-2.887291000	-0.490873000	-0.794343000
16	-0.857533000	0.226115000	-1.864198000
16	0.936424000	-0.034047000	1.970921000
16	1.836914000	-2.277002000	-0.564737000
16	-1.833960000	-2.305869000	0.373827000
7	3.990979000	-0.143720000	-1.082447000
7	-4.055076000	-0.261937000	1.003311000
7	3.359022000	1.518422000	0.862142000
8	3.938797000	-1.327217000	1.737002000
7	-3.358724000	1.606793000	-0.719976000
6	3.924723000	-1.266220000	-2.086388000
8	-3.831569000	-1.134747000	-1.921387000
6	-1.081931000	2.069050000	-1.694471000
6	2.986110000	2.092521000	-0.480441000
6	3.360149000	1.111169000	-1.627656000
6	-3.488372000	0.943050000	1.708387000
6	5.456532000	0.090742000	-0.770427000
6	-3.073111000	2.043054000	0.693156000
6	-2.563533000	2.412315000	-1.714463000
6	2.608093000	2.190534000	1.981998000
6	2.520636000	-1.830889000	-2.235358000
6	1.132868000	1.820711000	2.007750000
6	-4.005882000	-1.479170000	1.890631000
6	-5.507598000	-0.024943000	0.638604000
6	5.712865000	1.433736000	-0.086215000
6	4.837463000	1.720140000	1.134023000
6	-2.594384000	-2.023981000	2.048158000
6	-4.825520000	1.807605000	-1.051633000
6	-5.759052000	1.376962000	0.080912000
1	4.599305000	-2.046122000	-1.715546000
1	4.309371000	-0.902156000	-3.051657000
1	-0.590794000	2.545949000	-2.550206000
1	-0.584531000	2.408552000	-0.782015000
1	3.472764000	3.068416000	-0.605942000

1	1.904379000	2.249266000	-0.479978000
1	2.451210000	0.814536000	-2.157278000
1	4.043130000	1.573785000	-2.351901000
1	-2.605204000	0.611649000	2.260502000
1	-4.222697000	1.315735000	2.434031000
1	6.009498000	0.062101000	-1.720349000
1	5.782151000	-0.750253000	-0.149879000
1	-3.587173000	2.992903000	0.890018000
1	-1.996716000	2.216755000	0.770727000
1	-2.720078000	3.483282000	-1.511723000
1	-2.984126000	2.180521000	-2.699456000
1	2.735462000	3.280649000	1.892410000
1	3.089565000	1.859989000	2.909382000
1	1.830496000	-1.132646000	-2.720444000
1	2.561872000	-2.745235000	-2.838266000
1	0.688228000	2.185777000	2.940458000
1	0.577854000	2.255612000	1.172052000
1	-4.642051000	-2.232237000	1.412208000
1	-4.442772000	-1.222955000	2.868400000
1	-6.107672000	-0.160025000	1.550059000
1	-5.781221000	-0.803823000	-0.080191000
1	5.611711000	2.247043000	-0.815723000
1	6.759620000	1.446692000	0.243220000
1	5.091046000	1.069799000	1.977159000
1	4.982241000	2.766586000	1.439494000
1	-1.943280000	-1.355835000	2.621703000
1	-2.635957000	-2.988656000	2.566768000
1	-5.016799000	1.246114000	-1.971595000
1	-4.979737000	2.877590000	-1.252821000
1	-5.718482000	2.112279000	0.894268000
1	-6.786045000	1.404334000	-0.305015000

Complex $[VCuV]^+$ (UTPSSTPSS/6-311+G**/def2-TZVP), solvent: CH_3CN, Charge: 1, Multiplicity: 3

29	0.013463000	-0.868421000	0.001200000
23	2.926156000	-0.585582000	0.735376000
23	-2.887291000	-0.490873000	-0.794343000
16	-0.857533000	0.226115000	-1.864198000
16	0.936424000	-0.034047000	1.970921000
16	1.836914000	-2.277002000	-0.564737000
16	-1.833960000	-2.305869000	0.373827000
7	3.990979000	-0.143720000	-1.082447000
7	-4.055076000	-0.261937000	1.003311000
7	3.359022000	1.518422000	0.862142000
8	3.938797000	-1.327217000	1.737002000
7	-3.358724000	1.606793000	-0.719976000
6	3.924723000	-1.266220000	-2.086388000
8	-3.831569000	-1.134747000	-1.921387000
6	-1.081931000	2.069050000	-1.694471000
6	2.986110000	2.092521000	-0.480441000
6	3.360149000	1.111169000	-1.627656000
6	-3.488372000	0.943050000	1.708387000
6	5.456532000	0.090742000	-0.770427000
6	-3.073111000	2.043054000	0.693156000
6	-2.563533000	2.412315000	-1.714463000
6	2.608093000	2.190534000	1.981998000
6	2.520636000	-1.830889000	-2.235358000
6	1.132868000	1.820711000	2.007750000
6	-4.005882000	-1.479170000	1.890631000
6	-5.507598000	-0.024943000	0.638604000
6	5.712865000	1.433736000	-0.086215000
6	4.837463000	1.720140000	1.134023000
6	-2.594384000	-2.023981000	2.048158000
6	-4.825520000	1.807605000	-1.051633000
6	-5.759052000	1.376962000	0.080912000
1	4.599305000	-2.046122000	-1.715546000
1	4.309371000	-0.902156000	-3.051657000
1	-0.590794000	2.545949000	-2.550206000
1	-0.584531000	2.408552000	-0.782015000
1	3.472764000	3.068416000	-0.605942000
1	1.904379000	2.249266000	-0.479978000
1	2.451210000	0.814536000	-2.157278000
1	4.043130000	1.573785000	-2.351901000
1	-2.605204000	0.611649000	2.260502000
1	-4.222697000	1.315735000	2.434031000
1	6.009498000	0.062101000	-1.720349000
1	5.782151000	-0.750253000	-0.149879000
1	-3.587173000	2.992903000	0.890018000
1	-1.996716000	2.216755000	0.770727000
1	-2.720078000	3.483282000	-1.511723000
1	-2.984126000	2.180521000	-2.699456000
1	2.735462000	3.280649000	1.892410000
1	3.089565000	1.859989000	2.909382000
1	1.830496000	-1.132646000	-2.720444000
1	2.561872000	-2.745235000	-2.838266000
1	0.688228000	2.185777000	2.940458000
1	0.577854000	2.255612000	1.172052000
1	-4.642051000	-2.232237000	1.412208000
1	-4.442772000	-1.222955000	2.868400000
1	-6.107672000	-0.160025000	1.550059000
1	-5.781221000	-0.803823000	-0.080191000
1	5.611711000	2.247043000	-0.815723000
1	6.759620000	1.446692000	0.243220000
1	5.091046000	1.069799000	1.977159000
1	4.982241000	2.766586000	1.439494000
1	-1.943280000	-1.355835000	2.621703000
1	-2.635957000	-2.988656000	2.566768000
1	-5.016799000	1.246114000	-1.971595000
1	-4.979737000	2.877590000	-1.252821000
1	-5.718482000	2.112279000	0.894268000
1	-6.786045000	1.404334000	-0.305015000

Complex $[VCuV]^+$ (UTPSSTPSS/6-311+G**/def2-TZVP), solvent: gas phase, Charge: 1, Multiplicity: 1

29	-0.000167000	-0.720134000	0.000619000
23	2.892260000	-0.555004000	0.793600000
23	-2.891504000	-0.553198000	-0.794718000
16	-0.900845000	0.216946000	-1.915347000
16	0.902407000	0.212588000	1.917446000
16	1.748695000	-2.246510000	-0.412854000
16	-1.749026000	-2.247103000	0.409481000
7	3.996705000	-0.288943000	-1.054061000
7	-3.997640000	-0.290863000	1.052462000
7	3.416277000	1.549244000	0.752821000
8	3.895765000	-1.212593000	1.841182000
7	-3.416097000	1.550825000	-0.750488000
6	3.899818000	-1.485141000	-1.968646000
8	-3.893775000	-1.209130000	-1.844511000
6	-1.200801000	2.052890000	-1.841999000
6	3.059236000	2.016576000	-0.627594000
6	3.389000000	0.927159000	-1.690794000
6	-3.390907000	0.924295000	1.692019000
6	5.464121000	-0.077554000	-0.751921000
6	-3.060466000	2.015726000	0.631122000
6	-2.694473000	2.336982000	-1.810977000
6	2.695447000	2.333183000	1.815476000
6	2.476083000	-1.997105000	-2.100798000
6	1.201856000	2.048761000	1.847268000
6	-3.901297000	-1.488652000	1.965057000
6	-5.464846000	-0.079319000	0.749341000
6	5.762542000	1.307994000	-0.181354000
6	4.899769000	1.708639000	1.014477000
6	-2.477598000	-2.000633000	2.097369000
6	-4.899372000	1.710323000	-1.013232000
6	-5.763172000	1.307169000	0.181004000
1	4.526040000	-2.259361000	-1.514247000
1	4.330310000	-1.219120000	-2.947684000
1	-0.774398000	2.498401000	-2.746476000
1	-0.681464000	2.478171000	-0.978171000
1	3.569647000	2.964509000	-0.845891000
1	1.982547000	2.201309000	-0.636613000
1	2.459705000	0.606279000	-2.168565000
1	4.058885000	1.315288000	-2.470044000
1	-2.462011000	0.602820000	2.170159000
1	-4.061710000	1.310779000	2.471287000
1	6.028295000	-0.209564000	-1.686854000
1	5.752180000	-0.868246000	-0.053565000
1	-3.571386000	2.963092000	0.850688000
1	-1.983848000	2.200815000	0.641482000
1	-2.893311000	3.410947000	-1.661659000
1	-3.140969000	2.033563000	-2.763311000
1	2.893962000	3.407481000	1.668117000
1	3.142833000	2.027965000	2.766820000
1	1.828404000	-1.327776000	-2.675245000
1	2.485439000	-2.969532000	-2.602263000
1	0.776127000	2.492466000	2.752944000
1	0.681685000	2.475506000	0.984666000
1	-4.527079000	-2.262151000	1.508827000
1	-4.332593000	-1.224359000	2.944205000
1	-6.029852000	-0.213156000	1.683511000
1	-5.752071000	-0.868827000	0.049301000
1	5.682128000	2.064511000	-0.972311000
1	6.810662000	1.322759000	0.140317000
1	5.125115000	1.097508000	1.892719000
1	5.091715000	2.764321000	1.257575000
1	-1.830419000	-1.332188000	2.673411000
1	-2.487133000	-2.973911000	2.597177000
1	-5.123742000	1.100846000	-1.892871000
1	-5.091377000	2.766424000	-1.254468000
1	-5.683826000	2.062275000	0.973419000
1	-6.810983000	1.322138000	-0.141670000

Complex $[VCuV]^+$ (UTPSSTPSS/6-311+G**/def2-TZVP), solvent: gas phase, Charge: 1, Multiplicity: 3

29	-0.000167000	-0.720134000	0.000619000
23	2.892260000	-0.555004000	0.793600000
23	-2.891504000	-0.553198000	-0.794718000
16	-0.900845000	0.216946000	-1.915347000
16	0.902407000	0.212588000	1.917446000
16	1.748695000	-2.246510000	-0.412854000
16	-1.749026000	-2.247103000	0.409481000
7	3.996705000	-0.288943000	-1.054061000
7	-3.997640000	-0.290863000	1.052462000
7	3.416277000	1.549244000	0.752821000
8	3.895765000	-1.212593000	1.841182000
7	-3.416097000	1.550825000	-0.750488000
6	3.899818000	-1.485141000	-1.968646000
8	-3.893775000	-1.209130000	-1.844511000
6	-1.200801000	2.052890000	-1.841999000
6	3.059236000	2.016576000	-0.627594000
6	3.389000000	0.927159000	-1.690794000
6	-3.390907000	0.924295000	1.692019000
6	5.464121000	-0.077554000	-0.751921000
6	-3.060466000	2.015726000	0.631122000
6	-2.694473000	2.336982000	-1.810977000
6	2.695447000	2.333183000	1.815476000
6	2.476083000	-1.997105000	-2.100798000
6	1.201856000	2.048761000	1.847268000
6	-3.901297000	-1.488652000	1.965057000
6	-5.464846000	-0.079319000	0.749341000
6	5.762542000	1.307994000	-0.181354000
6	4.899769000	1.708639000	1.014477000
6	-2.477598000	-2.000633000	2.097369000
6	-4.899372000	1.710323000	-1.013232000
6	-5.763172000	1.307169000	0.181004000
1	4.526040000	-2.259361000	-1.514247000
1	4.330310000	-1.219120000	-2.947684000
1	-0.774398000	2.498401000	-2.746476000
1	-0.681464000	2.478171000	-0.978171000
1	3.569647000	2.964509000	-0.845891000
1	1.982547000	2.201309000	-0.636613000
1	2.459705000	0.606279000	-2.168565000
1	4.058885000	1.315288000	-2.470044000
1	-2.462011000	0.602820000	2.170159000
1	-4.061710000	1.310779000	2.471287000
1	6.028295000	-0.209564000	-1.686854000
1	5.752180000	-0.868246000	-0.053565000
1	-3.571386000	2.963092000	0.850688000
1	-1.983848000	2.200815000	0.641482000
1	-2.893311000	3.410947000	-1.661659000
1	-3.140969000	2.033563000	-2.763311000
1	2.893962000	3.407481000	1.668117000
1	3.142833000	2.027965000	2.766820000
1	1.828404000	-1.327776000	-2.675245000
1	2.485439000	-2.969532000	-2.602263000
1	0.776127000	2.492466000	2.752944000
1	0.681685000	2.475506000	0.984666000
1	-4.527079000	-2.262151000	1.508827000
1	-4.332593000	-1.224359000	2.944205000
1	-6.029852000	-0.213156000	1.683511000
1	-5.752071000	-0.868827000	0.049301000

1	5.682128000	2.064511000	-0.972311000
1	6.810662000	1.322759000	0.140317000
1	5.125115000	1.097508000	1.892719000
1	5.091715000	2.764321000	1.257575000
1	-1.830419000	-1.332188000	2.673411000
1	-2.487133000	-2.973911000	2.597177000
1	-5.123742000	1.100846000	-1.892871000
1	-5.091377000	2.766424000	-1.254468000
1	-5.683826000	2.062275000	0.973419000
1	-6.810983000	1.322138000	-0.141670000

Complex $[FeCuFe]^0$ (TPSSH/6-311+G**/def2-TZVP), solvent: CH_3CN, Charge: 0, Multiplicity: 4

29	-0.073824875	-0.602115533	0.033908460
26	-2.910053226	-0.358910655	-0.371553869
26	2.877442758	-0.178918337	0.744876786
16	0.820623482	0.824553914	1.636315894
16	1.740733784	-2.168032559	0.034259686
16	-1.906887380	-2.011491641	0.779437876
16	-1.285707645	-0.512584806	-1.942422490
8	-4.514343593	-2.022618826	-1.907973268
7	3.133723114	1.913990892	-0.168906270
7	-3.021767073	1.678542354	-0.819921602
7	-3.811906511	0.168923023	1.428330095
7	-4.138062416	-1.126274604	-1.226205092
7	3.979660239	-0.386840078	2.059492870
7	4.111450822	-0.636256699	-1.159900241
8	4.591838998	-0.565947098	3.085555443
6	4.113560949	2.624989977	0.684349619
6	-4.115465637	1.637293972	1.385044338
6	1.090372442	2.565647130	1.121554142
6	-3.084213428	2.339096238	0.520934245
6	2.466947980	-2.461681851	-1.626326805
6	-1.803543837	2.141282469	-1.556892337
6	3.662067777	1.775086669	-1.547924206
6	1.851789466	2.656870658	-0.195670347
6	-4.235148735	1.999244834	-1.622702555
6	3.192264215	-1.228147982	-2.160087010
6	-5.074332548	-0.586170840	1.665474582
6	4.678498562	0.641524368	-1.643964246
6	5.203286169	-1.578315083	-0.840978565
6	-2.835356249	-0.117205703	2.530807672
6	-1.444510768	1.167665690	-2.662880730
6	-2.395151073	-1.569881035	2.493764132
1	3.753254516	2.653094586	1.713464576
1	5.064712248	2.091366893	0.677771041
1	4.273235463	3.653703771	0.326065057
1	-5.118772250	1.760879466	0.972627942
1	-4.123932367	2.056925556	2.397649584
1	0.131411028	3.080572975	1.007952609
1	1.627229508	3.072027249	1.929233117
1	-2.090570487	2.241793755	0.968396805
1	-3.317254262	3.407224549	0.421762118
1	3.143183676	-3.319113796	-1.559739765
1	1.670181984	-2.729470416	-2.325797897
1	-1.966642689	3.158725649	-1.938525258
1	-0.986695061	2.157868704	-0.831425656
1	2.813414888	1.575660211	-2.207984504
1	4.125400210	2.712508417	-1.891458244
1	2.028235809	3.713124925	-0.460234054
1	1.241706646	2.205165868	-0.984095938
1	-4.152380883	1.526110191	-2.600628213
1	-5.124746375	1.607031436	-1.131456377
1	-4.329391991	3.085349366	-1.746261606
1	2.446483865	-0.470597413	-2.417951755
1	3.747825025	-1.485951247	-3.078524926
1	-5.784825507	-0.341736160	0.875348352
1	-4.870711667	-1.655878836	1.632438935
1	-5.499421590	-0.319843708	2.641113617
1	5.550433483	0.866060244	-1.022932452
1	5.034774336	0.557878384	-2.682777202
1	5.874566401	-1.111419276	-0.117520691
1	4.787564237	-2.480178570	-0.388620739
1	5.775367734	-1.854942442	-1.740238394
1	-1.961387927	0.521638346	2.372229385
1	-3.299694134	0.137596441	3.494581214
1	-0.489727687	1.447494439	-3.115811078
1	-2.196745399	1.140715444	-3.455870957
1	-3.179331372	-2.249878626	2.836078512
1	-1.526193944	-1.705584577	3.142048856

Complex $[FeCuFe]^{-1}$ (TPSSH/6-311+G**/def2-TZVP), solvent: CH_3CN, Charge: -1, Multiplicity: 5

29	-0.049213000	-0.608371000	-0.013857000
26	-3.004859000	-0.463257000	-0.601579000
26	2.946708000	-0.300326000	0.710882000
16	0.931552000	0.524527000	1.795174000
16	1.804205000	-2.178775000	-0.238161000
16	-1.829900000	-2.122574000	0.659477000
16	-1.090063000	-0.022724000	-2.030525000
8	-5.001626000	-1.540192000	-2.447592000
7	3.148753000	1.902684000	0.071748000
7	-3.079026000	1.838490000	-0.504655000
7	-4.000026000	-0.086862000	1.481272000
7	-4.293772000	-1.041004000	-1.601036000
7	4.117784000	-0.637649000	1.940861000
7	4.148459000	-0.469804000	-1.290158000
8	4.738975000	-0.895372000	2.947774000
6	4.183206000	2.506283000	0.941175000
6	-4.391544000	1.337657000	1.547562000
6	1.188603000	2.327458000	1.565713000
6	-3.340124000	2.233561000	0.899122000
6	2.553238000	-2.294469000	-1.911479000
6	-1.804152000	2.436235000	-0.967391000
6	3.578910000	1.945495000	-1.345522000
6	1.867405000	2.629819000	0.234506000
6	-4.188100000	2.276845000	-1.380221000
6	3.231064000	-0.991374000	-2.329006000
6	-5.191401000	-0.944540000	1.630054000
6	4.632637000	0.882256000	-1.643081000
6	5.291756000	-1.384362000	-1.106508000
6	-2.997773000	-0.408300000	2.523859000
6	-1.281828000	1.789060000	-2.244251000
6	-2.422687000	-1.813474000	2.369093000
1	3.877416000	2.425535000	1.985037000
1	5.124887000	1.968113000	0.827786000
1	4.339034000	3.567196000	0.689884000
1	-5.347257000	1.441738000	1.025990000
1	-4.556646000	1.663020000	2.587010000
1	0.226117000	2.847997000	1.593665000
1	1.777373000	2.701013000	2.409400000
1	-2.394301000	2.149456000	1.442153000
1	-3.663519000	3.284552000	0.963566000
1	3.261956000	-3.128376000	-1.922972000
1	1.769536000	-2.525943000	-2.638877000
1	-1.920837000	3.525143000	-1.102836000
1	-1.068129000	2.259428000	-0.177725000
1	2.691756000	1.775340000	-1.962075000
1	3.975148000	2.938138000	-1.611392000
1	2.029233000	3.715622000	0.122413000
1	1.206966000	2.296212000	-0.571441000
1	-4.002118000	1.941412000	-2.401374000
1	-5.125183000	1.828704000	-1.047914000
1	-4.287859000	3.373712000	-1.371598000
1	2.458162000	-0.237045000	-2.501021000
1	3.779724000	-1.138206000	-3.276383000
1	-5.919655000	-0.680412000	0.860600000
1	-4.908823000	-1.988959000	1.488092000
1	-5.654817000	-0.828504000	2.622775000
1	5.534594000	1.072545000	-1.054563000
1	4.922879000	0.943702000	-2.704001000
1	5.959733000	-0.971146000	-0.348115000
1	4.931544000	-2.351986000	-0.752884000
1	5.852199000	-1.528583000	-2.043866000
1	-2.175353000	0.306798000	2.431507000
1	-3.447812000	-0.291463000	3.525974000
1	-0.314585000	2.236445000	-2.493422000
1	-1.949465000	1.970984000	-3.092377000
1	-3.161342000	-2.577265000	2.632005000
1	-1.583391000	-1.921639000	3.062103000
